# The confounding effects of skin colour in photoacoustic imaging

**DOI:** 10.1038/s41467-026-74380-7

**Published:** 2026-06-17

**Authors:** Thomas R. Else, Christine Loreno, Alice Groves, Benjamin T. Cox, Evangelia Vetsiou, Janek Gröhl, Inés Modolell, Sarah E. Bohndiek, Amit Roshan

**Affiliations:** 1 Cancer Research UK Cambridge Institute, University of Cambridge, Cambridge, UK; 2 Department of Physics, University of Cambridge, Cambridge, UK; 3 ACED Clinic Cambridge, Early Cancer Institute, University of Cambridge, Cambridge, UK; 4 Department of Medical Physics and Biomedical Engineering, University College London, London, UK; 5 Cambridge University Hospitals NHS Foundation Trust, Cambridge, UK; 6 Barts Cancer Institute, John Vane Science Centre, Charterhouse Square, London, UK; 7 Present Address: Department of Bioengineering, Imperial College London, London, UK; 8 Present Address: Institute for Experimental Molecular Imaging, RWTH Aachen University Clinic, Aachen, Germany

**Keywords:** Biomarkers, Photoacoustics, Imaging and sensing

## Abstract

Skin colour is known to confound optical devices, adversely impacting care for patients with darker skin. Photoacoustic imaging (PAI) combines optics and ultrasound for deep tissue imaging, creating a complex relationship between PAI-derived biomarkers and skin melanin concentration, yet no generalisable bias correction has been demonstrated. Drawing on a healthy volunteer cohort of 42 participants spanning Fitzpatrick types I–VI and vitiligo – the most diverse ever assembled in PAI – we characterise optical and acoustic mechanisms driving skin colour-dependent degradation in image quality and biomarker quantification. Wavelength-dependent melanin absorption causes spectral colouring, dominating at low melanin levels, while epidermal ultrasound backscattering dominates at high melanin levels, producing a non-linear relationship between unmixed sO₂ and skin tone. Leveraging this understanding, we propose a practical spectral colouring correction and adapt a plane-wave reconstruction algorithm to resolve backscattered ultrasound artefacts. Our findings underscore the need for advanced reconstruction methods to enable equitable clinical PAI.

## Abstract (web summary)

Skin colour is known to confound optical devices, adversely impacting care for patients with darker skin. Here, the authors investigate this effect in photoacoustic imaging by characterising optical and acoustic mechanisms driving skin colour-dependent degradation in image quality and biomarker quantification.

## Introduction

The non-invasive measurement of blood oxygen saturation through the skin with optical technologies, such as pulse oximetry, is now widely acknowledged as being confounded by skin colour^1,2^. Melanin pigmentation in the epidermis strongly absorbs incident light, particularly at shorter wavelengths^3^, which distorts the incident spectrum in a process known as ‘spectral colouring’. In pulse oximetry, given the spectral properties of melanin and oxy- and deoxy-haemoglobin, the dominant effect of light absorption by epidermal melanin is an overestimation of the blood oxygen saturation readout, particularly at dangerously low blood oxygen levels^1^. Such overestimation in pulse oximetry can lead to missed hypoxaemia, which is recognised to have adversely affected patient management globally during the Covid-19 pandemic^4^. Subsequently, several government reviews (e.g., in the United States^5^ and the United Kingdom^6^) have highlighted the need to evaluate potential healthcare inequities in optical technologies that make measurements through the skin, and regulators now require that testing be conducted on participants with a diverse range of skin tones^5^.

**Fig. 1:**
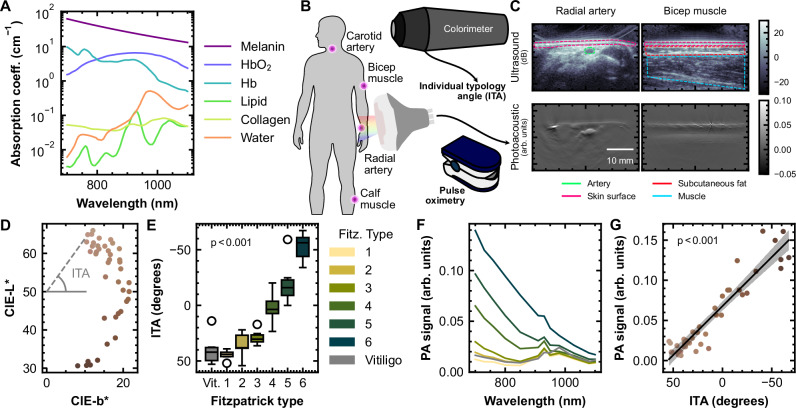
Photoacoustic signal amplitude correlates with subjective and quantitative measurements of skin colour, individual typology angle (ITA) and Fitzpatrick type. **A** Optical absorption spectra of dominant chromophores in the near infrared.^3^ Melanin spectrum shown at 40 % melanosome volume fraction (upper end of physiological levels), haemoglobin spectra are shown at the concentration found in blood. Lipid, collagen and water are shown in their pure forms. **B** Schematic overview of the study, highlighting imaging locations, colorimeter measurement of individual typology angle (ITA) and pulse oximetry. **C** Examples of photoacoustic and ultrasound images in the radial artery and bicep muscles, highlighting key anatomical structures. **D** Plot of colorimeter measurements in CIE colour space, lightness L*, vs yellow-blue b*. **E** ITA from a representative region (neck) correlates with Fitzpatrick type (*n* = 41 individuals, two-sided test, *p* = 5 × 10^−14^, *r*^2^ = 0.77, linear model). The box extends from the first to the third quartile, with a line denoting the median. The whiskers extend to the farthest data point lying within 1.5× the inter-quartile range from the box. The open circles represent values which are more than 1.5 interquartile ranges above the upper or below the lower quartile. **F** The mean photoacoustic signal in a representative location (neck) from the skin surface across Fitzpatrick type groups. **G** 700 nm photoacoustic signal in the skin surface region (neck) correlates with ITA. The shaded area shows 95% confidence intervals for the linear model (*n* = 41 individuals, two-sided test, *p* = 3 × 10^−20^, *r*^2^ = 0.89). Scatter plot markers in (**D**, **G**) are red/green/blue renderings of the colorimeter measurements.

Photoacoustic imaging (PAI) unites light and sound to enable non-invasive deep tissue imaging. Commercially available PAI systems typically exploit the lower scattering of near-infrared light to allow deep tissue imaging of oxy- and deoxy-haemoglobin, lipids, and water (Fig. 1A). Performing PAI at multiple wavelengths enables the optical absorption of these different biomolecules to be resolved and processed into output biomarkers, such as haemoglobin concentration and blood oxygen saturation. PAI has been evaluated across numerous large-scale multi-centre clinical trials and has been shown to hold substantial promise for clinical management of a range of diseases, from inflammatory bowel diseases to breast cancer^7^. Despite recent regulatory approvals in Europe, the USA, and Japan, a comprehensive assessment of the confounding effects of skin colour has yet to be performed for PAI.

The absorption coefficient of melanin in the epidermis can be higher than that of all other contributors for those with darker skin (Fig. 1A). Initial research efforts^8–10^ suggest that skin colour can affect both image quality and biomarker quantification. Image quality impacts primarily appear as ultrasound clutter, increasing in subjects with darker skin and obscuring the desired signal from features like blood vessels. Multispectral analysis is impacted by wavelength-dependent absorption by melanin, which skews spectral unmixing calculations, potentially introducing systematic biases in biomarkers such as blood oxygen saturation^8,11–16^. Increased clutter and spectral colouring could adversely affect image interpretation in the clinic, particularly in applications such as breast cancer, where image feature sets are used to interpret the findings and provide disease staging information^17–19^. Nonetheless, several substantial challenges remain regarding: the generalisation of findings to sites beyond the radial artery, conflicting reports on the quantitative effects of spectral colouring on photoacoustic oximetry biomarkers that imply opposite changes in photoacoustic estimates of blood oxygenation with increasing skin pigmentation^9,11^, the specific origin of the acoustic clutter observed, and the impact of the combined optical and acoustic effects on photoacoustic data.

Accurate quantification in PAI is challenging even without the confounder of melanin^20–24^. The challenge arises because the amount of light absorbed by a molecule, and hence the photoacoustic signal intensity, depends not only on the concentration of each absorbing molecule, but also on the spatially varying light fluence^25^. Inverting this process to obtain molecule concentrations and light scattering coefficients from photoacoustic images is impossible without making several assumptions^26^. To avoid this complexity, an approximate linear spectral unmixing approach^27^ is typically used in clinical applications^28–30^, however, skin pigmentation can distort the calculation. To date, clinical studies have yet to report the impacts of skin tone on their linear spectral unmixing results, nor have they recruited diverse testing cohorts, while emerging preclinical studies of skin tone in PAI show inconsistent results^9,11^.

Here, we present a comprehensive assessment of the confounding effects of skin tone in PAI. We conducted a volunteer study of 42 participants with a diverse range of skin pigmentations and with vitiligo, acquiring data at multiple anatomical sites. We began by analysing the corruption of image quality and biomarker quantification with increasing skin melanin pigmentation. We then undertook detailed theoretical modelling to improve understanding of both the optical and acoustic effects observed. By modelling wavelength-dependent changes in light fluence as a function of skin pigmentation, we demonstrate a fast, practical and generalisable correction method for subjects with lower skin pigmentation. For individuals with very high skin pigmentation, image clutter is shown to originate from the skin surface acting as an ultrasound transducer, with photoacoustic waves generated in the epidermis being backscattered throughout the image field. We adapt a plane wave ultrasound reconstruction algorithm to invert the scattering problem, remarkably reconstructing images that are practically identical to conventional ultrasound images. We underscore the importance of PAI clinical trials testing on diverse populations and accelerating co-design of hardware and software to ensure equitable future application of PAI in the clinic.

## Results

### A diverse healthy volunteer study was vital to understand confounding effects of skin colour in photoacoustic imaging

In our healthy volunteer study, we pre-screened potential participants using a questionnaire to establish their Fitzpatrick type, enabling fast and easy remote self-assessment of their qualitative skin tone grouping^31^. These self-reported characteristics were confirmed by an expert in skin oncology (AR) with a clinical practice in melanoma skin cancer associated with diverse skin pigmentation. To avoid other potential confounders, we sought to balance age, sex and body mass index between groups (summarised in Table 1, full data in Supplementary Table S1, S2). We recruited 6 participants from each Fitzpatrick type and 6 people with vitiligo for a total of 42 participants. Data from one participant was excluded, as ultrasound data was unavailable.

**Table 1:** Summary of the study cohort. Numerical values are shown as mean ± standard deviation, with range in brackets

Fitzpatrick Type	Age (years)	Sex	BMI (kg m^−2^)	ITA (°)
I	53.5 ± 22.2 (24, 76)	1 M, 5 F	21.2 ± 1.4 (19.1, 23.1)	44.8 ± 4.0 (40.2, 50.9)
II	46.2 ± 21.4 (24, 73)	3 M, 3 F	24.5 ± 4.0 (18.5, 29.9)	36.5 ± 11.2 (21.6, 54.1)
III	31.7 ± 6.4 (25, 43)	2 M, 4 F	23.6 ± 3.3 (19.7, 27.7)	29.2 ± 7.0 (17.0, 36.8)
IV	34.0 ± 12.9 (22, 57)	3 M, 3 F	23.3 ± 2.6 (19.6, 27.2)	2.2 ± 14.0 (− 19.1, 22.6)
V	37.5 ± 15.2 (23, 65)	3 M, 3 F	23.8 ± 2.8 (20.2, 27.5)	− 21.4 ± 22.4 (− 63.5, − 0.8)
VI	34.0 ± 9.5 (26, 48)	4 M, 2 F	23.8 ± 3.2 (19.6, 27.5)	− 53.0 ± 13.4 (− 67.7, − 33.8)
Vitiligo	59.7 ± 12.7 (40, 72)	2 M, 4 F	25.0 ± 4.1 (20.2, 28.7)	39.1 ± 13.3 (14.0, 51.2)

A total of six participants in each group (row). The ITA measurement shown is indicative of a sun-exposed region (the neck).

All participants underwent photoacoustic imaging (PAI) with simultaneous reflection ultrasound (US) computed tomography and colourimetry at each imaging site, as well as systemic pulse oximetry (Fig. 1B). The photoacoustic probe was positioned using the anatomical information from the ultrasound image and data acquisition was performed for 30 s per site (Fig. 1C). To maximise generalisability of findings to a broad range of clinical applications, we acquired PAI data in several anatomical sites, targeting major blood vessels and muscles, across 13 wavelengths, from 700 nm to 1110 nm (see Methods).

Colorimeter measurements were mapped to individual typology angle (ITA), a widely used measure of skin melanin level, which decreases with increasing melanin concentration (Fig. 1D). Separate ITA measurements were made for each imaging site in each participant, including a sun-exposed region (the neck), the forearm, the bicep and lower leg. By transforming ITA to melanosome volume fraction (see Methods), we see that our recruitment resulted in an approximately uniform distribution of the logarithm of epidermal melanin concentration (Supplementary Fig. S1). A strong correlation was observed between the sun-exposed ITA and all other sites, however, the bicep and forearm had consistently higher ITA values than the neck (*p* < 0.001 and *p* = 0.004, respectively), particularly at the lowest ITA values, reflecting the reduced melanin levels in non-sun exposed regions (Supplementary Fig. S2). ITA in the leg was consistently higher than the neck in participants with high ITA values, but was more similar at low ITA values, reflecting differences in hair follicle colour (Supplementary Fig. S2). The neck ITA decreased significantly with increasing Fitzpatrick type (*p* < 0.001 with a linear model), as observed in prior studies (Fig. 1E)^32,33^. The significant differences in ITA between imaging sites demonstrated the importance of a site-dependent quantitative measurement of skin colour in our subsequent analyses.

We confirmed the relationship between photoacoustic signal from the skin surface and skin pigmentation quantified by colorimetry^8,11^. Interestingly, we observed that the photoacoustic time series signal amplitude reached the upper limit of the analogue to digital converter in subjects with ITA as high as 40 (Supplementary Fig. S3), resulting in clipping; band-pass filtering was applied prior to image reconstruction to reduce the effect. After model-based image reconstruction, the photoacoustic signal in the skin was obtained by drawing a polygonal region of interest (ROI) on the US images and applying it to the photoacoustic images (Fig. 1C). The skin PA spectra showed the characteristic decrease with increasing wavelength associated with melanin absorption (Fig. 1F) and the PA signal at 700 nm correlated strongly with ITA (*p* < 0.001, linear model; Fig. 1G). The correlation between PA signal and ITA was still observed when high (> 10) and low ( < 10) ITA values were considered separately (Supplementary Fig. S4, *p* = 0.018 and *p* < 0.001, respectively).

### Increasing skin pigmentation leads to extensive acoustic clutter artefacts

**Fig. 2:**
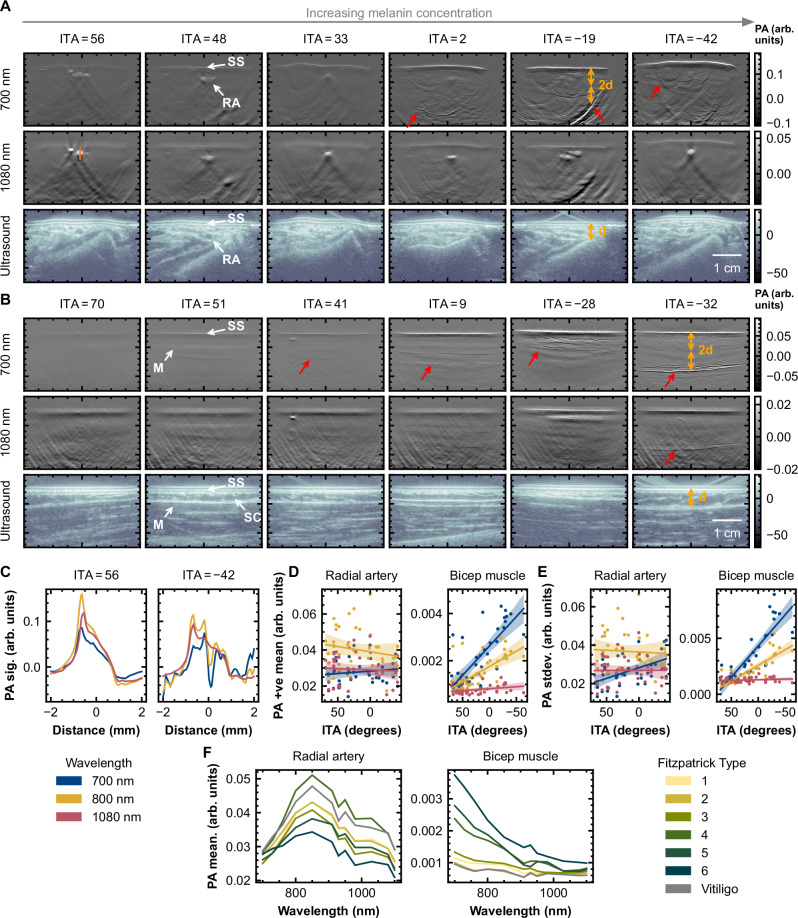
Single-wavelength photoacoustic data reveal increasing clutter signal with increasing skin pigmentation. Example ultrasound and photoacoustic images of the radial artery (**A**) and bicep muscle (**B**) at two wavelengths across a range of skin pigmentations, as measured with individual typology angle (ITA). White arrows label anatomical features: skin surface (SS), radial artery (RA), muscle (M), subcutaneous fat (SC). Red arrows show imaging artefacts; orange arrows demonstrate the depth, d, of anatomical structures and their corresponding artefacts. **C** Vertical lines plots of PA signal intensity through the radial artery in the subjects with the maximum ITA (least melanin, left) and minimum (most melanin, right) at several wavelengths. Mean, excluding negative pixels, (**D**) and standard deviation, including positive and negative pixels, (**E**) of the photoacoustic signal in the radial artery and bicep at several wavelengths as a function of ITA. Each point corresponds to one individual. The solid line shows a linear regression with a shaded 95 % confidence interval. Wavelengths shown in (**C**–**E**) are 700 nm (blue), 800 nm (orange) and 1080 nm (pink). **F** Mean photoacoustic spectrum (excluding negative pixels) for each Fitzpatrick type group and the vitiligo cohort in the radial artery and bicep muscle regions.

We first qualitatively assessed the appearance of the image features and artefacts across the dataset. In forearm scans, the radial artery was clearly distinguished at long wavelengths (>= 950 nm) in all participants but was only visible at all wavelengths in participants with lighter skin tones (Fig. 2A). Conversely, as noted previously^8^, the radial artery was entirely obscured by clutter in participants with darker skin, particularly at shorter wavelengths (Fig. 2A). Unlike prior work where clutter showed a speckle-like appearance^8^, here we note the presence of very dark or very light arc-shaped features below the skin surface in the images. Likewise, in scans of the bicep and calf muscles (Fig. 2B and Supplementary Fig. S5), photoacoustic signals can distinguish the muscle from the subcutaneous fat in participants with lighter skin, but acoustic clutter comes to dominate in participants with darker skin.

A bright horizontal artefact, located twice as deep as the muscle-fat interface, was observed in all muscle scans in subjects with darker skin, consistent with strong acoustic reflection of the photoacoustic wave at the muscle-fat interface (Fig. 2B and Supplementary Fig. S5). Similar observations were made in scans of the neck, where veins around the carotid artery can be seen in photoacoustic images in almost all subjects, even at shorter wavelengths, but increased clutter is visible in subjects with darker skin, obscuring the carotid artery (Supplementary Fig. S6).

Our qualitative assessment suggested that the forearm and bicep muscles were informative sites for understanding the confounding effects of skin colour, due to the presence of the radial artery as a focal feature, and the striking presence of acoustic reflection artefacts in the muscle images. The remainder of the manuscript, therefore, focuses primarily on the analysis of the radial artery in the forearm and the bicep muscle. Data from other sites are presented for completeness in the Supplementary Information.

We then performed a quantitative assessment of photoacoustic signals in the radial artery (forearm site) and muscle (bicep site). A vertical line plot through the radial artery illustrates the effects of acoustic clutter; in subjects with lighter skin, the radial artery has a consistent shape at all wavelengths, with sharply defined edges, and a slight decay in intensity consistent with reducing light fluence (Fig. 2C). In subjects with darker skin, distinct oscillations could be observed in the line plots through the radial artery (Fig. 2C) due to stripe artefacts in the photoacoustic image and bands of low signal intensity.

The overall signal intensity of different structures did not decrease with increasing skin pigmentation, as might have been predicted based solely on optical considerations. Rather, in the radial artery, there was a negligible change in signal when artefactual negative pixels are excluded at several wavelengths used for blood vessel imaging in commercial devices (Fig. 2D, less than 1.3-fold changes, not statistically significant at 700 nm, 800 nm and 1080 nm from ITA = 68 to ITA = − 42). However, the mean signal intensity and corresponding contrast to noise ratio at shorter wavelengths in the radial artery decreased significantly with increasing melanin level when negative pixels were included (Supplementary Fig. S7). At shorter wavelengths ( < 950 nm), the contrast to noise ratio of the radial artery was significantly higher in participants with low ITA values (*p* < 0.01, linear model), while at longer wavelengths (≥ 950 nm), the contrast to noise ratio of the radial artery did not vary significantly (*p* > 0.1, linear model) with ITA. Quantification with negative pixels included is therefore consistent with predictions made based on optical considerations alone and aligns with a qualitative assessment of the images; oscillatory clutter, as seen in Fig. 2B, appears to cancel when negative pixels are included.

The standard deviation of the signal across the radial artery increased significantly at 700 nm, reflecting the presence of clutter artefacts with increasing melanin (Fig. 2E, *p* < 0.001 at 700 nm, *p* = 0.70 at 800 nm, *p* = 0.97 at 1080 nm). The averaged photoacoustic spectrum of the radial artery had substantial variability across Fitzpatrick types, maintaining the broad shape associated with oxygenated blood, with a slight increase at low wavelengths at the highest Fitzpatrick types (Fig. 2F).

In the bicep, there was a dramatic increase in mean photoacoustic signal with increasing skin pigmentation, consistent with the line-shaped clutter observed in the images (4.8-fold from ITA = 70 to ITA = − 63 and *p* < 0.001 at 700 nm, 3-fold and *p* < 0.001 at 800 nm, 1.3-fold and *p* = 0.08 at 1080 nm, Fig. 2D). A significant increase in signal standard deviation was observed with increasing skin pigmentation in the bicep muscle, again reflecting increased clutter (*p* < 0.001 at 700 nm, *p* < 0.001 at 800 nm, *p* = 0.29 at 1080 nm, Fig. 2E). The overall photoacoustic spectrum across the muscle varied dramatically across Fitzpatrick types, moving from a relatively flat spectrum to one with substantial wavelength dependence, resembling the optical absorption spectrum of melanin (Fig. 2F).

### Estimates of blood oxygen saturation are confounded by skin pigmentation

**Fig. 3:**
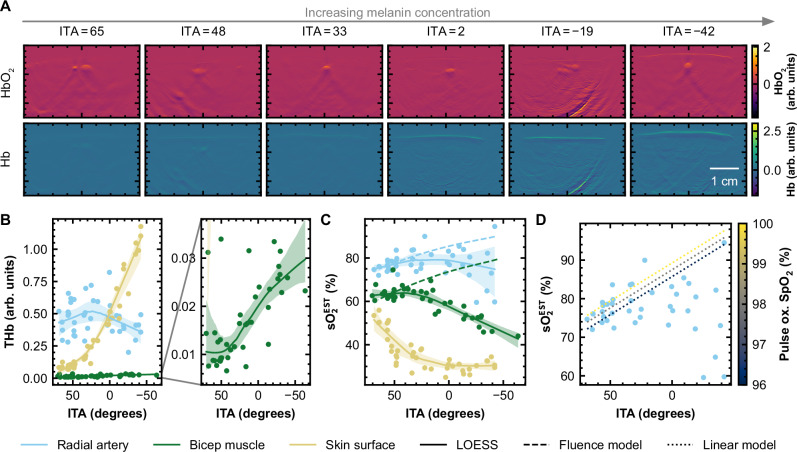
Linear spectral unmixing results in regions of interest show a strong dependence on skin pigmentation. **A** Unmixed oxy-haemoglobin (HbO_2_) and deoxy-haemoglobin (Hb) images of six participants across the range of skin pigmentations. Unmixed total haemoglobin (**B**) and blood oxygenation (sO_2_^EST^ **C**) variation in the radial artery, bicep muscle and skin surface regions of interest. LOWESS fit mean, and 95% confidence intervals are shown as solid lines and shaded regions, respectively. A light fluence model relative to the minimum melanin level is shown as a dotted line (**B**, **C**). **D** A linear mixed effects model fit over the linear skin colour range (ITA > 10) shows the unmixed sO_2_^EST^ correlation with pulse oximetry. Dotted lines show the linear mixed effects model at 96, 98 and 100 % SpO_2_.

We next applied linear spectral unmixing for oxy- (HbO_2_) and deoxy-haemoglobin (Hb) to our dataset at wavelengths between 700 nm and 900 nm and estimated both total haemoglobin (THb = HbO_2_ + Hb) and blood oxygen saturation (sO_2_^EST^ = HbO_2_ / THb) as commonly evaluated PAI biomarkers in the clinic. Encouragingly, HbO_2_ images showed blood vessels quite clearly, even in subjects with darker skin (Fig. 3A upper row). On the other hand, Hb images showed a high level of clutter with increasing melanin and poor blood vessel visibility (Fig. 3A lower row).

By extracting average values across ROIs, we were able to identify several important trends that indicate challenges in the clinical translation of PAI biomarkers. Estimates of total haemoglobin in the skin surface ROI increased dramatically as ITA decreased, highlighting a limitation of linear unmixing – melanin absorption can be mistaken for deoxy-haemoglobin – leading also to a decrease in sO_2_^EST^ with decreasing ITA (Fig. 3C). In the radial artery, there was a negligible change in THb with ITA (*p* = 0.29), but substantial variability between subjects (Fig. 3B). At low melanin levels (high ITA values), sO_2_^EST^ increased with decreasing ITA (*p* = 0.011, *n* = 25 subjects ITA > 10), while at high melanin levels (lower ITA values), the trend reverses and there is substantial variability between subjects (*p* = 0.39, *n* = 16 subjects ITA < 10) (Fig. 3C). In subjects with lower melanin levels (ITA > 10), sO_2_^EST^ correlated with pulse oximetry measurements (Fig. 3D, *p* = 0.02, coefficient = 0.885, [95 % CI 0.14, 1.63], linear mixed effects model, 163 observations from 18 participants).

In the bicep muscle, there was a dramatic increase in THb with increasing melanin level (*p* < 0.001, linear model), consistent with the increase in clutter signal observed in single wavelength data (Fig. 3B zoomed graph). Again, sO_2_^EST^ follows a more complex trend, staying approximately level with increasing melanin at low melanin levels (ITA > 10, *p* = 0.57), before decreasing significantly at high melanin levels (ITA < 10, *p* < 0.001, [95 % CI 0.132, 0.306]; Fig. 3C).

To establish the basis for the dependence of sO_2_^EST^ on skin colour, we simulated the effect of spectral colouring from melanin on light fluence and the consequent effect on PAI sO_2_^EST^ (see Methods). The model had one free parameter, the sO_2_^EST^ at zero melanin concentration, which was dependent on the non-melanin absorption and scattering of the surrounding tissue. The theoretical model was found to fit very well at low melanin levels (ITA ~ > 30) in the bicep muscle and the radial artery (shown as a dashed line on Fig. 3C). Our fluence model fits particularly well for the radial artery, with a likelihood ratio of 0.016 compared to a constant model (ITA > 30). For the bicep muscle, the model is not statistically any better than a constant model (likelihood ratio of 2.1), but is not inconsistent either (2-tailed *t* test on residuals *p*-value = 0.98; ITA > 30). The model gradually diverges from the experimental data as melanin concentration increases, suggesting that spectral colouring ceases to be the dominant process at high melanin levels (Fig. 3C).

### Reproducibility was partially influenced by participant skin tone

To assess the potential impact of skin pigmentation on the reproducibility of photoacoustic measurements, we evaluated the coefficient of variation of single-wavelength measurements and unmixed coefficients sO_2_^EST^ and THb across three scans, with replacement, in all participants. For reference, a vendor-provided phantom was scanned prior to scanning each volunteer. The mean signal inside each phantom inclusion was evaluated over time, and the coefficient of variation was calculated (Supplementary Fig. S8). In the phantom inclusion with the highest photoacoustic signal intensity, which is of the same order of magnitude as measurements in the radial artery, the coefficient of variation was below 10% for all wavelengths below 1000 nm, and no higher than 11.5% for any wavelength. The mean coefficient of variation across all wavelengths from 680 nm to 1100 nm, inclusive, was 6.8%.

In measurements of the radial artery, there was a very slight decrease in the coefficient of variation with increasing melanin level (*p* = 0.019 and *r* = 0.41 at 800 nm, *p* = 0.049 and *r* = 0.35 at 1080 nm, Spearman’s rank (SR) test, Supplementary Fig. S9A,B upper row). In the bicep muscle, there was no significant correlation between ITA and coefficient of variation (Supplementary Fig. S9A,B lower row). Overall, across all wavelengths, there was a mean coefficient of variation of 22 % in the half of the cohort with lighter skin and 14 % in the half with darker skin. For THb, a similar observation was made, with a slight correlation between coefficient of variation and ITA in the radial artery (*p* = 0.021, *r* = 0.39, SR test) and no correlation in the bicep muscle (*p* = 0.50, *r *= 0.12, SR test) (Supplementary Fig. S10). Coefficient of variation in sO_2_^EST^ correlated significantly with ITA in the radial artery (*p* = 0.029, *r *= − 0.37, SR test), but there was no trend in the bicep muscle data (*p* = 0.56, *r* = − 0.10, SR test; Supplementary Fig. S10). In the radial artery, the mean coefficient of variation for THb and sO_2_^EST^ was 25% and 3.3%, respectively, for the lighter half of the cohort and 17% and 5.5% for the half with darker skin. In the bicep muscle, the mean coefficient of variation for THb and sO_2_^EST^ was 24% and 4.2%, respectively, for the lighter half of the cohort and 27% and 5.3% for the half with darker skin.

### Photoacoustic imaging is sensitive to subtle changes in skin pigmentation in participants with vitiligo

**Fig. 4:**
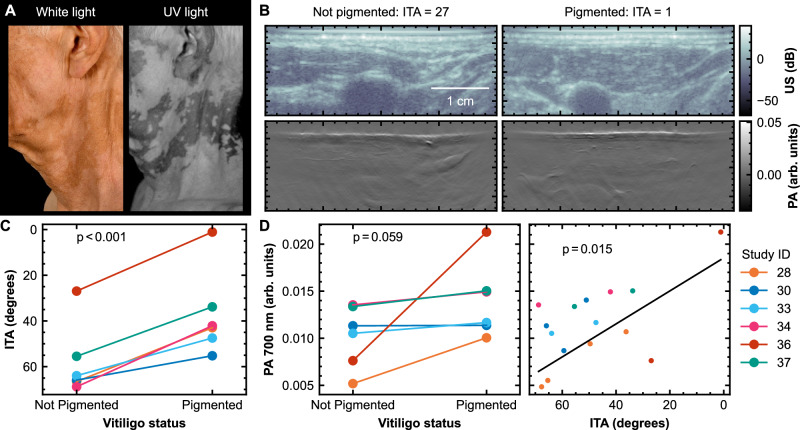
Photoacoustic imaging in subjects with vitiligo reveals high sensitivity to melanin concentration in the epidermis. **A** Example white light and UV light photographs of the neck of a participant with vitiligo. **B** Ultrasound (US) and 700 nm photoacoustic images of the neck (carotid artery) over a non-pigmented patch and a pigmented patch of the same participant. **C** Individual typology angle for each vitiligo participant over vitiligo patches (not pigmented) and patches with normal pigmentation (pigmented). *p* = 1.2 × 10^−11^. **D** Mean 700 nm photoacoustic signal in the epidermis in vitiligo patches (not pigmented) and patches with normal skin pigmentation (pigmented). Plot of the same 700 nm photoacoustic signal against ITA with a linear mixed effects model line of best fit (black). All *p*-values shown were calculated using a linear mixed effects model and use a two-sided test. Each point represents aggregated measurements for a single individual with the given study ID at a particular imaging site.

Photoacoustic images from participants with vitiligo were compared between pigmented and non-pigmented (vitiligo affected) regions, as determined by white light and UV photography (Fig. 4A). All participants in the vitiligo cohort would otherwise have been placed in Fitzpatrick types I, II, or III, so there is relatively low variation in skin pigmentation in the cohort. Consequently, while increases in melanin absorption of the epidermis between pigmented regions and non-pigmented can be seen in 700 nm photoacoustic images, no changes in the influence of image artefacts can be observed (Fig. 4B). ITA values had a highly significant difference between pigmented and non-pigmented patches (*p* < 0.001, linear mixed effects model; Fig. 4C). Photoacoustic signals from the skin surface region at the lowest wavelength of 700 nm (highest melanin absorption) increased only modestly between non-pigmented regions and pigmented regions, because of the relatively limited range of skin tones in the vitiligo study cohort and because of absorption from other chromophores like haemoglobin (*p* = 0.059, linear mixed effects model; Fig. 4D). There was a high correlation, however, between ITA and 700 nm skin surface photoacoustic signal, as observed in the wider cohort (*p* = 0.015, linear mixed effects model; Fig. 4D).

### A fast generalisable method can correct for melanin spectral colouring and improve estimation of blood oxygen saturation in participants with low skin pigmentation

**Fig. 5:**
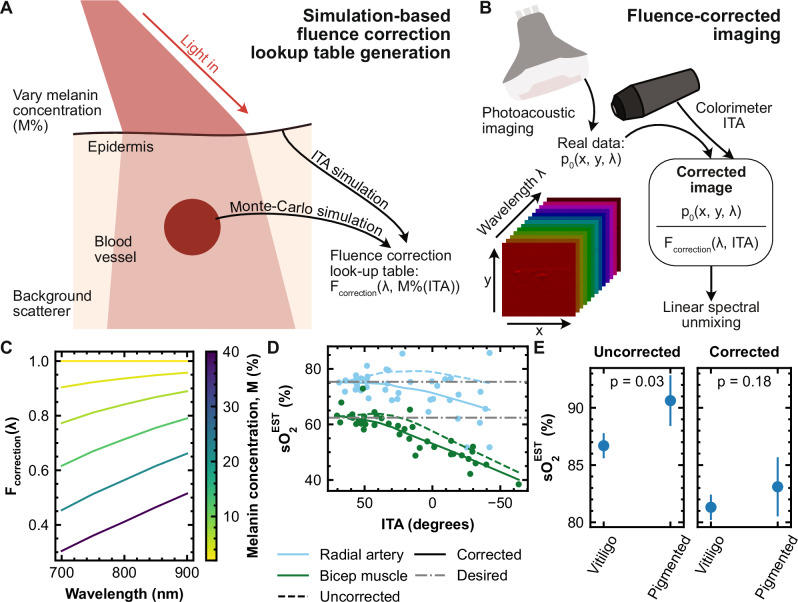
A Monte-Carlo simulation model for spectral colouring correction due to melanin in the epidermis. **A** A schematic of the approach taken to fluence correction, where melanosome volume fraction (M %) is related to light fluence (F), wavelength (λ), and individual typology angle (ITA) through optical modelling. **B** A schematic of how the fluence correction is applied to the real photoacoustic data. **C** The normalised fluence correction factor as a function of wavelength and melanosome volume fraction (M %). **D** Blood oxygenation estimates from linear unmixing in the radial artery and the bicep muscle of healthy volunteers before and after fluence correction, with a LOWESS trend line. **E** Blood oxygenation estimates from linear unmixing of the carotid artery from a single participant with vitiligo over a non-pigmented (vitiligo) patch and a pigmented patch, before and after correction; *p*-values show a 1-tailed *t* test (*n* = 3 technical replicates per group). The error bars show mean ± 2 x SEM.

To mitigate the spectral colouring component of skin colour bias, we developed a fluence correction approach. We modelled light transport through a simple tissue model consisting of an epidermis with varying melanosome volume fraction, a blood vessel and background scattering and absorbing tissue (Fig. 5A; see Methods). The relationship between melanosome volume fraction and ITA was established in a previous study, enabling us to connect the simulated data to our in vivo measurements^11^. The simulated illumination geometry was consistent with the clinical PA system used in this study. The simulated wavelength was varied, and the light fluence in the blood vessel was averaged to give a lookup table. The lookup table was normalised by the lowest melanin concentration simulated, and interpolated across wavelength and ITA, giving a correction factor that could be applied to the clinical data (Fig. 5B). The correction factor did not vary spatially, so was calculated by a simple cubic spline interpolation of the lookup table (Fig. 5C).

The correction approach was then tested on the reconstructed photoacoustic images acquired in healthy volunteers, which were divided by the associated correction factor to give a corrected image. Linear spectral unmixing was then applied to estimate blood oxygenation (Fig. 5D). The increase in sO_2_^EST^ with increasing skin pigmentation associated with spectral colouring was normalised in the corrected images at low melanin levels. The correction method also worked well for a matched region on a vitiligo participant and was able to match the sO_2_^EST^ across their vitiligo (ITA = 26.9°) and pigmented (ITA = 1.1°) regions (*p* = 0.03 before correction, *p* = 0.18 after correction, Fig. 5E). The correction factor removed the statistically significant relationship between ITA and sO_2_^EST^ of the radial artery in subjects with ITA > 10 (*p* = 0.82). After applying the correction factor, sO_2_^EST^ in the bicep muscle did not have a significant ITA dependence for ITA > 30 (*p* = 0.42). Unfortunately, this did not compensate for biases observed at higher melanin levels, suggesting that spectral colouring cannot explain the observed trends alone.

Given the strong correlation between ITA and photoacoustic signal (Fig. 1G), we derived a fluence correction factor based purely on the mean photoacoustic signal from the skin surface. Based on a relationship between ITA and melanosome volume fraction derived in prior work and photoacoustic signal averaged across the skin surface region, we created a linear model to map photoacoustic signal to melanosome volume fraction (p < 0.001, *r*^2^ = 0.90, Supplementary Fig. S11A)^11^. As with the ITA-based approach, we were able to use the same lookup table approach to correct for spectral colouring in the radial artery (ITA > 10, *p* = 0.63) and bicep muscle (ITA > 30, *p* = 0.85) regions of interest, although again this is only for higher ITA values (Supplementary Fig. S11B).

### Acoustic clutter arises from the epidermis, which acts as a secondary source of ultrasound in participants with high skin pigmentation

**Fig. 6:**
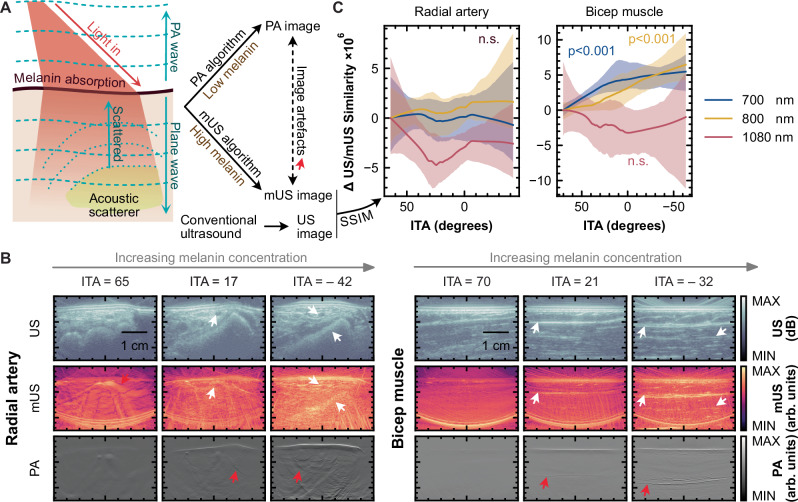
Plane wave ultrasound reconstruction illustrates that ultrasound scattering artefacts dominate in participants with darker skin pigmentation at shorter wavelengths. **A** A schematic showing the generation of ultrasound plane waves in the skin surface, which scatter off deeper scattering tissues, like bones and muscles. A standard photoacoustic (PA) algorithm generates a good PA image, whilst the melanin ultrasound (mUS) algorithm generates an ultrasound (US) image at high melanin levels. At the other extreme, mUS and PA images contain artefacts (shown with a red arrow). **B** Photoacoustic (PA), melanin ultrasound (mUS) and conventional ultrasound (US) images at low, medium and high melanin levels from the radial artery (left) and bicep muscle (right). Features visible in both mUS and US images are highlighted with a white arrow, while artefacts are highlighted by a red arrow. **C** A plot of a LOWESS fit of the structural similarity index metric (SSIM) change between mUS and conventional US images as a function of individual typology angle (ITA) in all subjects at several wavelengths, relative to the value at maximum ITA; shaded areas show 95 % confidence intervals for the LOWESS fit, the solid line shows the mean of the LOWESS fit. Quoted p-values use a two-sided Spearman’s rank test. Radial artery: 700 nm, *p* = 0.94; 800 nm, *p* = 0.66; 1080 nm, *p* = 0.62. Bicep: 700 nm, *p* = 8 × 10^−5^; 800 nm, *p* = 6 × 10^−4^; 1080 nm, *p* = 0.41.

Having established how changes in the skin pigmentation affect the propagation of light in the tissue and subsequently corrupt estimation of THb and sO_2_^EST^, we finally sought to develop a deeper understanding of the origin of the clutter artefacts observed. An initial qualitative evaluation suggested that the artefacts appeared in the PA image approximately twice as far below the skin surface as strongly reflecting structures in the ultrasound image, with spectral features similar to that of melanin in the skin surface (Fig. 2B, D and Supplementary Fig. S5). The consistent appearance aligned with prior evidence that the epidermis could be acting as a secondary source of acoustic waves due to the strong optical absorption of melanin, which were backscattered at interfaces between tissues of different acoustic impedance^14–16,34,35^. Based on the wave travel time, these reflections are erroneously reconstructed in the photoacoustic image (Fig. 6A). Due to the optical absorption spectrum of melanin and the limitations of linear spectral unmixing, such artefacts could easily be mistaken as low-oxygenation blood vessels, which could be highly misleading in a clinical assessment.

To further illustrate the source of acoustic clutter, we developed an algorithm that was able, in the high melanin limit, to recover the spatial ultrasound scattering distribution from the clutter. The resulting images are referred to as melanin ultrasound (mUS). We adapted an ultrasound reconstruction algorithm originally used for plane wave ultrasound^36^, making changes to account for the arc-shaped transducer and the spatial offset between the detector and the epidermis source of ultrasound (see Methods). We validated our algorithm with computational mUS and US simulations, showing that mUS images resemble conventional US in the high melanin limit (Supplementary Fig. S12).

Remarkably, mUS images computed from 700 nm PA time series data resemble conventional ultrasound images in all imaging sites, even at only modest levels of melanin (Fig. 6B). At very high melanin levels, deep anatomical structures, such as the radius bone, striations in the muscles, and the carotid artery, can be visualised in mUS images (Fig. 6B and Supplementary Figs. S13, S14). By contrast, mUS images at low melanin levels contain artefacts from features like blood vessels and muscles, as the photoacoustic wave generated in structures like the radial artery and bicep muscle outweighs the secondary melanin absorption wave (Fig. 6B and Supplementary Figs. S13, S14). The structural similarity index metric (SSIM) was calculated to compare mUS and US images (Fig. 6C). At shorter wavelengths, SSIM shows a significant correlation with ITA in the bicep muscle (*p* < 0.001 for 700 nm and 800 nm, *p* = 0.48 for 1080 nm, Spearman’s rank test), but not in the radial artery (*p* > 0.5 at 700, 800 and 1080 nm, Spearman’s rank test). Our observations demonstrate that the epidermis acts as a strong source of ultrasound waves, overwhelming primary PA signals at high melanin concentrations, imprinting artefacts with a melanin spectral fingerprint on PA images. Thus, our previous observations of sO_2_^EST^ diverging from the fluence model at high melanin levels can be attributed to acoustic heterogeneity, a factor not included in the PAI reconstruction model, which assumes no ultrasound scattering.

## Discussion

PAI holds potential for adoption in clinical indications ranging from peripheral artery disease assessment to cancer diagnosis, but the confounding effects of skin colour on PAI quality and biomarker measurements have not yet been well established. We carried out a healthy volunteer study in 42 participants across a wide range of skin tones, including 6 participants with vitiligo. We showed that single wavelength measurements and the linear unmixing quantities THb and sO_2_^EST^ are all affected by skin pigmentation in a non-linear manner due to confounding effects arising from both optical (spectral colouring) and acoustic (backscattering) effects. We revealed the physical mechanisms behind the observed effects and took steps towards correction methodologies through comprehensive computational modelling.

To address the spectral colouring effects, we related a computational model of light transport to an independent measure of skin colour, ITA, and were successfully able to predict the changing light fluence spectrum below the epidermis, giving improved quantitative oximetry values at low melanin levels. To better understand the acoustic effects, we modelled the epidermis as a source of ultrasound plane waves, allowing us to reconstruct ultrasound images from the clutter signal. Our work paves the way for skin colour-independent clinical photoacoustic imaging, addressing some challenges but revealing the difficulties of tackling this issue in a universal manner.

Our observations suggest that optical and acoustic phenomena separately contribute to photoacoustic skin tone dependence. Optical effects from melanin absorption change the light fluence in deeper tissue regions, like muscles or blood vessels, giving a misleading spectral appearance. At lower melanin levels (ITA ~ > 10), our observations are consistent with previous theoretical work, in which optical absorption by melanin in the epidermis leads to a large reduction in light fluence at shorter wavelengths and less so at longer wavelengths, predicting a decrease in photoacoustic signal intensity and an increase in unmixed blood oxygenation with increasing melanin^11^. Yet the optical model breaks down at higher melanin levels. For ITA less than ~ 10, we saw a rise in the single-wavelength photoacoustic signal and a decrease in unmixed blood oxygenation. We were able to attribute the divergence from our optical model to backscattering of photoacoustic ultrasound generated in the epidermis, and the constant speed of sound assumption in image reconstruction. The resulting artefacts have the same spectrum as melanin and obscure the desired primary photoacoustic signal.

Having observed these confounders, we then sought to correct them. We showed that a light fluence model can compensate for skin colour corruption of sO_2_^EST^ in subjects with ITA greater than ~ 10. The correction method did not depend on any empirical parameters; we directly modelled the illumination geometry and individual typology angle as a function of melanosome volume fraction. We chose not to derive an empirical correction factor for sO_2_^EST^, as proposed previously^9^, as differences in tissue acoustic properties of the dataset used to generate the empirical model would alter the clutter level, leading to more or less spectral corruption in different anatomical sites. Our proposed correction model therefore generalises to other tissue types, like muscles, while previous empirical formulae were derived specifically for the radial artery^9^. The model also takes ITA as its input, rather than a qualitative factor, like Fitzpatrick type, allowing for skin colour differences within the same subject to be modelled, neatly demonstrated by data in a subject with vitiligo. The lookup table approach would easily integrate with existing clinical software without demanding computational overheads or even the need for a colorimeter, albeit still only addressing the optical challenge.

Acoustic scattering means that photoacoustic images of participants with higher melanin pigmentation (ITA < ~ 10) are not, in fact, what we usually think of as photoacoustic images. Rather, the time series data is far more like a plane wave ultrasound scan. Because of the high optical absorption of melanin, the epidermis acts as an ultrasound transmitter, emitting waves into the deeper tissue, which give rise to reflected ultrasound signals that are far stronger than the desired primary photoacoustic signal from the tissue of interest^12^. Remarkably, we were able to generate excellent ultrasound images from the PA time series data, convincingly illustrating how dominant backscattering is. Unfortunately, this mapping approach does not correct for the confounder. Previous studies have proposed methods for reducing backscattering but require either specialist hardware or contrived imaging protocols to cancel the scattered wave^12,15,16^, or introduce a non-linear scaling to the intensity, making quantification impossible^8^. Rather, our approach demonstrates the severity of the problem and indicates ways in which it might be addressed in future work. For example, the ultrasound reflectivity of the tissue derived from photoacoustic time series data could be incorporated into a model to separate out the primary photoacoustic signal from the scattered signals. Similar challenges are observed in other areas of photoacoustic imaging with strong ultrasound scattering, such as in the brain, where jointly reconstructing the initial pressure and speed of sound have shown promise^37^. Furthermore, since the scattered signal has the same wavelength dependence as the epidermis, a spectral unmixing-like approach might enable some bias reduction.

This is the largest healthy volunteer study of its kind so far in PAI, giving several key insights into the phenomenon of skin colour bias in photoacoustic imaging. There remain, however, limitations in recruitment and in the applicability of our proposed correction method that should be addressed in future work. Our recruitment was based on the Fitzpatrick scale, giving approximately uniform sampling in terms of the logarithm of melanosome volume fraction. Alternative qualitative metrics, such as the Monk scale, may have enabled more uniform recruitment with respect to skin type without increased complexity in the recruitment process^38^. Furthermore, while melanin is the clearest example of skin pigmentation variation in the population, additional variation, for example, scar tissue or darker areolar skin in breast imaging, also warrants additional investigation. Representative recruitment for the vitiligo cohort was also challenging; we were only able to recruit subjects with vitiligo with Fitzpatrick types I-III. It should further be emphasised that our proposed correction method, while promising, does not address acoustic backscattering effects and thus breaks down for participants with darker skin. Truly equitable photoacoustic imaging would require both effects to be jointly corrected and remains an open challenge.

The study was carried out only with a single photoacoustic imaging system, with an arc-shaped detector, 2D acquisition geometry and a relatively wide detection bandwidth. How these results would translate to different systems, particularly those with different illumination and acoustic detection hardware, remains unclear. In particular, the clutter signal will depend on transducer characteristics, such as bandwidth, noise level, and optical power and pulse duration, which vary substantially between LED and laser illumination hardware^39^. Likewise, the lookup table generated for melanin absorption fluence correction will depend on the illumination geometry and should be recalculated and validated on other photoacoustic systems before it can be used in clinical practice. Furthermore, this study did not modulate the photoacoustic signal, as sometimes done in clinical photoacoustic applications, for example, with a blood pressure cuff or exercise. One would expect that “delta” metrics would be less susceptible to skin colour bias, as artefacts would remain relatively constant over the perturbation.

The competing effects of the acoustic and optical confounders could have profound implications for clinical translation of photoacoustic imaging. Either confounder may dominate, making blood vessels appear more or less oxygenated than they truly are depending on the subject’s precise skin colour. Perhaps most concerningly, artefacts may resemble hypoxic blood vessels, whose intensity depends not primarily on optical properties as we tend to expect, but on acoustic properties like density and elasticity of the surrounding tissue. Such confounders, if not accounted for, would have alarming consequences in applications like breast cancer diagnosis, for which regulatory approval has already been achieved, and where blood oxygenation is a key marker of cancer stage^18,19^. It is incumbent on the photoacoustic community to tackle this problem before widespread adoption of the technology.

## Methods

To examine the effects of skin colour on PAI, a prospective, exploratory observational trial was conducted (PAISKINTONE). PAISKINTONE was approved by the East of England - Cambridge South Research Committee not (ref: 23/EE/0019) and conducted in accordance with the Declaration of Helsinki. Written informed consent was obtained from all study participants. The trial was registered with clinicaltrials.gov: https://clinicaltrials.gov/study/NCT05554523. The study started in June 2023 and finished recruitment in August 2024. A total of 42 participants were recruited for the study. We sought to balance age, sex and body mass index between groups. The participants had a gender distribution of 24 females and 18 males (Table 1). Participants were recruited by flyers within the University of Cambridge, through invitation by the dermatology clinic of Cambridge University Hospital NHS Foundation Trust, or through invitation by the Alliance for Cancer Early Detection (ACED) cohort.

Participants were eligible if they were between the ages of 20 and 80, could provide informed written consent in English, and had a body mass index (BMI) of between 18.5 and 29 (not obese or underweight). Participants were excluded if they had tattoos or scarring over the imaging regions or if they had a pulmonary sleep disorder or any other active respiratory condition that could affect blood oxygenation. Throughout the procedure, participants were monitored for adverse events, such as unacceptable skin erythema, at which point they would have been withdrawn. No adverse events were observed. Participants were free to withdraw at any point.

Prior to the study visit, potential participants were screened to assess their eligibility and Fitzpatrick skin type assignment. The Fitzpatrick skin type was identified by a questionnaire to ensure that we recruited a maximum of 6 participants for each group. While the Fitzpatrick skin type was originally designed as a method for categorising a person’s risk of skin cancer^31^, it has since been used in several studies as a proxy measure of skin colour. It does not consider differences in skin colour between different positions of the body or changes over time (e.g., because of vitiligo or sun exposure) therefore it was used here solely as a straightforward method for ensuring that a representative, diverse cohort of participants were recruited.

Participants were invited for a single visit of approximately 1 hour duration. At the start of the visit, written informed consent was obtained, height and weight were measured, and eligibility was confirmed. The desired imaging sites were shaved if necessary to ensure good acoustic coupling between the photoacoustic probe and the skin. Colorimeter measurements (Colorimeter DSM-4, Cortex Technology, Aalborg, Denmark) were taken at each imaging site (over the carotid artery, the bicep muscle, the radial artery and the calf muscle), giving a quantitative measure of skin colour. Five repeat measurements were made, and the mean average was taken. A single outlier ITA measurement was excluded from the radial artery ITA measurements of participant 25, as it was 5 standard deviations away from the mean of the other samples. The colour measurement is based on diffuse reflectance spectroscopy in a 45°/0° configuration. The colorimeter returns the reflected colour in several colour space representations (CIE-L*a*b*, CIE-L*C*h, CIE-XYZ) and several derived quantities, including the individual typology angle (ITA, Eq. 1), which has been proposed as a quantitative measure of skin colour^40^. All specular reflections were excluded from the measurement. Measurements were performed within an 8 mm diameter opening in the front of the device. The colorimeter was calibrated daily, using dark, white and gloss references provided by the manufacturer.

1 $${{{\rm{ITA}}}}=\frac{180}{\pi }\arctan \left(\frac{{L}^{*}-50}{{b}^{*}}\right)$$

Pulse oximetry (B125 Patient Monitor, GE Health, Wisconsin, United States) blood oxygen saturation readings were recorded at the start of each scan. The pulse oximeter was used to give an approximation of the oxygen saturation of the arterial blood in the finger.

### Photoacoustic imaging

PAI was conducted using the MSOT Acuity Echo CE system (iThera Medical GmbH, Munich, Germany). Procedures were conducted under laser safety-approved conditions. Light excitation pulses were provided by a tuneable optical parametric oscillator (OPO) pumped by a neodymium-doped yttrium aluminium garnet (Nd-YAG) laser, with a pulse repetition rate of 25 Hz and pulse duration of 4–7 ns^41^. The laser output wavelength can be tuned from 660 nm to 1300 nm. Laser light was delivered to the probe by a single optical fibre. The output energy density of the laser was less than 20 mJ/cm^2^, falling below the maximum permissible exposure limits. The laser energy of each pulse was measured automatically by the system.

To ensure that the photoacoustic signal was consistent over time, a vendor-supplied phantom was measured daily prior to each participant visit. Measurements were taken over the full range of wavelengths in 10 nm steps. The phantom consisted of four absorbing inclusions. To assess reproducibility, regions of interest were drawn manually around each inclusion, the mean photoacoustic signal was calculated for each time point at each wavelength, and the coefficient of variation over time was calculated by dividing the standard deviation over time by the mean.

The photoacoustic signals were detected by a two-dimensional array of ultrasound transducers arranged in an arc shape within the probe (central detection frequency 4 MHz, 256 detector elements). The resulting images have a field of view of 30 mm and spatial resolution 150 µm. Reflection ultrasound computed tomography images are provided by the system in parallel, giving intrinsically co-registered anatomical information alongside the functional information provided by PAI. The ultrasound images were used to position the probe, as they provided much clearer anatomical information than photoacoustic images.

PAI was carried out on several sites in the body. Clear ultrasound gel (Parker Aquasonic Clear) was applied to the probe before imaging. In the non-vitiligo cohort, we scanned the lower leg (calf muscle), the forearm (transverse and longitudinal radial artery), the upper arm (bicep muscle) and the neck (transverse carotid artery). In the vitiligo cohort, we scanned the same positions, with additional scans taken of specific vitiligo-affected areas where a contralateral non-affected area could be identified. Each position measured first with the colorimeter and then scanned for 30 s at a time and marked with a small sticker when necessary to identify the scan location for subsequent measurements. Three successive 10 s scans were acquired, from which values were aggregated (mean) for subsequent analyses. The whole imaging procedure was repeated three times to assess the reproducibility of the resulting images. To minimise the effects of patient and probe motion, three frames with minimal motion were chosen from each 30 second scan for subsequent analysis.

### Image reconstruction and processing

Photoacoustic images were reconstructed and analysed using the PATATO toolkit and custom Python code^42^. Images were reconstructed using an iterative model-based image reconstruction algorithm (field of view 4 cm, 400 × 400 pixels). The time-series data was band-pass filtered before model-based reconstruction (5 kHz to 7 MHz). The filtered signals were divided by laser pulse energy to correct for per-pulse energy fluctuations. A qualitative depth-exponential weighting was applied to all images to improve the image contrast at depth for visualisation; however, this weighting was not used in the quantitative analysis.

Spectral unmixing was applied to the reconstructed data using the PATATO toolkit. The images were unmixed pixel-wise using literature values of the absorption spectra. Each pixel was expressed as a linear combination of deoxy-haemoglobin (Hb) and oxy-haemoglobin (HbO_2_) using NumPy’s matrix pseudo-inverse function. Total haemoglobin (THb = HbO_2_ + Hb) and blood oxygenation estimates (sO_2_^EST^ = HbO_2_ / THb) were obtained from the unmixed coefficients.

Reconstructed ultrasound images were provided by the imaging system and were acquired in sequence with the photoacoustic images. Ultrasound images from each scan were used to identify a single frame in each 10 s scan that had minimal motion between the first and last wavelength of the PAI frame. This frame was used in the subsequent analyses. Polygonal regions of interest were drawn around the skin surface (this consists of the epidermis but includes other tissue as we are limited by our imaging resolution, which is larger than a typical epidermis thickness), arteries, muscles and subcutaneous fat regions using ultrasound images as a reference. These were converted into binary masks and applied to the photoacoustic images. Unless specified, thresholds were applied to sO_2_^EST^ quantification when masks were applied; pixels with sO_2_^EST^ greater than 1 or smaller than 0 were ignored. Mean values of each metric (unmixed or single wavelength, as specified in the results) were calculated across the region of interest. Negative pixel values were excluded when calculating the mean spectra or total haemoglobin quantities unless otherwise specified.

The regions of interest analysed showed some variation in size and depth (mean ± standard deviation): radial artery diameter 8.5 ± 1.0 mm, depth 2.9 ± 0.8 mm; carotid artery diameter 10 ± 4 mm, depth 15 ± 3 mm. The centroid of the bicep muscle region was at a depth of 14.2 ± 1.4 mm, while that of the calf muscle was 14.3 ± 1.6 mm. Muscle regions of interest included as much of the muscle as was visible in the field of view. No statistically significant linear relationship was observed between ITA and bicep muscle depth (*p* = 0.14) or radial artery depth (*p* = 0.96).

Contrast to noise ratio (CNR) was calculated by evaluating the mean photoacoustic signal in the radial artery region of interest (${S}_{s}$), subtracting the mean photoacoustic signal in a background region (${S}_{b}$) constructed with the same area, 1 cm to the left, at the same depth, then dividing by the standard deviation of the signal in the background region ${\sigma }_{b}$^43^.

2 $${{{\rm{CNR}}}}=\frac{|{S}_{s}-{S}_{b}|}{{\sigma }_{b}}$$

### Optical modelling and fluence correction

We simulated light fluence in a tissue model using a Monte-Carlo simulation in Monte Carlo eXtreme (MCX)^44^. The model was defined on a 1000 × 1000 × 1000 grid with pixel size 60 µm. Scattering properties were defined using typical values seen in soft tissue throughout, ${\mu }_{s}^{{\prime} }=2.2{\left(\frac{\lambda }{500{\mbox{nm}}}\right)}^{-0.66}$ mm^−1^, *g* = 0.9^3^. Three layers were used in the tissue model. On top, a 60 µm epidermis was modelled, with melanosome volume fraction varying logarithmically from 0.02 to 0.4 in 6 steps. Below, a 1 mm-thick dermis layer was modelled, with 0.2 % blood volume fraction and 70 % oxygenation. The rest of the simulation volume comprised a background layer with 70 % blood oxygenation and 2.5 % blood volume fraction^11^. Finally, a blood vessel was added with radius 1.5 mm, 100 % blood oxygenation and 100 % blood volume fraction, at a depth of 5 mm.

The Monte-Carlo simulation was run with 10^7^ photons per simulation, at 41 wavelengths from 700 nm to 900 nm inclusive. To confirm the convergence of our simulations, we evaluated the difference in fluence in the simulated blood vessel obtained between 10^7^ and 10^8^ photons in our simulation with the highest melanosome concentration, observing only a 1 % difference. We used an illumination geometry which approximates the clinical PAI system used in this study. The system consists of a single optical fibre with a beam divergence of 8.66°, 43.2 mm above the surface of the probe, directed at an angle of 22.4° from the imaging plane, intersecting the imaging plane 2.8 mm outside of the probe. All simulations were run on a computer with 2 NVIDIA Quadro 8000 GPUs (48 GB RAM each), Intel Xeon Gold 6230 CPU at 2.10 GHz (40 cores), and 256 GB RAM.

The fluence across the blood vessel region was averaged and saved for each wavelength and melanosome volume fraction. A lookup table for correction was then constructed by normalising all the factors by the wavelength-dependent fluence for the lowest melanosome volume fraction (0.02). The individual typology angle (ITA) measurements from colorimeter were related to melanosome volume fraction of the simulation by a previously published adding-doubling diffuse reflectance simulation^11^. The previous study estimated ITA as a function of melanosome volume fraction in the same simulation conditions as used here for the Monte-Carlo model. A quadratic curve was fit to the points from the previous study and used to estimate the appropriate melanosome volume fraction from the experimental ITA value.

To correct experimental data, we interpolated the normalised fluence correction factor using a cubic spline interpolation across wavelength and melanosome volume fraction using the SciPy class RegularGridInterpolator in Python. Each entire experimental photoacoustic image was then divided by the factor for the corresponding wavelength, after which the standard analysis and linear unmixing approach was taken.

### Melanin absorption plane-wave ultrasound reconstruction

Melanin ultrasound (mUS) images were generated from the raw photoacoustic time series data by adapting a pulse-echo plane-wave ultrasound reconstruction algorithm^36^. At shorter optical wavelengths in subjects with darker skin, our photoacoustic time series acquisition resembles a plane-wave ultrasound imaging system like that described by Pham, K. et al.^36^ due to the strong, highly localised, and approximately planar optical absorption by melanin in the epidermis. Previous implementations of this algorithm used a planar ultrasound detector in the same position as the plane wave generation. In this study, however, an arc-shaped ultrasound transducer array was used, which was spatially offset from the skin surface, the source of the plane waves. The plane-wave reconstruction algorithm described by Cheng, J. & Lu, J.-y.^45^ was therefore adapted to account for these two differences.

#### Theoretical background

In two dimensions, the conventional plane-wave reconstruction algorithm aims to recover a B-mode-like ultrasound image, $a\left(x,y\right)$, which resembles the spatial distribution of ultrasound scatterers under a Born approximation. The method assumes that the acoustic field can be expressed as a linear sum of plane waves, and that the dispersion relation $\frac{{\omega }^{2}}{{c}^{2}}={k}_{x}^{2}+{k}_{y}^{2}$ applies^45^. Given the dispersion relation, any acoustic field can therefore be expressed as a function of any two of ${k}_{x}$, ${k}_{y}$ and ω. For an incident plane-wave ${p}_{{\mbox{plane}}}\left(x,y,t\right)=\delta \left(y-{ct}\right)$ and resultant acoustic pressure time series measured by some detectors at $\left({x}_{i},{y}_{i}\right)$, $p\left({x}_{i},{y}_{i},t\right)$, the algorithm proceeds by computing the Fourier transform of the pressure field, $P\left({k}_{x},{k}_{y}\right)$ and applying the mapping ${k}_{y}^{{\prime} }={k}_{y}+\omega /c$. The mapping gives the Fourier transform of the scattering distribution, $A\left({k}_{x},{k}_{y}^{{\prime} }\right)$. By applying the inverse Fourier transform, the scattering distribution can be recovered, $a\left(x,y\right)$.

With planar ultrasound detection (detectors along the line y=0), the algorithm proceeds by applying a two-dimensional Fourier transform to the measured time series $p\left(x,y=0,t\right)$ along the x and t axes, giving $P\left({k}_{x},\omega \right)$. Through the dispersion relation, this can be mapped to $P\left({k}_{x},{k}_{y}\right)$. Applying the second mapping, ${k}_{y}^{{\prime} }={k}_{y}+\omega /c$, to get $A\left({k}_{x},{k}_{y}^{{\prime} }\right)$, and inverse Fourier transforming, the scattering distribution, $a\left(x,y\right)$ can be recovered. To account for a spatial offset between the skin surface (source of incident plane waves) and the ultrasound transducers, $P\left({k}_{x},{k}_{y}\right)$ is multiplied by a phase factor $\exp \left(i{k}_{y}{\delta }_{y}\right)$ before mapping to ${k}_{y}^{{\prime} }$ coordinates and $A\left({k}_{x},{k}_{y}^{{\prime} }\right)$ is multiplied by the phase factor $\exp \left(-i{k}_{y}^{{\prime} }{\delta }_{y}\right)$ prior to the final inverse Fourier transform, where ${\delta }_{y}$ is the perpendicular distance between the skin surface and the transducer array.

For the circular arc-shaped system used in this study, a plane wave ultrasound reconstruction algorithm was developed by analogy with the linear array. The Fourier transform of the pressure field, $P\left({k}_{x},{k}_{y}\right)$ was computed, as described elsewhere^46,47^. The algorithm then proceeds as above: multiply $P\left({k}_{x},{k}_{y}\right)$ by $\exp \left(i{k}_{y}{\delta }_{y}\right)$, apply the mapping ${k}_{y}^{{\prime} }={k}_{y}+\omega /c$ to get $A\left({k}_{x},{k}_{y}^{{\prime} }\right)$, multiply by $\exp \left(-i{k}_{y}^{{\prime} }{\delta }_{y}\right)$ and apply and inverse Fourier transform to give the ultrasound scattering distribution $a\left(x,y\right)$.

#### Implementation

The melanin absorption ultrasound reconstruction algorithm was implemented using custom-written code in Python and is publicly available https://github.com/BohndiekLab/PAISKINTONE. As with photoacoustic image reconstruction, a Butterworth band-pass filter was applied to the raw photoacoustic time series data between 5 kHz and 7 MHz using the SciPy functions butter (order 5) and filtfilt (even padding with length 100).

To isolate the scattered ultrasound waves, the non-scattered wave generated by the skin surface was subtracted from the filtered raw time series data. To calculate the non-scattered wave, the forward photoacoustic model was applied to the photoacoustic image with pixels outside the skin surface set to zero. The subtracted and filtered time series data was then used as input for the plane wave reconstruction algorithm described above.

To compute the Cartesian Fourier transform, $P\left({k}_{x},{k}_{y}\right)$ from the (filtered, subtracted) arc-shaped plane-wave time series data, $p\left(\theta,t\right)$, a Fourier-based method was used. First, a smooth crop of the time-series data was made. Then, the time series was interpolated onto a grid covering all 2π radians, with zeros used in place of missing detector angles. A two-dimensional Fourier transform was applied along the angle and time dimensions. A Hankel function weighting was applied, the data Fourier transformed again along the angle dimension, and interpolated onto a Cartesian grid to give $P\left({k}_{x},{k}_{y}\right)$. The plane-wave ultrasound reconstruction algorithm then proceeded as described above, implemented using the Python packages NumPy and SciPy.

#### Validation using acoustic simulations

To validate the adapted algorithm described above, two-dimensional k-space plane-wave ultrasound simulations were run in j-Wave, modelling the key components of ultrasound scattering reconstruction based on melanin absorption^48^. A simulation grid was defined with a width and height of 256 pixels; the pixel width was 50 µm. The background speed of sound was 1540 m s^−1^, and the background density was 1000 kg m^−3^. The initial pressure distribution was defined to mimic skin surface absorption, with a single pixel-width horizontal structure of constant value. A sensor array with 256-point detectors was defined parallel to the skin surface, with a defined spatial offset ${\delta }_{y}$. The initial pressure in a simulated blood vessel region was defined, either with the same amplitude (low melanin limit) or 100 times less amplitude (high melanin limit). Simulations were run in two dimensions, with a 512 × 512 grid with 200 µm spacing. To simulate ultrasound scattering structures, inhomogeneities in the tissue density and tissue speed of sound were introduced. Nine circular scattering structures were defined with radius 1.5 mm, 5 mm below the skin surface, evenly distributed on a square of side length 22.5 mm (Supplementary Fig. S12). Outside of the structures, the background density and speed of sound were multiplied by random noise sampled from a normal distribution with mean 1 and standard deviation 0.008. Inside the scattering circle structures, the density was sampled from a normal distribution with mean 1043 kg m^−3^ and standard deviation 61 kg m^−3^, and the speed of sound was sampled from a normal distribution with mean 1565 m s^−1^ and standard deviation 75 m s^−1^.

### Statistical analysis

Statistical analysis was carried out using the statsmodels library in Python. The relationship between continuous variables was assessed using a linear model, unless otherwise stated. Where a statistical test was applied to determine a dependence on skin pigmentation, the individual typology angle (ITA) was used as the dependent variable, and quoted p-values are the 2-tailed test. Where a linear model was not appropriate, a Spearman’s rank correlation test was applied using the spearmanr function in SciPy. T-tests were applied using the SciPy function ttest_1samp. Additional linear regression plots and box plots were made with Seaborn and matplotlib; in box plots, the median is represented by a solid black line, boxes show the upper and lower quartiles, whiskers show upper and lower quartiles plus or minus 1.5 times the inter-quartile range, respectively and the points outside this range are denoted by a black circle.

The theoretical model of sO_2_^EST^ dependence on ITA was compared to an alternative model (sO_2_^EST ^= constant) by calculating the likelihood ratio, using the Akaike information criterion (AIC) for the two models. The log likelihood was calculated using the residual values, ${r}_{i}$ as follows

3 $${{{{\rm{AIC}}}}}=2+{{{\rm{N}}}}\,\log \left(2\pi \right)+{{{\rm{N}}}}\,\log ({\sigma }^{2})+{{{\rm{N}}}}$$

4 $${\sigma }^{2}=\frac{{\sum }_{i}^{N}{r}_{i}^{2}}{N}$$

The likelihood ratio was then computed as follows:

5 $$\exp \left(\frac{{{{\rm{AIC}}}}_{1}-{{{\rm{AIC}}}}_{2}}{2}\right)$$

Where linear trend lines were not appropriate, locally weighted scatter plot smoothing (LOWESS) curves are included to guide the eye and highlight non-linear trends. LOWESS fits a smooth local polynomial regression line, allowing model fitting without the specification of a function. LOWESS was computed using the statsmodels lowess function. 75 % of the data was used when calculating each y-value (frac = 0.75 in the lowess function), and 3 residual-based re-weightings were performed (it = 3 in the lowess function). 95 % confidence intervals were calculated by bootstrapping, with 500 repeated re-samplings with replacement.

### Reporting summary

Further information on research design is available in the Nature Portfolio Reporting Summary linked to this article.

## Supplementary information

Supplementary Information

Reporting Summary

Transparent Peer Review file

## Data Availability

All raw data sets, including raw photoacoustic imaging data, pulse oximetry measurements, and colorimetry are available on Zenodo (10.5281/zenodo.20068219)^49^.

## Code availability

The code required to reproduce all analyses and figures in this manuscript is available at https://github.com/BohndiekLab/PAISKINTONE and is archived in this paper’s associated Zenodo dataset repository^49^.
