# Supplementary material for: The confounding effects of skin colour in photoacoustic imaging
Source: Nat Commun. 2026 Jun 17;17:7672. doi: 10.1038/s41467-026-74380-7 (PMC13433769; doi:10.1038/s41467-026-74380-7)
Supplement: Supplementary file 1 [file 41467_2026_74380_MOESM1_ESM.pdf]

Supplementary information for “The confounding effects of skin colour in photoacoustic imaging”

Thomas R. Else[1, 2, †], Christine Loreno[3], Alice Groves[3], Benjamin T. Cox[4], Evangelia Vetsiou[5], Janek Gröhl[1, 2, ‡], Inés Modolell[3, 5], Sarah E. Bohndiek[1, 2, *], Amit Roshan[1, 5, 6, *]

[1]Cancer Research UK Cambridge Institute, University of Cambridge; Cambridge, United Kingdom
[2]Department of Physics, University of Cambridge; Cambridge, United Kingdom
[3]ACED Clinic Cambridge, Early Cancer Institute, University of Cambridge; Cambridge, United Kingdom
[4]Department of Medical Physics and Biomedical Engineering, University College London; London, United Kingdom
[5]Cambridge University Hospitals NHS Foundation Trust; Cambridge, United Kingdom
[6]Barts Cancer Institute, John Vane Science Centre, Charterhouse Square; London, United Kingdom
[†]Present address: Department of Bioengineering, Imperial College London; London, United Kingdom
[‡]Present address: Institute for Experimental Molecular Imaging, RWTH Aachen University Clinic, Aachen, Germany
[*]Corresponding authors: SEB (*seb53@cam.ac.uk*), AR (*a.roshan@qmul.ac.uk*)

Supplementary tables

Table S1: ***Summary of all participants from the main cohort recruited to the study.*** *Note SKIN27 was consented but not recruited to the study due to equipment failure*

Study ID	Age range	Sex	Fitzpatrick type	BMI (kg m^{-2})	Neck ITA (°)	Leg ITA (°)	Forearm ITA (°)	Bicep ITA (°)
SKIN01	20-29	Male	2	25.5	54.2	61.7	64.5	70.4
SKIN02	30-39	Male	2	29.9	38.5	54.9	47.1	56.3
SKIN03	40-49	Male	3	27.5	17.1	31.6	34.7	49.4
SKIN04	20-29	Female	3	22	30.6	39	33.9	41.1
SKIN05	20-29	Female	3	21.5	36.3	43.3	48.4	51.3
SKIN06	70-79	Female	2	21.6	26.9	43.8	25.8	52.4
SKIN07	30-39	Male	3	27.7	33.5	46.2	33.2	42.7
SKIN08	30-39	Male	4	24.6	-20.2	-17.3	-12.8	-15.5
SKIN09	50-59	Female	4	21.9	7.7	15.4	0.4	9.4
SKIN10	60-69	Female	2	26.6	38.1	53.9	52.7	42.7
SKIN11	50-59	Female	2	18.5	22	21.3	32.4	14.7
SKIN12	70-79	Female	1	19.1	42.1	61.2	64.1	68
SKIN13	20-29	Male	2	25.2	40	20.6	47.6	40.1
SKIN14	30-39	Female	4	27.2	1.1	-8.6	1.5	3.8
SKIN15	30-39	Female	4	24.2	23.5	33.4	16.2	32.2
SKIN16	70-79	Female	1	20.2	39.1	40.1	47.8	50.3
SKIN17	20-29	Female	3	19.7	30.2	16.8	29.9	17
SKIN18	30-39	Female	3	23.2	25.4	16.6	25	20.6
SKIN19	30-39	Female	5	27.5	-59.2	-51.7	-9.4	-31.8
SKIN20	50-59	Male	1	23.1	42.5	62.1	56	61.2
SKIN21	20-29	Female	1	22	46.2	43.3	48.5	50.6
SKIN22	20-29	Male	4	22.6	-5.3	-29.5	-13.5	12.5
SKIN23	20-29	Female	1	21.9	52	61.9	56.3	54.6
SKIN24	50-59	Female	1	20.8	45.6	51.5	52.6	50.8
SKIN25	20-29	Male	4	19.6	6.2	23.7	22.1	25.4
SKIN26	60-69	Male	5	24.5	-7	7.1	-0.5	24.3
SKIN29	40-49	Male	6	22.5	-34	-34.8	-18.7	-26.7
SKIN31	30-39	Male	6	20.9	-53.4	-58.7	-25.4	-29.5
SKIN32	40-49	Male	5	25.1	0.1	10.7	2.9	-1.3
SKIN35	40-49	Female	6	26.4	-40.9	-51.9	-35.5	-39.7
SKIN38	20-29	Female	6	27.5	-67.3	-66.9	-42.1	-63.6
SKIN39	20-29	Male	5	24.5	-15	-46.3	-39.1	-21.7
SKIN40	20-29	Male	6	25.8	-59.2	-50.4	-42.1	-27.9

SKIN41	20-29	Male	6	19.6	-61.6	-55.5	-35	-29.9
SKIN42	20-29	Female	5	20.7	-17	-19	-19.2	-13.8
SKIN43	30-39	Female	5	20.2	-24.8	-9.1	-3.5	-13.7

Table S2: ***Summary of the participants with vitiligo included in this study.*** *Where participants had patches of vitiligo in a particular region, two ITA values are shown. The highest value corresponds to the ITA in the non-pigmented patch, and the lowest value corresponds to ITA in the pigmented patch.*

Study ID	Age range	Sex	BMI (kg m^{-2})	Neck ITA (°)	Leg ITA (°)	Forearm ITA (°)	Bicep ITA (°)
SKIN28	70-79	Female	28.7	44.9	53.3	66.5, 43.0	56.2
SKIN30	40-49	Female	20.3	51.2	54.6	57.1, 56.5	65.9, 55.2
SKIN33	40-49	Male	28.5	47.6	63.2	65.0, 50.3	68.3
SKIN34	60-69	Female	28.3	39.2	49.6	68.8, 42.1	37.5
SKIN36	70-79	Male	20.2	26.9, 1.1	44.9	49.8, 48.3	51.3
SKIN37	60-69	Female	23.9	37.5	n.a.	55.5, 33.8	16.6

Supplementary figures

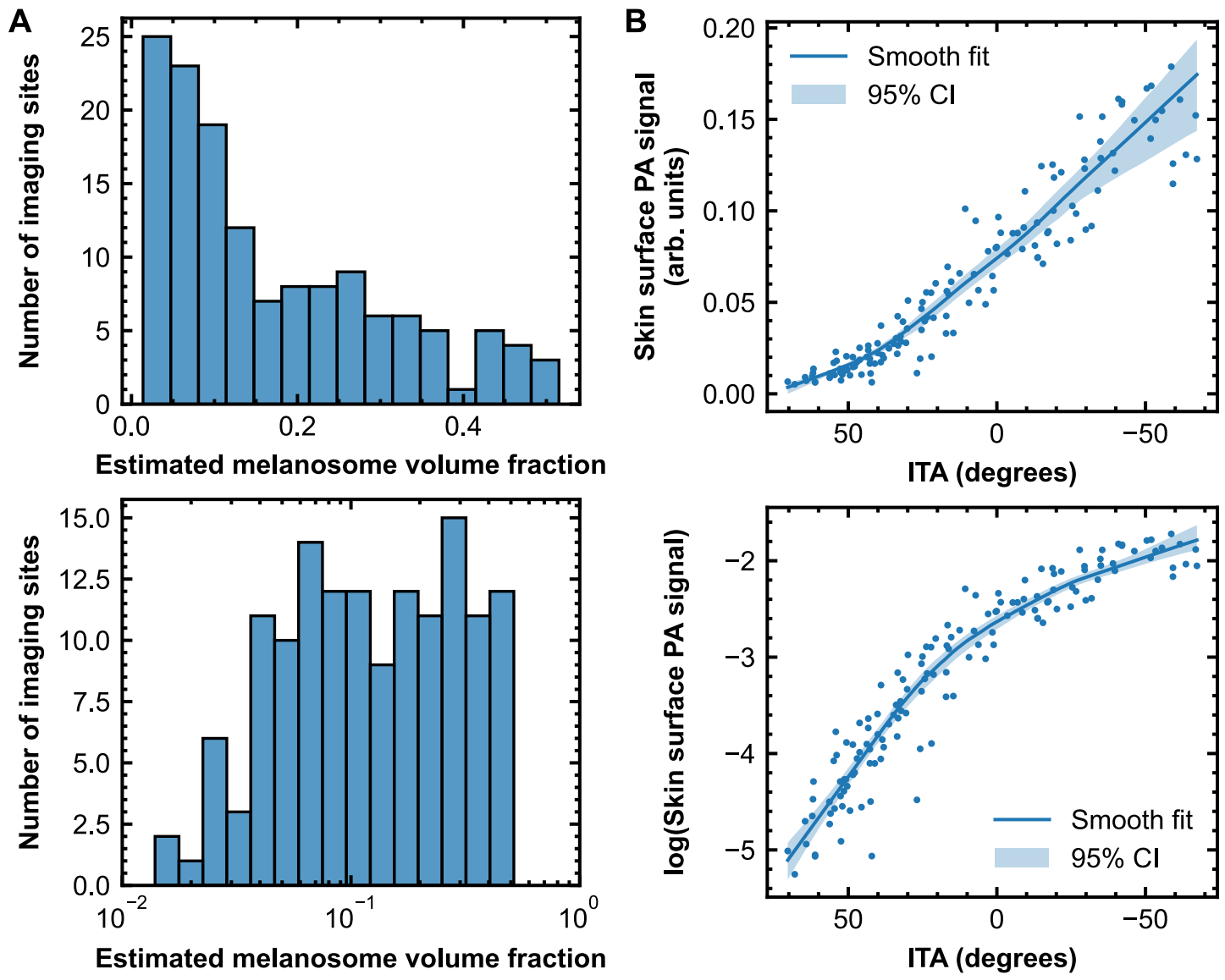

Fig. S1: ***The melanosome volume fraction distribution for all imaging sites is approximately uniformly distributed in log space between 0.04 and 0.4. Uncertainty in the fit of individual typology angle is a constant fraction of the photoacoustic signal. (A)*** *Histogram of the melanosome volume fraction, estimated from individual typology angle (ITA), on a linear scale and a logarithmic scale.* ***(B)*** *The skin surface photoacoustic (PA) signal as a function of ITA shown on a linear scale and a logarithmic scale. The smooth line in subpanel B is LOWESS fit, with the 95% confidence interval denoted by the shaded area. Each point represents the mean measurement for one imaging site for one participant.*

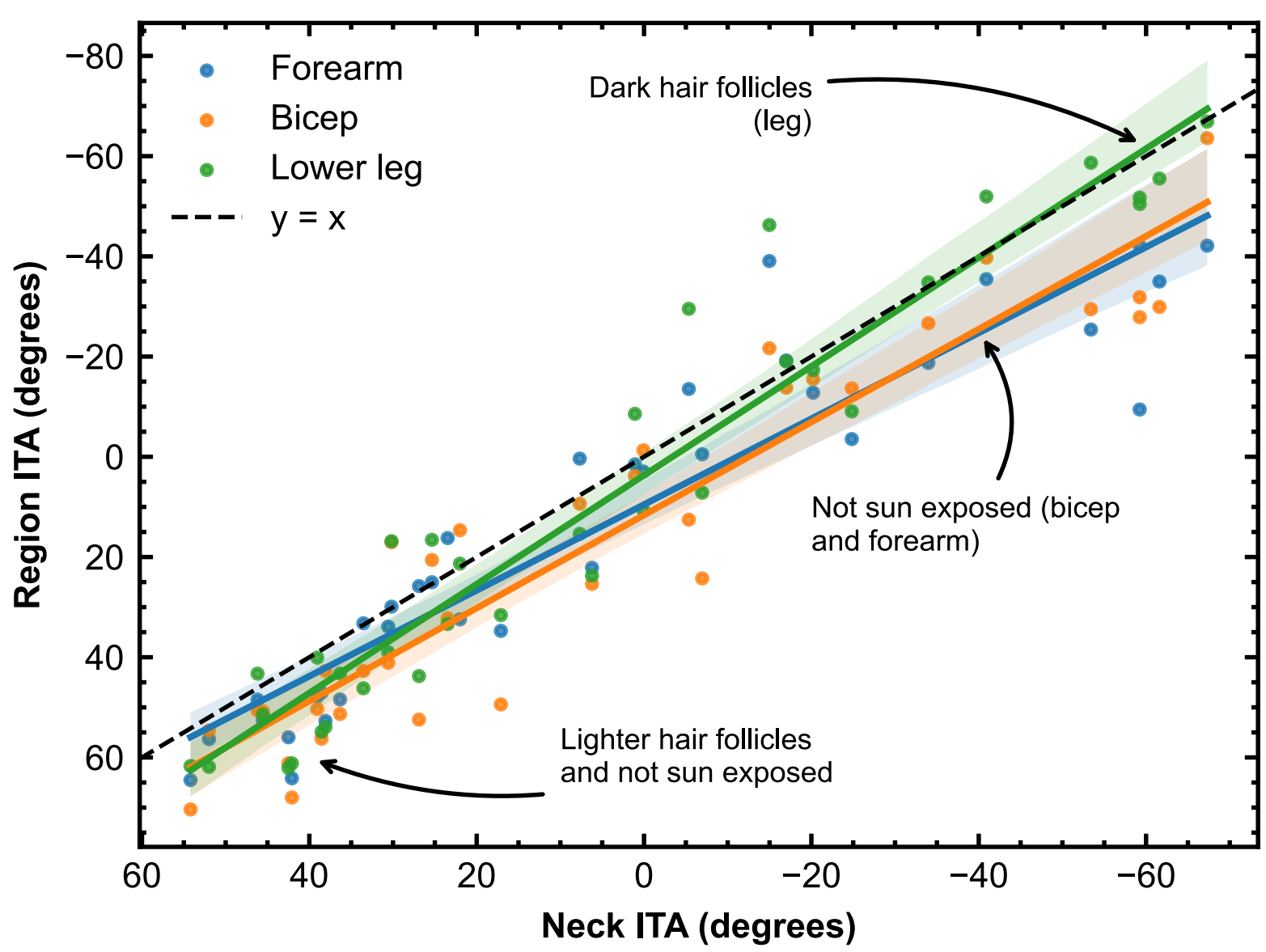

Fig. S2: ***Correlation between individual typology angle (ITA) measured in a sun-exposed area (neck) with forearm, bicep and lower leg regions.*** *Dotted line shows the line that would be predicted if ITA were equal across all regions. Shaded area shows 95% confidence interval of linear model. Solid line shows the mean linear line of best fit. Each point represents the mean measurement from a single person of the corresponding anatomical position.*

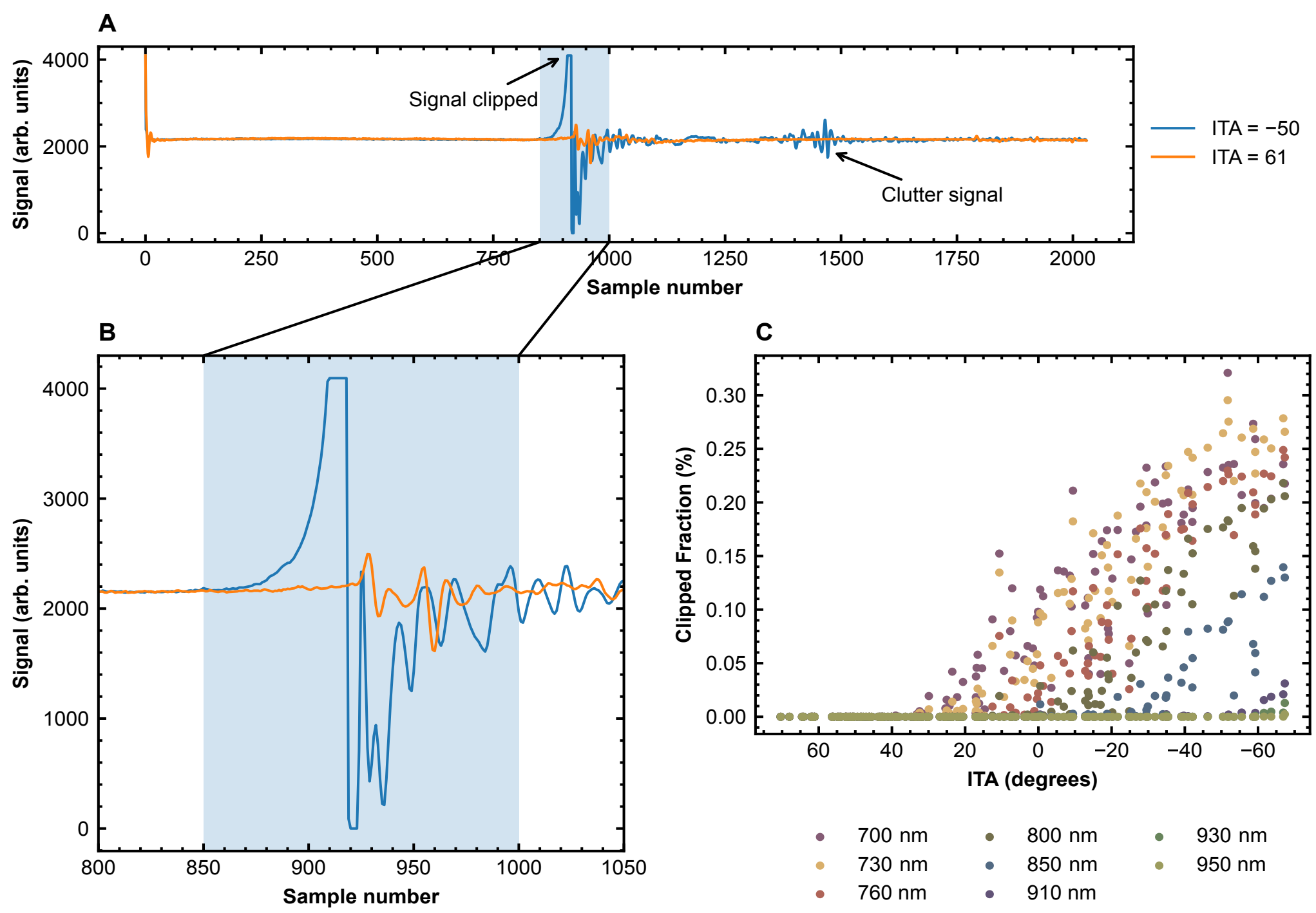

Fig. S3: ***Raw photoacoustic time-series data shows clipping in signal digitisation.*** *(**A** and **B**) Comparison of the raw time series data from a Fitzpatrick V subject, ITA = −50 (blue) and a Fitzpatrick II subject, ITA = 61 (orange). The signal can be seen to reach the limit of digitisation during the arrival of the skin signal.* ***(C)*** *The fraction of time samples that were clipped is shown as a function of wavelength and skin type. Each point represents the mean measurement for one imaging site for one participant.*

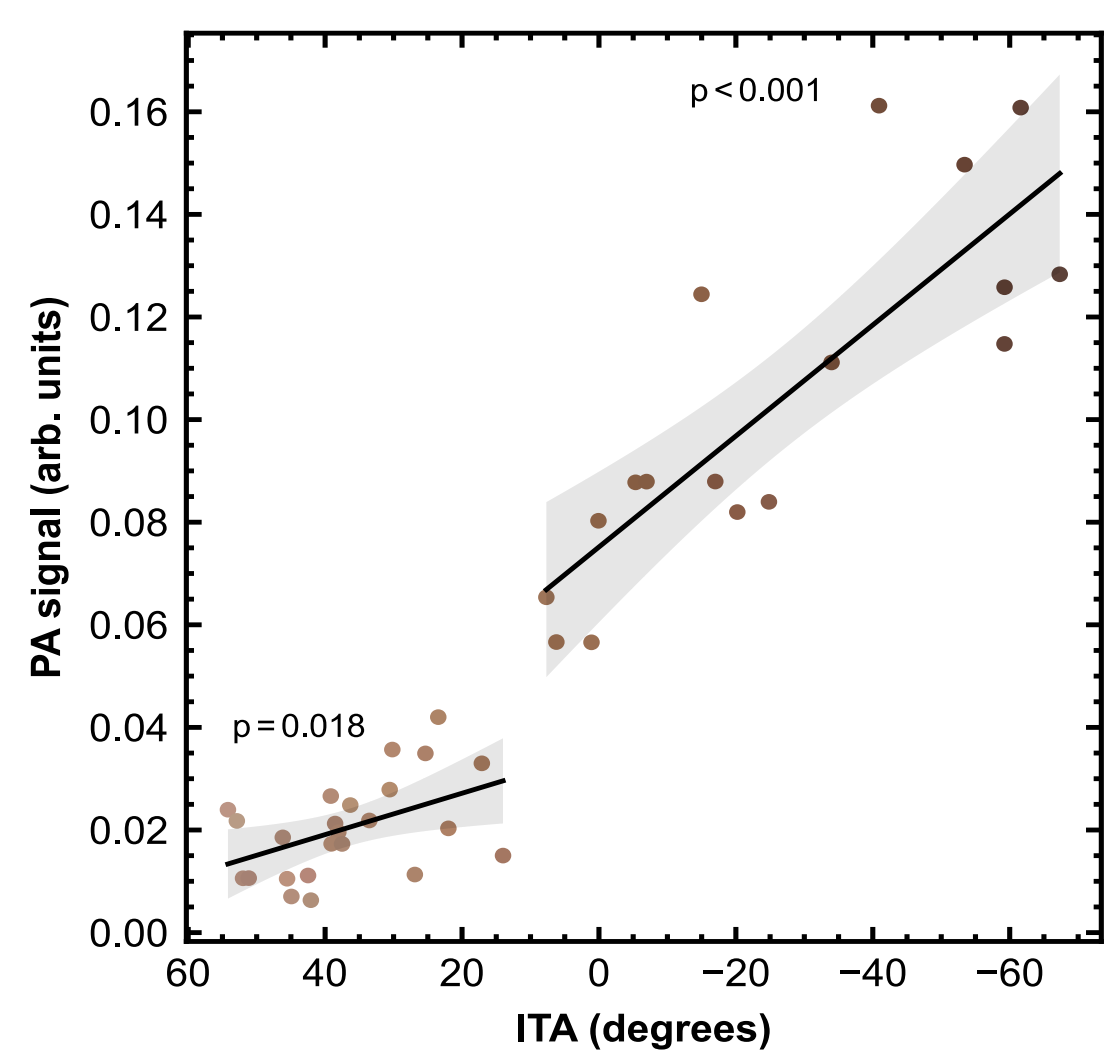

Fig. S4: ***Fit of photoacoustic signal in the skin surface region of the neck as a function of individual typology angle (ITA) for low (< 10) and high (>10) ITA values.*** *Solid lines show a linear regression line; shaded area shows 95% confidence intervals. P-values are taken from a separate linear model for each group (higher ITA: p=0.018, n=24 subjects, r^2=0.19; lower ITA: p=4x10^{-5}, n=17 subjects, r^2=0.67).*

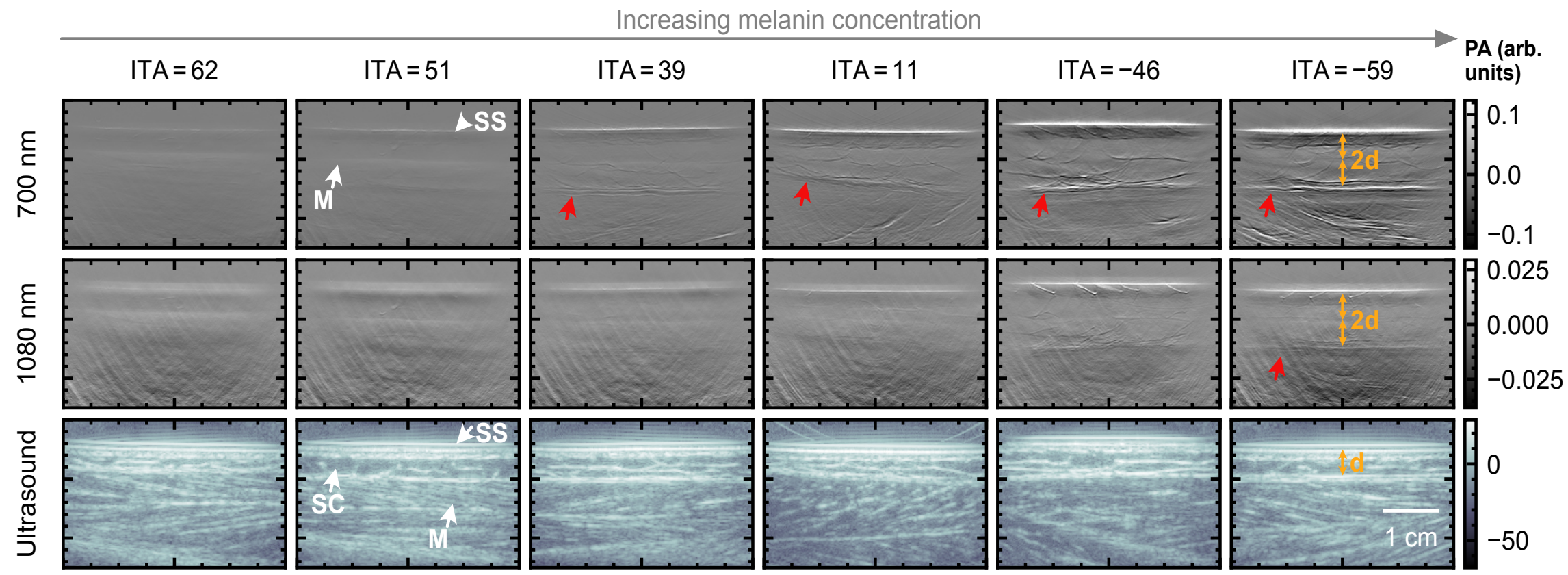

Fig. S5: ***Example photoacoustic and ultrasound images of the calf muscle at representative skin tones and several wavelengths.*** *Key anatomical features are highlighted by a white arrow, and acoustic reflection artefacts are highlighted by a red arrow (M = muscle, SS = skin surface, SC = subcutaneous fat). Orange arrows show that the artefact depth is twice the depth from the skin surface to muscle-fat interface. A qualitative exponential signal intensity depth compensation is applied to enhance the visibility of deeper structures.*

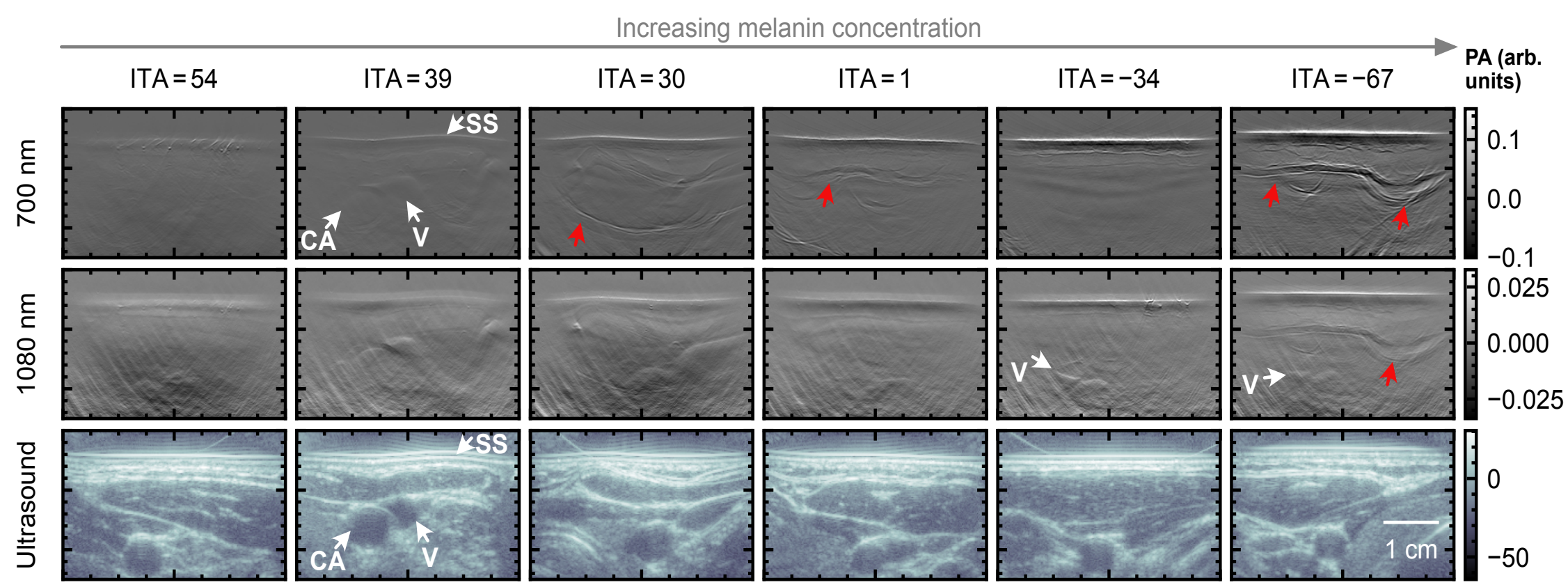

Fig. S6: ***Example photoacoustic and ultrasound images of the carotid artery at representative skin tones and several wavelengths.*** *Key anatomical features are highlighted by a white arrow and acoustic reflection artefacts are highlighted by a red arrow (V = vein, CA = carotid artery, SS = skin surface). A qualitative exponential signal intensity depth compensation is applied to enhance the visibility of deeper structures.*

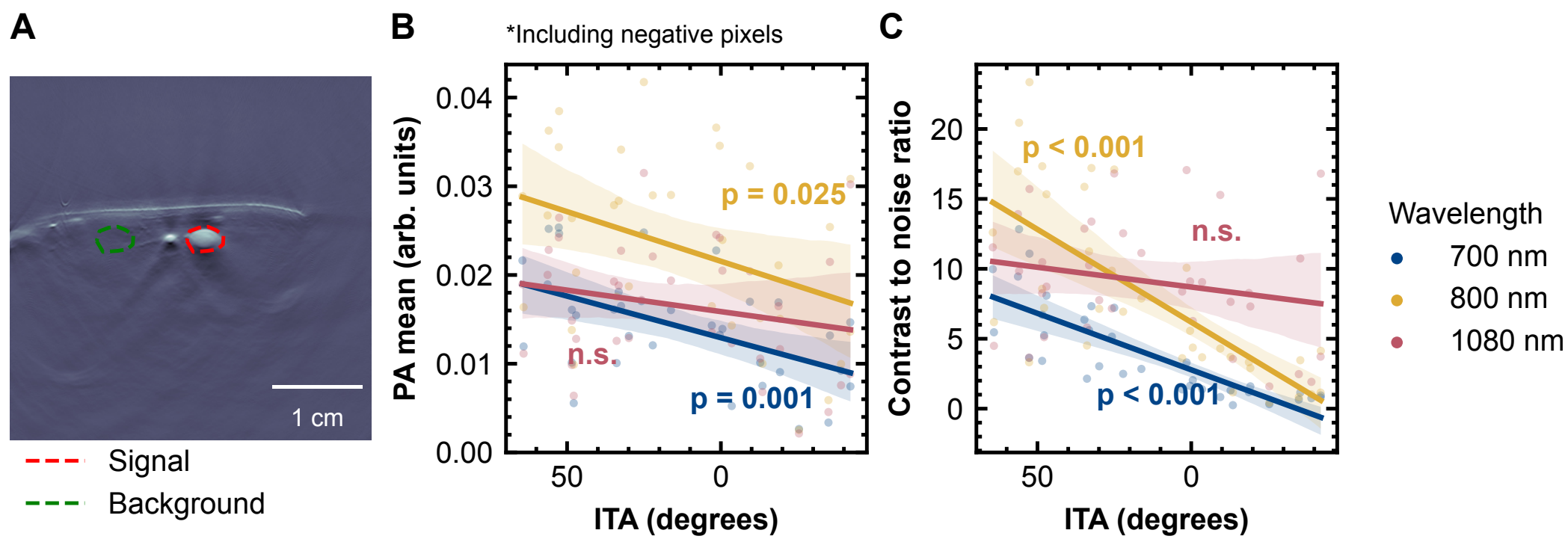

Fig. S7: ***Quantification of signal intensity in the radial artery including negative pixels and contrast to noise ratio. (A)*** *An example photoacoustic image with the regions of interest used to calculate the mean signal and mean background signal.* ***(B)*** *Mean photoacoustic signal at three representative wavelengths in the radial artery including negative pixels as a function of individual typology angle (ITA). Linear model statistical test on gradient (700 nm: p=0.001, n=35 participants, r^2=0.25; 800 nm: p=0.03, n=35 participants, r^2=0.12; 1080 nm: p=0.2, n=35 participants, r^2=0.02).* ***(C)*** *Contrast to noise ratio between the signal and background regions as a function of ITA (700 nm: p=6x10^{-9}, n=35 participants, r^2=0.64; 800 nm: p=1x10^{-6}, n=35 participants, r^2=0.50; 1080 nm: p=0.2, n=35 participants, r^2=0.05). P-values are calculated for two-tailed tests on the gradient of a linear model. Solid line shows the mean of a linear regression model; the shaded area shows the 95% confidence interval.*

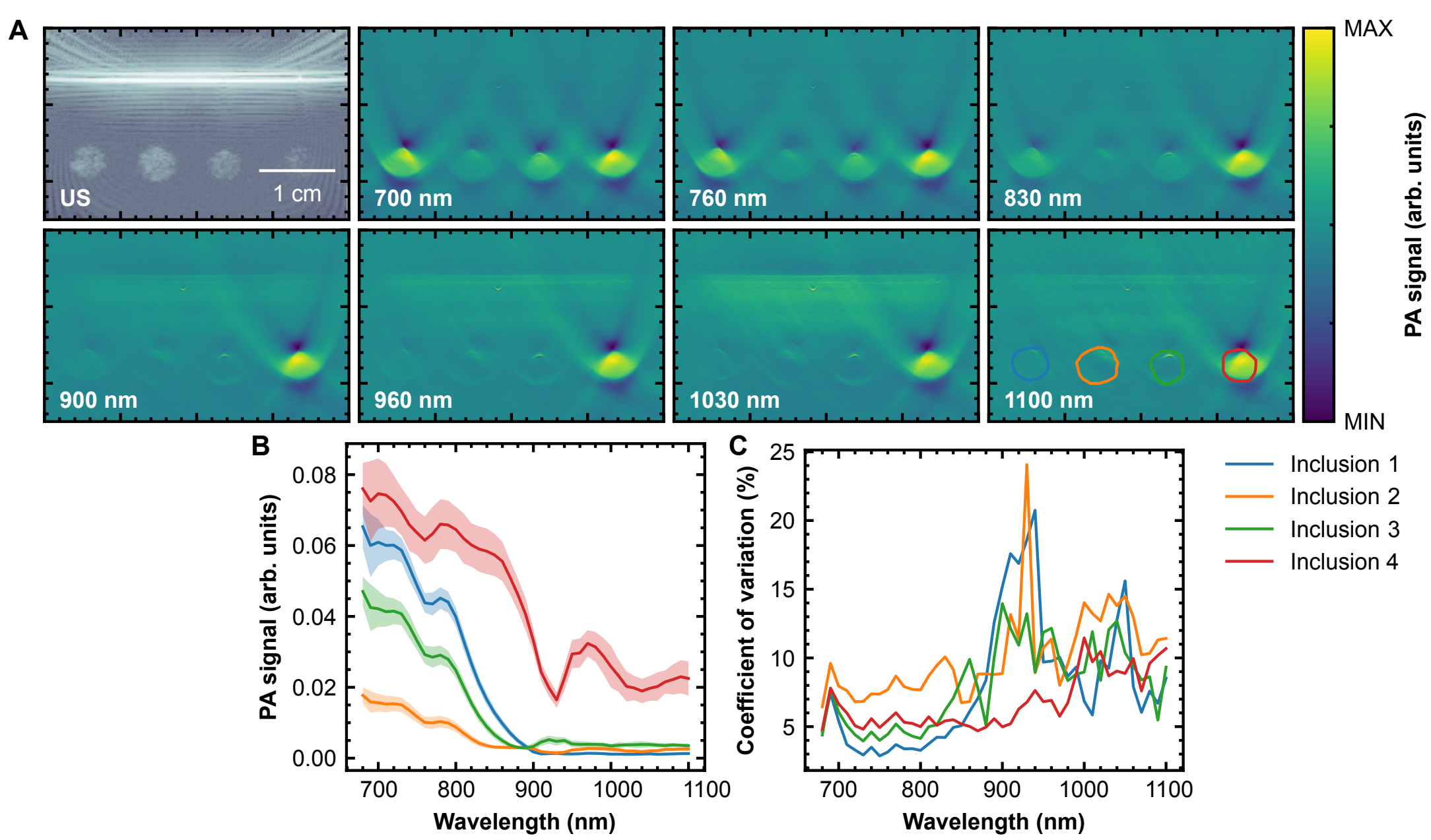

Fig. S8: ***Characterisation of phantom imaging reproducibility over the course of the study. (A)*** *Example ultrasound image of the test phantom.* ***(B)*** *Mean photoacoustic spectrum across (solid line) and standard deviation (shaded area) in each phantom inclusion, across phantom scans.* ***(C)*** *Coefficient of variation across all wavelengths for each phantom inclusion.*

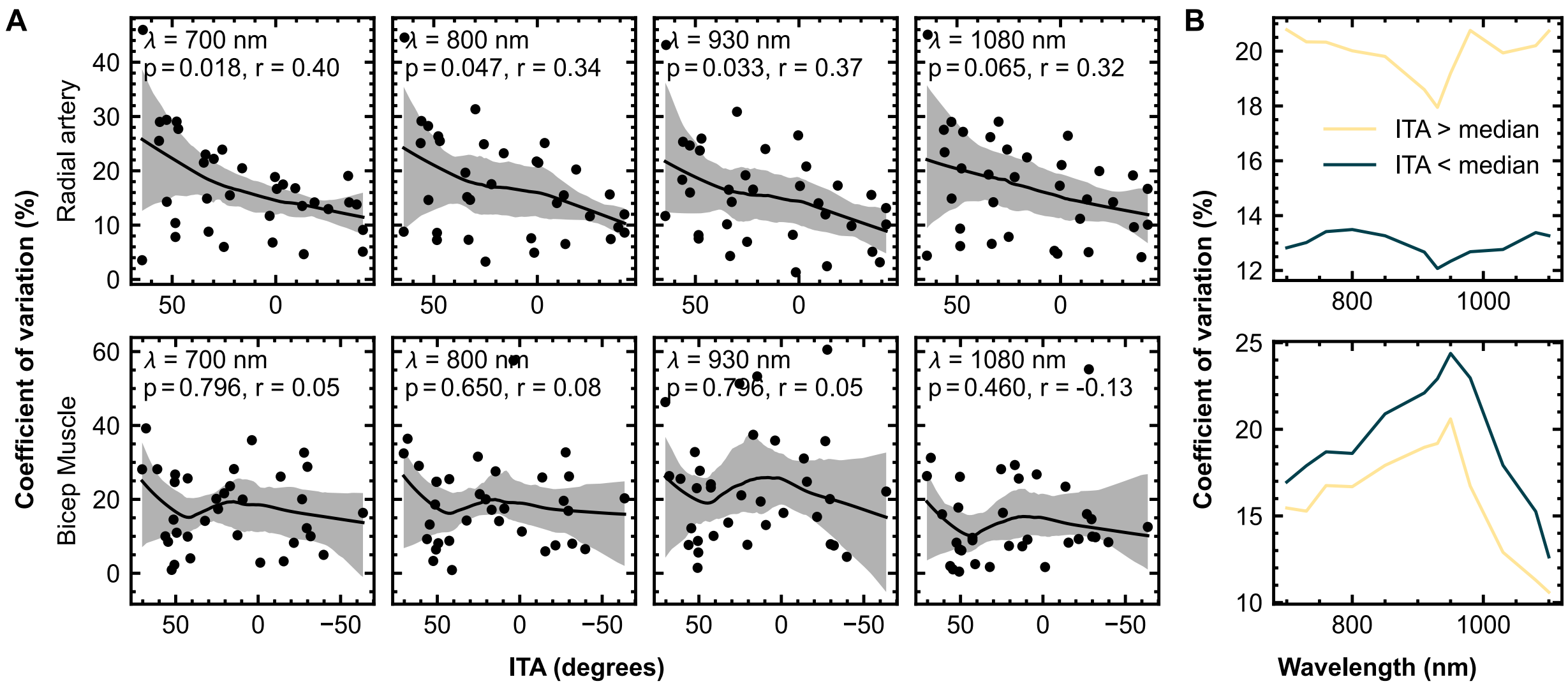

Fig. S9: ***Coefficient of variation in single wavelength measurements averaged across the radial artery and bicep muscle. (A)*** *A plot of coefficient of variation as a function of individual typology angle (ITA) at several wavelengths for each region of interest, with a LOWESS smoothing curve and 95% confidence intervals shown as the shaded region, calculated by bootstrapping. Spearman's rank correlation coefficient r and p values are shown (n=35 participants).* ***(B)*** *The mean coefficient of variation across all wavelengths is plotted for the half cohort of participants with lighter skin (ITA > median) and darker skin (ITA < median) in the radial artery and bicep muscle.*

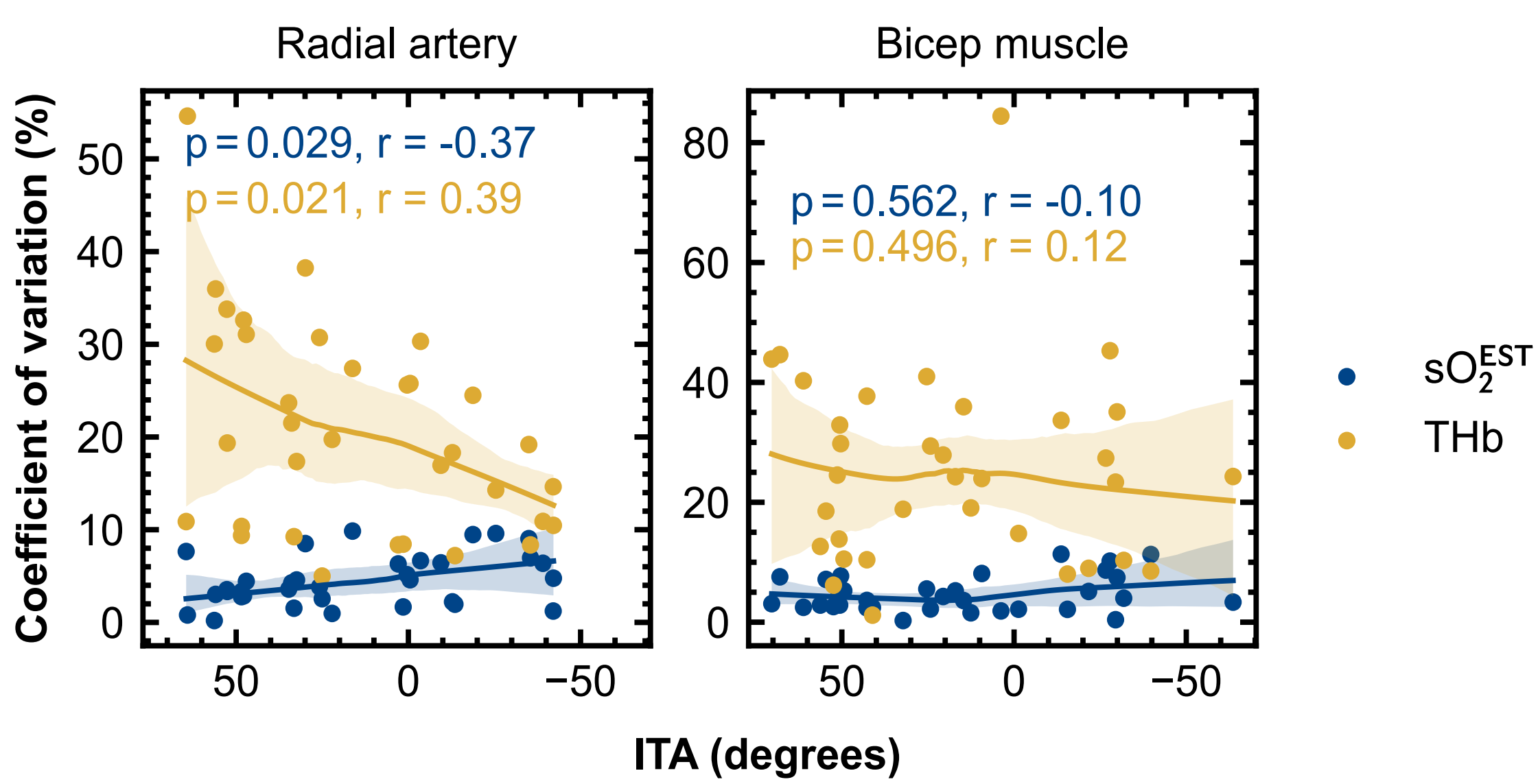

Fig. S10: ***Coefficient of variation in linear unmixing quantities sO2EST and THb, averaged across the radial artery and bicep muscle.*** *A plot of coefficient of variation as a function of individual typology angle (ITA) for each region of interest. Spearman's rank correlation coefficient r and p values are shown (n=35 participants). LOWESS smoothing is shown with 95% confidence intervals shaded.*

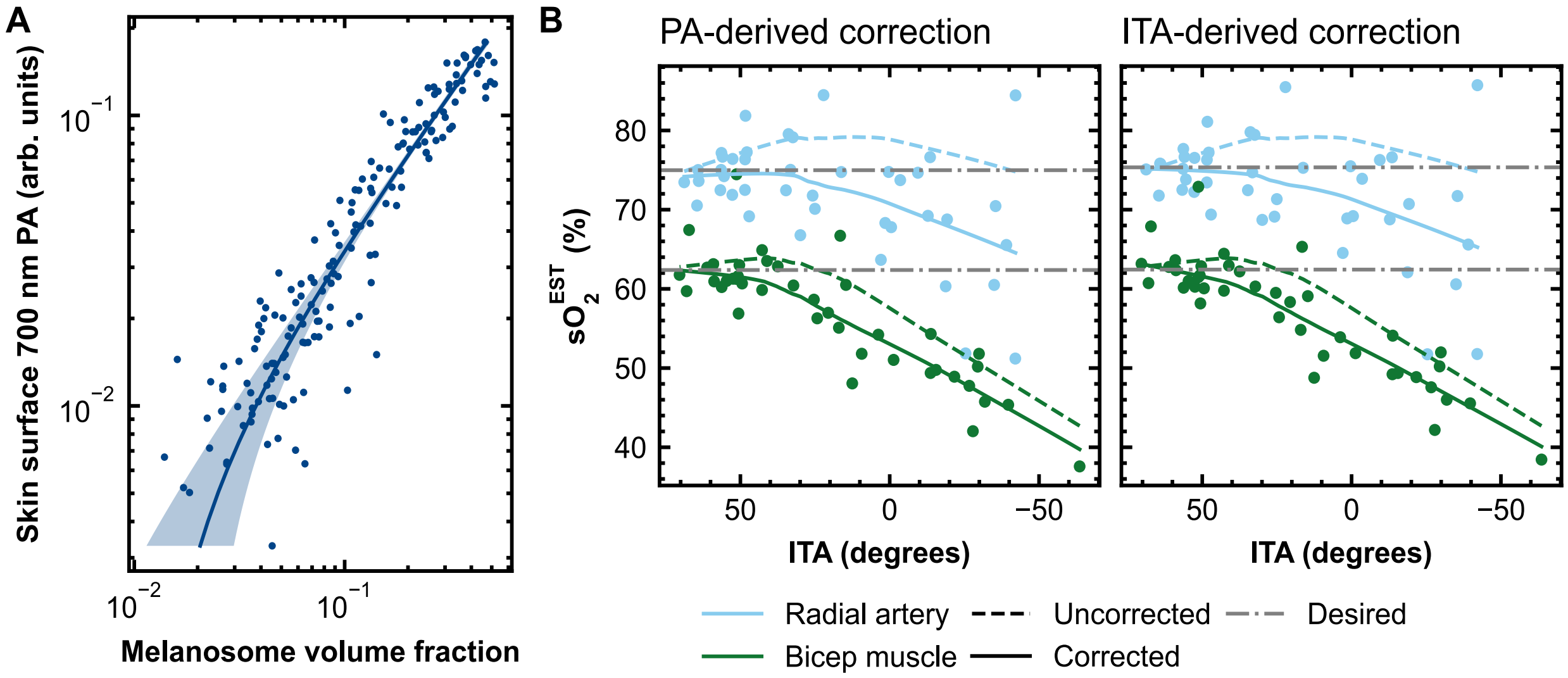

Fig. S11: ***Spectral colouring correction can be applied using photoacoustic signal in the skin surface as a measure of melanosome volume fraction instead of individual typology angle (ITA). (A)*** *A linear relationship was derived between melanosome volume fraction (derived from colourimeter ITA values) and the mean positive 700 nm photoacoustic (PA) signal in the skin surface (each point represents a single imaging site from a single participant, the solid line shows the mean linear regression line, and the shaded area shows the 95% confidence interval).* ***(B)*** *Comparison of the sO_2^{EST} in different regions of interest derived from multispectral PA signals after correction based on a fluence correction factor derived directly from the photoacoustic signal (left) or from the external colorimeter measurement of ITA (right). The points and solid line show results after correction, while the dashed line shows the uncorrected relationship. The grey line shows an idealised case where there is no dependence between sO_2^{EST} and ITA.*

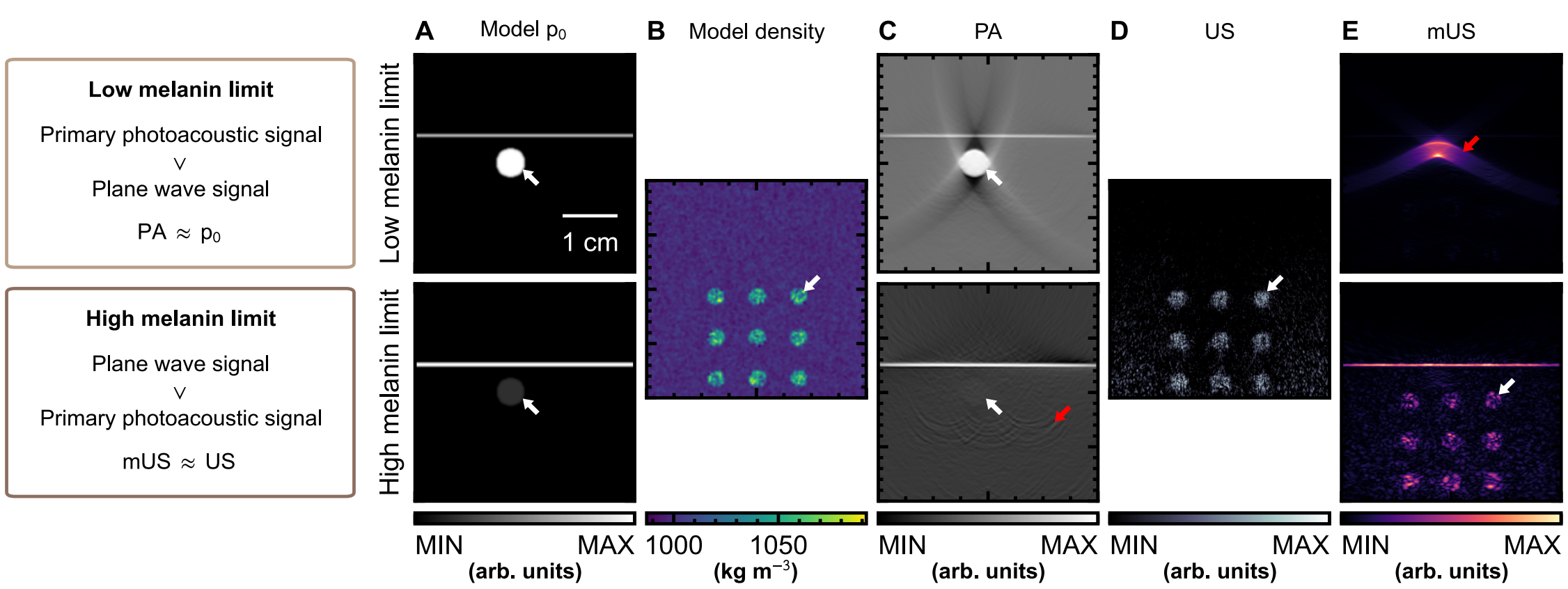

Fig. S12: ***Acoustic simulations in the low and high melanin concentration limits validate the melanin absorption ultrasound (mUS) reconstruction method.*** *Initial pressure* ***(A)*** *and tissue density* ***(B)*** *parameters used in the acoustic simulations; bright circular structures represent a blood vessel (p_0) and generic ultrasound scatterers (density) respectively.* ***(C)*** *Reconstructed simulated photoacoustic (PA) images show the skin surface, the blood vessel (white arrow) and artefacts (red arrows).* ***(D)*** *Reconstructed simulated ultrasound (US) images.* ***(E)*** *Reconstructed melanin ultrasound images based on plane wave reconstruction. Artefacts in each image are highlighted with a red arrow. The blood vessel is indicated with a white arrow in the PA image and ultrasound scatterers are highlighted with a white arrow in US and mUS images. Note: the colour scales have been manually adjusted to highlight key artefacts and image features.*

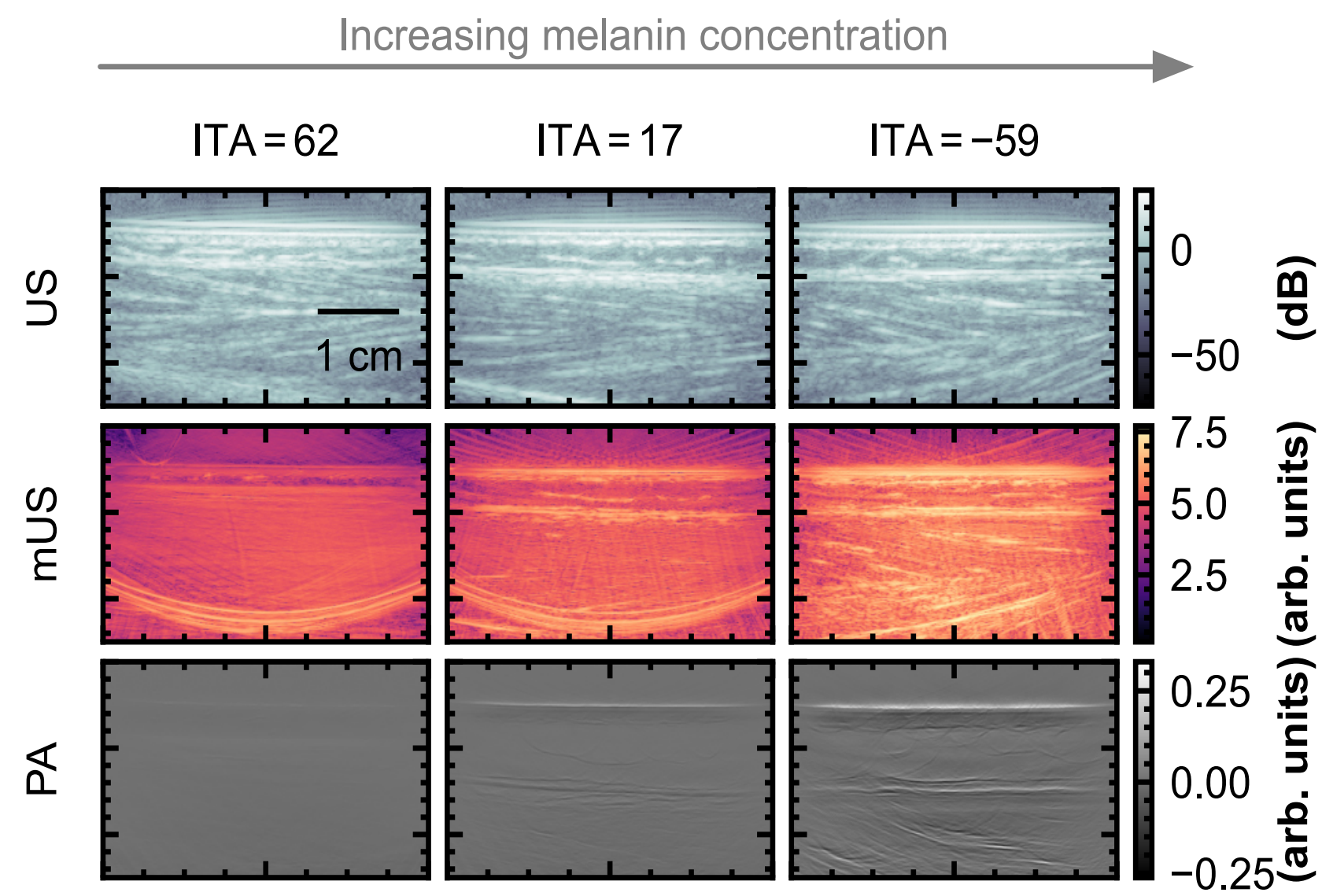

Fig. S13: ***Melanin absorption ultrasound (mUS) images in the calf muscle.*** *Conventional, reflection ultrasound computed tomography (RUCT) images, compared to melanin ultrasound (mUS) images from plane wave ultrasound reconstruction, and photoacoustic (PA) images in scans of the calf muscle in several different subjects with a range of skin tones.*

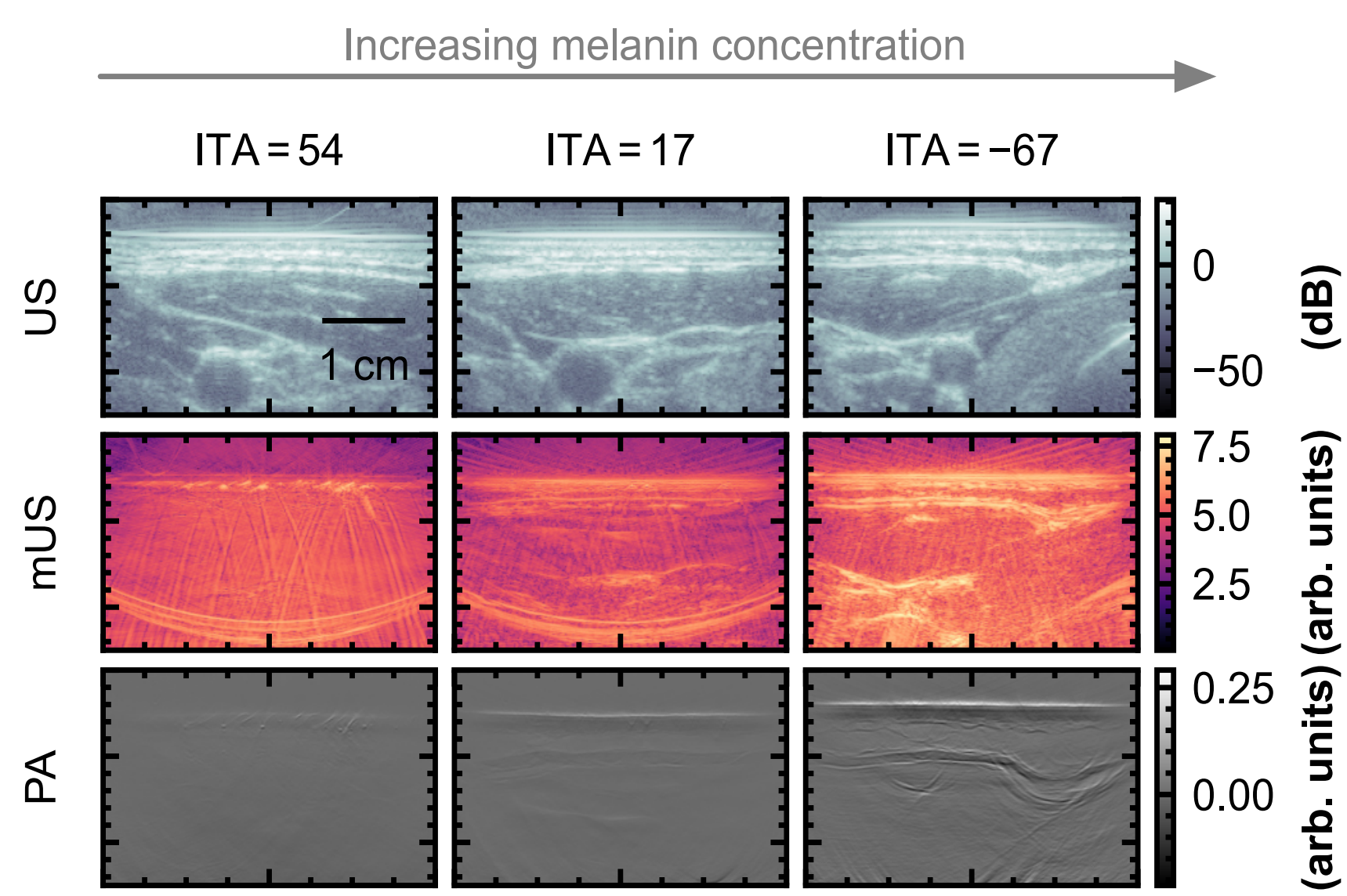

Fig. S14: ***Melanin absorption ultrasound (mUS) images of the carotid artery.*** *Conventional, reflection ultrasound computed tomography (RUCT) images, compared to melanin ultrasound (mUS) images from plane wave ultrasound reconstruction, and photoacoustic (PA) images in scans of the carotid artery in the neck in several different subjects with a range of skin tones.*
