# Supplementary material for: The confounding effects of skin colour in photoacoustic imaging
Source: Nat Commun. 2026 Jun 17;17:7672. doi: 10.1038/s41467-026-74380-7 (PMC13433769; doi:10.1038/s41467-026-74380-7)
Supplement: Supplementary file 2 [file 41467_2026_74380_MOESM2_ESM.pdf]

nature portfolio

Corresponding author(s): Amit Roshan
Sarah Bohndiek

Last updated by author(s): Dec 19, 2025

Reporting Summary

Nature Portfolio wishes to improve the reproducibility of the work that we publish. This form provides structure for consistency and transparency in reporting. For further information on Nature Portfolio policies, see our Editorial Policies and the Editorial Policy Checklist.

Statistics

For all statistical analyses, confirm that the following items are present in the figure legend, table legend, main text, or Methods section.

n/a	Confirmed	
☐	☒	The exact sample size (*n*) for each experimental group/condition, given as a discrete number and unit of measurement
☐	☒	A statement on whether measurements were taken from distinct samples or whether the same sample was measured repeatedly
☐	☒	The statistical test(s) used AND whether they are one- or two-sided<br>*Only common tests should be described solely by name; describe more complex techniques in the Methods section.*
☐	☒	A description of all covariates tested
☐	☒	A description of any assumptions or corrections, such as tests of normality and adjustment for multiple comparisons
☐	☒	A full description of the statistical parameters including central tendency (e.g. means) or other basic estimates (e.g. regression coefficient) AND variation (e.g. standard deviation) or associated estimates of uncertainty (e.g. confidence intervals)
☐	☒	For null hypothesis testing, the test statistic (e.g. *F*, *t*, *r*) with confidence intervals, effect sizes, degrees of freedom and *P* value noted<br>*Give P values as exact values whenever suitable.*
☒	☐	For Bayesian analysis, information on the choice of priors and Markov chain Monte Carlo settings
☒	☐	For hierarchical and complex designs, identification of the appropriate level for tests and full reporting of outcomes
☐	☒	Estimates of effect sizes (e.g. Cohen's *d*, Pearson's *r*), indicating how they were calculated

Our web collection on statistics for biologists contains articles on many of the points above.

Software and code

Policy information about availability of computer code

Data collection	Photoacoustic data was collected using the iThera Medical ViewMSOT software, versions 2.1.1.3 to 2.2.0.1.
Data analysis	Data analysis was conducted using the open source Python toolkit PATATO (github.com/BohndiekLab/patato) and custom-written code (github.com/BohndiekLab/PAISKINTONE). Specific version details for the Python libraries used during analysis can be found in the uv.lock file of the PAISKINTONE GitHub repostory.

For manuscripts utilizing custom algorithms or software that are central to the research but not yet described in published literature, software must be made available to editors and reviewers. We strongly encourage code deposition in a community repository (e.g. GitHub). See the Nature Portfolio guidelines for submitting code & software for further information.

Data

Policy information about availability of data

All manuscripts must include a data availability statement. This statement should provide the following information, where applicable:

- Accession codes, unique identifiers, or web links for publicly available datasets
- A description of any restrictions on data availability
- For clinical datasets or third party data, please ensure that the statement adheres to our policy

Anonymised imaging datasets are available on Zenodo (10.5281/zenodo.20068219).

Anonymised colourimeter data and simulation results are available in the GitHub repository.

Research involving human participants, their data, or biological material

Policy information about studies with human participants or human data. See also policy information about sex, gender (identity/presentation), and sexual orientation and race, ethnicity and racism.

Reporting on sex and gender

Sex was recorded for each participant and is provided in our data tables. Our study cohort consisted of a similar number of male and female participants. Our study addressed only differences in photoacoustic signals with respect to melanin content, so we did not conduct an analysis of differences related to sex.

Reporting on race, ethnicity, or other socially relevant groupings

The Fitzpatrick skin type, an approximate measurement of skin colour was collected for each participant by self-reporting. We used this at the recruitment stage to ensure that we recruited a suitably wide range of participants.

Population characteristics

42 healthy volunteers were recruited to the study. 6 participants were recruited for each Fitzpatrick type and 6 participants with vitiligo were recruited. The mean and standard deviation of the age, and the sex distribution of each group was as follows:

	Age		Sex	BMI (kg/m2)	
FP I	53.5 ± 22.2	(24.0, 76.0)	1 M, 5 F	21.2 ± 1.4	(19.1, 23.1)
FP 2	46.2 ± 21.4	(24.0, 73.0)	3 M, 3 F	24.5 ± 4.0	(18.5, 29.9)
FP 3	31.7 ± 6.4	(25.0, 43.0)	2 M, 4 F	23.6 ± 3.3	(19.7, 27.7)
FP 4	34.0 ± 12.9	(22.0, 57.0)	3 M, 3 F	23.3 ± 2.6	(19.6, 27.2)
FP 5	37.5 ± 15.2	(23.0, 65.0)	3 M, 3 F	23.8 ± 2.8	(20.2, 27.5)
FP 6	34.0 ± 9.5	(26.0, 48.0)	4 M, 2 F	23.8 ± 3.2	(19.6, 27.5)
Vitiligo	59.7 ± 12.7	(40.0, 72.0)	2 M, 4 F	25.0 ± 4.1	(20.2, 28.7)

Recruitment

Participants were recruited by flyers within the University of Cambridge, through invitation by the dermatology clinic of Cambridge University Hospital NHS Foundation Trust, or through invitation by the Alliance for Cancer Early Detection (ACED) cohort. Participants were screened prior to recruitment using a Fitzpatrick type self-assessment form, to ensure that we did not under- or over-recruit for any Fitzpatrick group.

Ethics oversight

PAISKINTONE was approved by the East of England - Cambridge South Research Committee (Ref: 23/EE/0019) and conducted in accordance with the Declaration of Helsinki. Written informed consent was obtained from all study participants. The trial was registered with clinicaltrials.gov: https://clinicaltrials.gov/study/NCT05554523.

Note that full information on the approval of the study protocol must also be provided in the manuscript.

Field-specific reporting

Please select the one below that is the best fit for your research. If you are not sure, read the appropriate sections before making your selection.

☒ Life sciences ☐ Behavioural & social sciences ☐ Ecological, evolutionary & environmental sciences

For a reference copy of the document with all sections, see nature.com/documents/nr-reporting-summary-flat.pdf

Life sciences study design

All studies must disclose on these points even when the disclosure is negative.

Sample size

We estimated a lower bound on the number of participants required to give us enough statistical power based on related pulse oximetry studies and simulations. We then chose a number of participants greater than this based on feasibility (6 from each Fitzpatrick type + 6 with vitilgo, total 42).

Data exclusions

Data from one participant was excluded as we were not able to acquire ultrasound data due to equipment failure. We excluded this participant to reduce potential biases caused by drawing regions of interest on photoacoustic images.

Replication

Imaging phantoms were acquired prior to each participant visit. No substantial drift or variation was observed in the photoacoustic signal in this phantom.

Photoacoustic measurements for each participant for each imaging site were repeated three times with replacement of the imaging probe and ultrasound gel. The reproducibility was quantified in this study and shown as a function of skin colour.

Randomization

Randomisation was not performed. Participants were assigned into group based on an observation of the Fitzpatrick skin type, and individual typology angle.

Blinding

Regions of interest were drawn on ultrasound images, which do not have any skin colour dependency, rather than photoacoustic images to reduce any potential biases.

Full blinding was not possible during data acquisition, however, the imaging operator used the ultrasound images as an anatomical guide, to reduce any potential bias caused by photoacoustic imaging artefacts.

Reporting for specific materials, systems and methods

We require information from authors about some types of materials, experimental systems and methods used in many studies. Here, indicate whether each material, system or method listed is relevant to your study. If you are not sure if a list item applies to your research, read the appropriate section before selecting a response.

Materials & experimental systems

n/a	Involved in the study
☒	☐ Antibodies
☒	☐ Eukaryotic cell lines
☒	☐ Palaeontology and archaeology
☒	☐ Animals and other organisms
☐	☒ Clinical data
☒	☐ Dual use research of concern
☒	☐ Plants

Methods

n/a	Involved in the study
☒	☐ ChIP-seq
☒	☐ Flow cytometry
☒	☐ MRI-based neuroimaging

Clinical data

Policy information about clinical studies

All manuscripts should comply with the ICMJE guidelines for publication of clinical research and a completed CONSORT checklist must be included with all submissions.

Clinical trial registration: https://clinicaltrials.gov/study/NCT05554523.

Study protocol: The full study protocol is available from the corresponding authors.

Data collection: The study started in June 2023 and finished recruitment in August 2024. The study was conducted at the Cambridge Clinical Research Centre at Addenbrookes Hospital in Cambridge, United Kingdom. Participants were invited for a single study.

Outcomes:
Primary outcome measure:
Quantitative photoacoustic imaging: Identify biases in quantitative photoacoustic imaging when scanning people with different Fitzpatrick skin tones and Vitiligo.
Secondary outcome measure:
Correlation of photoacoustic imaging data with pulse oximetry.

Plants

Seed stocks: *Report on the source of all seed stocks or other plant material used. If applicable, state the seed stock centre and catalogue number. If plant specimens were collected from the field, describe the collection location, date and sampling procedures.*

Novel plant genotypes: *Describe the methods by which all novel plant genotypes were produced. This includes those generated by transgenic approaches, gene editing, chemical/radiation-based mutagenesis and hybridization. For transgenic lines, describe the transformation method, the number of independent lines analyzed and the generation upon which experiments were performed. For gene-edited lines, describe the editor used, the endogenous sequence targeted for editing, the targeting guide RNA sequence (if applicable) and how the editor was applied.*

Authentication: *Describe any authentication procedures for each seed stock used or novel genotype generated. Describe any experiments used to assess the effect of a mutation and, where applicable, how potential secondary effects (e.g. second site T-DNA insertions, mosiacism, off-target gene editing) were examined.*
