# Supplementary material for: The confounding effects of skin colour in photoacoustic imaging
Source: Nat Commun. 2026 Jun 17;17:7672. doi: 10.1038/s41467-026-74380-7 (PMC13433769; doi:10.1038/s41467-026-74380-7)
Supplement: Supplementary file 3 [file 41467_2026_74380_MOESM3_ESM.pdf]

nature portfolio

Peer Review File

The confounding effects of skin colour in photoacoustic imaging

Corresponding Author: Dr Sarah Bohndiek

This file contains all reviewer reports in order by version, followed by all author rebuttals in order by version.

Attachments originally included by the reviewers as part of their assessment can be found at the end of this file.

Parts of this Peer Review File have been redacted as indicated to remove third-party material.

Version 0:

Reviewer comments:

Reviewer #1

(Remarks to the Author)
This manuscript builds on recent prior work in PAI to elucidate the effects of biological variations in epidermal melanin content on PAI image quality and oximetry measurements, improve understanding of the mechanisms involved and identify methods for mitigation of these effects during clinical procedures. The authors have performed an impressive clinical study in subjects with a wide range of skin pigmentation levels to generate novel insights. These results are combined with computational modeling and numerical approaches to further analyze significant trends, and a lookup-table based approach is proposed for mitigation of pigmentation effects. Overall, this is a very high quality study, although additional comparisons of their findings with prior studies should be provided, and some of the results could be more rigorously analyzed in order to validate certain conclusions. If these issues are suitably addressed, this manuscript may be acceptable for publication.

1. Abstract. The statement “From this comprehensive dataset, we identified and characterised two physical mechanisms responsible...” seems to imply that this article is the first to identify melanin-based spectral colouring, which is not accurate.

2. Abstract. The text mentions that “strong light absorption by melanin” causes spectral colouring, however, it may be worth mentioning that it is not just the strength of the absorption that matters, but the spectral variation in absorption that is key.

3. Abstract. The text notes that light absorption by melanin leads to spectral colouring, but it fails to then connect this to the important practical outcome of errors in SO2.

4. Abstract. “ identified the backscattering of ultrasound waves generated in the skin...” Stating the specific location of generation – the “epidermis” – would make this statement clearer than using “skin”. (I believe this would be the case in several other locations where “skin” is used as well)

5. P. 4. Para 1. This paragraph mentions "the familiar spectral colouring" which may cause the reader some confusion, as it does not clearly state that this version of spectral colouring is not caused by blood absorption, but rather by melanin (or a combination of melanin and blood absorption).

6. P. 4. The literature review omits mention of highly relevant prior work (Vogt et al., Biomed Opt Express 2023) which used tissue phantoms to provide early insights into melanin effects in PAI including as generalized clutter and discrete acoustic reflection artifacts, spectral colouring, penetration depth reduction and impact on spectral unmixing for blood oxygenation estimates.

7. P. 6. Please clarify which form of the Fitzpatrick approach was used for subject recruitment. The original version of this approach involves a questionnaire focusing on the subject’s tendency to incur sunburn versus tanning – it did not involve assessment of skin tone. The more common approach in recent years has involved a skin color scale that is not well standardized. If the latter, please identify what approaches were used to help ensure that responses were standardized.

8. P. 6. More information should be provided on methods used to perform colorimetry measurements. These measurements can produce spurious results unless known problems are mitigated. For example, were multiple measurements taken on

each subject? Were outliers discarded? What tissue site was measured?

9. Much of the data is presented in "arbitrary units" which does not inspire confidence in the results and conclusions. There are sources of error that could cause day-to-day or inter-measurement variations which a high quality study will always seek to quantify so as to standardize measurements to the degree possible. Was PAI signal not standardized in any way as to make it not completely arbitrary (e.g., daily phantom checks)? If so, please provide some insight into this process, such as how often was such a procedure applied.

10. P. 7 The text states: “the photoacoustic signal in the skin was obtained by drawing a polygonal region of interest (ROI) on the US images and applying it to the photoacoustic images” In this scenario, was the mean pixel value in the polygon used? Was a specific type and size of polygon used consistently?

11. P. 7 The manuscript describes imaging of radial and carotid arteries. However, it fails to provide substantial geometric information on imaged structures being discussed, esp. depth and diameter of these vessels. Such information is critical to understand the results. In all cases where structures are discussed, information on depth and diameter should be provided.

12. P. 8. The text states “Perhaps surprisingly, the overall signal intensity of different structures did not decrease with increasing skin pigmentation, as might be naively predicted” Given the strong absorption properties of melanin – particularly at shorter wavelengths like 700 nm – it is logical to expect decreasing fluence at a given depth with increasing pigmentation, and thus a decrease in signal intensity. A concomitant increase in background noise due to clutter from acoustic reflections would further decrease SNR at chromophore sites (e.g., vessels). The "naive" statement should preferably be removed, and if not, it should be more clearly explained. Additionally, this counterintuitive result should be explained based on optical/physical principles (if that is possible), and compared to prior articles that showed more expected results.

13. Fig. 2A, B. The results in these images were particularly confusing. The 700 nm data in Fig. 2A appears to show a gradual decrease in radial artery detectability with increasing pigmentation, to the point where the background signal/clutter for the two highest pigmentation cases exhibit no clearly identifiable vessel. However, the result in Fig. 2B, and the authors explanation of these results appear to indicate that the vessel was indeed detectable even at ITA = -42, and the results in Fig. 2C indicate no apparent trend in signal intensity with ITA. This is a questionable finding that an unbiased reader will likely believe to be artifactual. A line graph with a larger depth range would likely better illustrate the difference in detectability (contrast-to-noise ratio) than the very narrow depth range selected. Additionally, instead of graphing signal intensity vs. ITA, a more realistic way to assess variations in inclusion conspicuity would be to graph contrast-to-noise ratio (defined as the difference of mean signal in vessel and mean background level, divided by standard deviation of background signal) as a function of ITA.

14. P. 20. The description of colourimetry measurements should be expanded to include basic information such as the wavelength range was used. Additionally, during acquisition process, were any steps taken to ensure that variations in blood content of the skin did not impact measurements? This has been described as a key confounding factor in prior studies.

15. P. 20 The text states that ITA “has been proposed as a quantitative measure of skin colour.” In fact, the justification for using ITA is stronger than this. It has been demonstrated to provide values that are well correlated with epidermal melanin content (e.g., https://doi.org/10.1111/pcmr.12410)

16. P. 20. The pulse duration of the OPO laser pulses should be provided.

17. P. 22. While a melanosome volume fraction of 0.43 was identified by Jacques as an approximate level for highly pigmented skin, PAI is commonly used for breast imaging, and substantial portions of the breast surface can be covered by areolar skin which has a much greater melanin content than other skin regions. Ideally, this study should address the viability of PAI through areolar skin and the potential of the proposed mitigations to overcome the challenge of greater melanin fraction levels that this scenario represents. At very least, the authors should discuss this issue.

18. P. 23. The text notes that 10E7 photons were used per simulation. Some discussion of convergence/noise is warranted here, such as what level of convergence/noise did this produce in your results, especially for deeper regions of the tissue where absorbed photons are more sparse? Was noise level considered in selecting the number of photons launched?

19. P. 23. Was the probe reflectivity accurately modeled? A large fraction of photons likely reflect back out near the source and must be simulated correctly for an accurate result. A highly reflective probe surface may enhance the effect of melanin by increasing the mean number of photon passes through the epidermis, in addition to increasing fluence (whereas a highly absorptive surface may introduce photoacoustic artifacts).

20. Discussion. The Monk Skin Tone (MST) scale is a method based on 10 color levels, which is standardized according to colorimetry values and emerging as the preferred subjective approach for recruitment, particularly in the field of pulse oximetry. The text should discuss whether MST would be more appropriate for future clinical studies than the Fitzpatrick approach.

(Remarks on code availability)

Reviewer #2

(Remarks to the Author)
See attached document.

(Remarks on code availability)

Version 1:

Reviewer comments:

Reviewer #1

(Remarks to the Author)
The authors have provided an extensive reply to author comments and have done an excellent job of responding and making meaningful changes to the manuscript. I believe that these modifications have significantly improved the quality of the manuscript. This is an impressive piece of work and is suitable for publication.

That said, please carefully review the references.

- Many Journal titles are not listed with using standard abbreviations.
- Titles of articles are inconsistently capitalized.
- Reference 31 does not actually provide a citation.
- In reference 39, the first author name appears to be omitted/incorrect.

(Remarks on code availability)

Reviewer #2

(Remarks to the Author)
see attached document

(Remarks on code availability)

Version 2:

Reviewer comments:

Reviewer #2

(Remarks to the Author)
Thanks for being more precise in the words chosen to describe this work. It helps a lot. There are still some confusing points in the response document, which do not affect the overall manuscript interpretation. Here is one example: "We have included this data in Supplementary Figure 1 and refer to this on pg. 5" Where exactly on pg. 5? Aside from issues like this, the paper has been improved overall, and I appreciate all of the detailed responses and clarifications.

(Remarks on code availability)

Open Access This Peer Review File is licensed under a Creative Commons Attribution 4.0 International License, which permits use, sharing, adaptation, distribution and reproduction in any medium or format, as long as you give appropriate credit to the original author(s) and the source, provide a link to the Creative Commons license, and indicate if changes were made.
In cases where reviewers are anonymous, credit should be given to 'Anonymous Referee' and the source.
The images or other third party material in this Peer Review File are included in the article's Creative Commons license, unless indicated otherwise in a credit line to the material. If material is not included in the article's Creative Commons license and your intended use is not permitted by statutory regulation or exceeds the permitted use, you will need to obtain permission directly from the copyright holder.
To view a copy of this license, visit https://creativecommons.org/licenses/by/4.0/

Reviewer #1:

This manuscript builds on recent prior work in PAI to elucidate the effects of biological variations in epidermal melanin content on PAI image quality and oximetry measurements, improve understanding of the mechanisms involved and identify methods for mitigation of these effects during clinical procedures. The authors have performed an impressive clinical study in subjects with a wide range of skin pigmentation levels to generate novel insights. These results are combined with computational modeling and numerical approaches to further analyze significant trends, and a lookup-table based approach is proposed for mitigation of pigmentation effects. Overall, this is a very high quality study, although additional comparisons of their findings with prior studies should be provided, and some of the results could be more rigorously analyzed in order to validate certain conclusions. If these issues are suitably addressed, this manuscript may be acceptable for publication.

We appreciate the enthusiasm of the reviewer for the **"very high quality"** of the study and hope that the changes made below suitably address their remaining concerns.

1. *The statement "From this comprehensive dataset, we identified and characterised two physical mechanisms responsible..." seems to imply that this article is the first to identify melanin-based spectral colouring, which is not accurate.*
    a. We have rephrased this sentence to avoid the noted implication that we are the first to identify spectral colouring:
    b. "From this comprehensive dataset, we characterised the two physical mechanisms responsible for skin colour-dependent degradation in both image quality and haemoglobin biomarker quantification." [Line 31-33]
2. *The text mentions that "strong light absorption by melanin" causes spectral colouring, however, it may be worth mentioning that it is not just the strength of the absorption that matters, but the spectral variation in absorption that is key.*
    a. Indeed, we have now added a note of the wavelength dependence to this sentence:
    b. "Accompanied by detailed theoretical modelling, we demonstrated that strong wavelength-dependent light absorption by melanin leads to spectral colouring, which dominates in individuals with low skin melanin pigmentation." [Line 33-36]
3. *The text notes that light absorption by melanin leads to spectral colouring, but it fails to then connect this to the important practical outcome of errors in sO2.*
    a. Thank you for this comment, we agree that this is very important to note in the abstract. We have now added a sentence to the abstract to reflect this:
    b. "Together, ultrasound backscattering and spectral colouring result in a complex relationship between calculated sO_2 and skin colour; at lower

melanin levels, spectral colouring leads to an increase in sO_2 with increasing melanin, while at very high melanin levels, backscattered ultrasound waves dominate, leading sO_2 to decrease with increasing melanin." [Lines 39-43]

4. "Identified the backscattering of ultrasound waves generated in the skin..." Stating the specific location of generation – the "epidermis" – would make this statement clearer than using "skin".
    a. We have updated the points in the text where we it would be more appropriate to have used epidermis rather than skin. E.g.:
    b. Abstract: "We further identified the backscattering of ultrasound waves generated in the epidermis as a major source of image artefacts for individuals with high skin melanin pigmentation." [Line 38]
    c. In some places, we intentionally used the phrase "skin surface" rather than "epidermis", particularly when segmenting photoacoustic images, as our imaging resolution (~100 um) is approximately the same as the thickness of a typical epidermis. Therefore, it was not possible to say we segmented the epidermis only, but rather a coarser "skin surface" region. In these parts of the text, we have retained the "skin surface" description.
5. This paragraph mentions "the familiar spectral colouring" which may cause the reader some confusion, as it does not clearly state that this version of spectral colouring is not caused by blood absorption, but rather by melanin (or a combination of melanin and blood absorption.)
    a. The original paragraph was intended to highlight the optical and acoustic effects that have been observed in prior studies. We have now separated these more explicitly:
    b. "Image quality impacts primarily appear as ultrasound clutter, increasing in subjects with darker skin and obscuring the desired signal from features like blood vessels. Multispectral analysis is impacted by wavelength-dependent absorption by melanin, which skews spectral unmixing calculations, potentially introducing systematic biases in biomarkers such as blood oxygen saturation." [Lines 77-81]
6. The literature review omits mention of highly relevant prior work (Vogt et al. Biomed Opt Express 2023) which used tissue phantoms to provide early insights into melanin effects in PAI including as generalized clutter and discrete reflection artifacts, spectral colouring, penetration depth reduction and impact on spectral unmixing for blood oxygenation estimates.
    a. Thank you for flagging this oversight. We have now referenced this study. [Line 82].
7. Please clarify which form of the Fitzpatrick approach was used for subject recruitment. The original version of this approach involves a questionnaire focussing on the subject's tendency to incur sunburn versus tanning – it did

not involve assessment of skin tone. The more common approach in recent years has involved a skin colour scale that is not well standardised.

   a. We used the former approach, a questionnaire, as cited in reference 27. https://www.arpansa.gov.au/sites/default/files/legacy/pubs/RadiationProtection/FitzpatrickSkinType.pdf.
   b. We recognise that the Fitzpatrick type is only a coarse proxy measurement of skin colour, but we used it only to pre-screen participants in advance of their in-person study visit. By recruiting 6 participants from each Fitzpatrick type, we were able to ensure we had a wide range of skin types included in the study. The Fitzpatrick type was not used for any quantitative analyses beyond verifying that it gave the expected range of ITA values (skin colour types). All study analyses were performed using ITA values measured using an independent colourimeter (as noted in response to point 8).

8. More information should be provided on methods used to perform colorimetry measurement. These measurements can produce spurious results unless known problems are mitigated. For example, were multiple measurements taken on each subject? Were outliers discarded? What tissue site as measured.
   a. We apologise that the methods were insufficiently detailed on this point; we have now updated the section to clarify where measurements were taken and how many repeats were acquired [Lines 515-520].
   b. Five colorimeter measurements were made over each imaging site. The five were averaged (mean) to give an ITA value for each imaging site. When plots are shown comparing ITA to photoacoustic data, the ITA for the corresponding site was used.
   c. While addressing this question, we uncovered a single outlier in the ITA measurements of participant 25 (radial artery) by plotting the standard deviation of the five measurements against the mean (see figure below). The five measurements taken were: [27.80, 14.49, 25.74, 20.54, -3.32]. The -3.32 entry is 5 standard deviations from the mean of the samples. We therefore removed the -3.32 measurement and recalculated the mean for this entry.

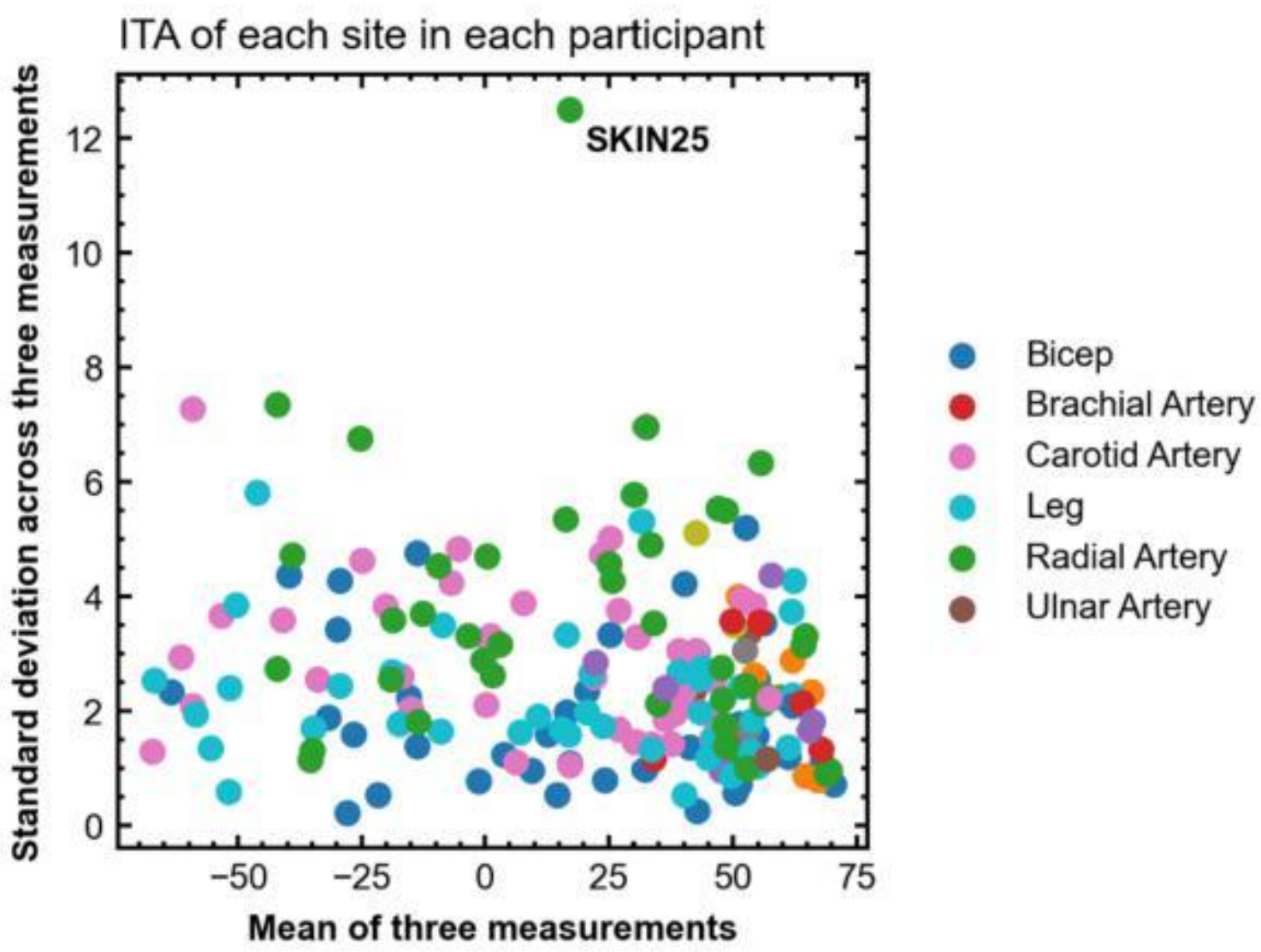

.

d. For completeness, here is the list of changes in the manuscript resulting from this update:
   i. Figure 2C and D had three points each affected by this change. A p-value for the change in standard deviation of PA signal across the radial artery changed in the final decimal place: (p < 0.001 at 700 nm, p = 0.70 at 800 nm, p = 0.97 at 1080 nm)". [Lines 213-214]
   ii. Figure 3: One point changed in scatter plots in 3B, 3C and 3D. P values are slightly altered, with no changes in the significance levels and conclusions. [Lines 239-252]
   iii. Figure 6C. Since one ITA value has changed, the LOESS smooth fit lines in the radial artery subpanel changed ever so slightly. No changes were observed in the statistical tests to the number of decimal places shown.
   iv. Supplementary Figure S9: Four points in sub panel A were affected by this change. The p-values and r values were not affected to the degree of precision given in the document.
   v. Supplementary Figure S10: Two points moved. Does not change the p-values or r values listed to the precision given. 9. Much of the data is

presented in "arbitrary units" which does not inspire confidence in the results and conclusions. There are sources of error that could cause day-to-day or inter-measurement variations which a high quality study will always seek to quantify so as to standardize measurements to the degree possible. Was PAI signal not standardized in any way as to make it not completely arbitrary (e.g., daily phantom checks)? If so, please provide some insight into this process, such as how often was such a procedure applied. a. Indeed, a vendor supplied phantom was scanned before every participant (Supplementary Figure S8). The coefficient of variation of the PA signal in the analysed inclusion was below 10 % for all wavelengths below 1000 nm and no higher than 11.5 % for any

wavelength. The mean coefficient of variation across all wavelengths was 6.8%. These results are included in the main text [Lines 267-276].

b. All photoacoustic data, denoted as arbitrary units, are plotted on a linear scale, which should correspond to initial pressure up to a constant scaling factor. The arbitrary scaling factor appears to be constant over time given the evaluation made of the phantom that was scanned. We have added an additional paragraph to the Materials and Methods section providing more details of the phantom imaging and analysis protocol. [Lines 541-547]

c. We agree that in an ideal world, photoacoustic intensities would be given in absolute pressure units, like Pascals. However, performing a perfect acoustic inversion with typical photoacoustic clinical scanners like ours is impossible, as it would require a knowledge of the speed of sound and acoustic attenuation in the sample, as well as infinitely broadband transducers. We expect, therefore, a constant scaling factor difference between our data and that obtained by similar systems, due to differences in factors like transducer sensitivity. Nonetheless, we have confidence in the consistency of the arbitrary units readout over time based on the phantom referencing included in the manuscript.

10. The text states: “the photoacoustic signal in the skin was obtained by drawing a polygonal region of interest (ROI) on the US images and applying it to the photoacoustic images” In this scenario, was the mean pixel value in the polygon used? Was a specific type and size of polygon used consistently?

    a. The polygon was drawn by hand, so the radial artery region of interest was approximately circular. The muscle polygon was approximately rectangular. Although hand drawn, consistency was maintained through having the same operator draw all regions and using the same shapes for different regions of quantification. The mean pixel value within the polygon was used, however, pixels with negative values at any wavelength were excluded from the calculation, unless otherwise mentioned in the text.

    b. We have added detail regarding variation in the region of interest in response to point 11.

11. The manuscript describes imaging of radial and carotid arteries. However, it fails to provide substantial geometric information on imaged structures being discussed, esp. depth and diameter of these vessels. Such information is critical to understand the results. In all cases where structures are discussed, information on depth and diameter should be provided.

    a. Thank you for this suggestion, we agree that this would provide important context for readers. We have now included information about the depth and diameter of the vessels, as well as the muscle, in the Results section. We additionally confirmed that there was no systematic relationship between ITA and region of interest depth.

b. "The regions of interest analysed showed some variation in size and depth (mean ± standard deviation): radial artery diameter 8.5 ± 1.0 mm, depth 2.9 ± 0.8 mm; carotid artery diameter 10 ± 4 mm, depth 15 ± 3 mm. The centroid of the bicep muscle region was at a depth of 14.2 ± 1.4 mm, while that of the calf muscle was 14.3 ± 1.6 mm. Muscle regions of interest included as much of the muscle as was visible in the field of view." [Lines 163-167]

12. The text states, "Perhaps surprisingly, the overall signal intensity of different structures did not decrease with increasing skin pigmentation, as might be naively predicted" Given the strong absorption properties of melanin – particularly at shorter wavelengths like 700 nm – it is logical to expect decreasing fluence at a given depth with increasing pigmentation, and thus a decrease in signal intensity. A concomitant increase in background noise due to clutter from acoustic reflections would further decrease SNR at chromophore sites (e.g., vessels). The "naive" statement should preferably be removed, and if not, it should be more clearly explained. Additionally, this counterintuitive result should be explained based on optical/physical principles (if that is possible) and compared to prior articles that showed more expected results.
    a. Our naïve statement was coming from solely optical considerations, but we appreciate that acoustic considerations should be included on an equal footing, so we have clarified this point in the Results section [Lines 198 onward].
    b. Since this relates to both our unmixing results and our single-wavelength results, we have added a clearer explanation of the optical effects of melanin absorption in the epidermis to the Discussion section:
    c. "At lower melanin levels (ITA ~>10), our observations are consistent with previous theoretical work, in which optical absorption by melanin in the epidermis leads to a large reduction in light fluence at shorter wavelengths and less so at longer wavelengths, predicting a decrease in photoacoustic signal intensity and an increase in unmixed blood oxygenation with increasing melanin" [Lines 408-412]
13. Fig. 2A, B. The results in these images were particularly confusing. The 700 nm data in Fig. 2A appears to show a gradual decrease in radial artery detectability with increasing pigmentation, to the point where the background signal/clutter for the two highest pigmentation cases exhibit no clearly identifiable vessel. However, the result in Fig. 2B, and the authors explanation of these results appear to indicate that the vessel was indeed detectable even at ITA = -42, and the results in Fig. 2C indicate no apparent trend in signal intensity with ITA. This is a questionable finding that an unbiased reader will likely believe to be artifactual. A line graph with a larger depth range would likely better illustrate the difference in detectability (contrast-to-noise ratio)

than the very narrow depth range selected. Additionally, instead of graphing signal intensity vs. ITA, a more realistic way to assess variations in inclusion conspicuity would be to graph contrast-to-noise ratio (defined as the difference of mean signal in vessel and mean background level, divided by standard deviation of background signal) as a function of ITA.

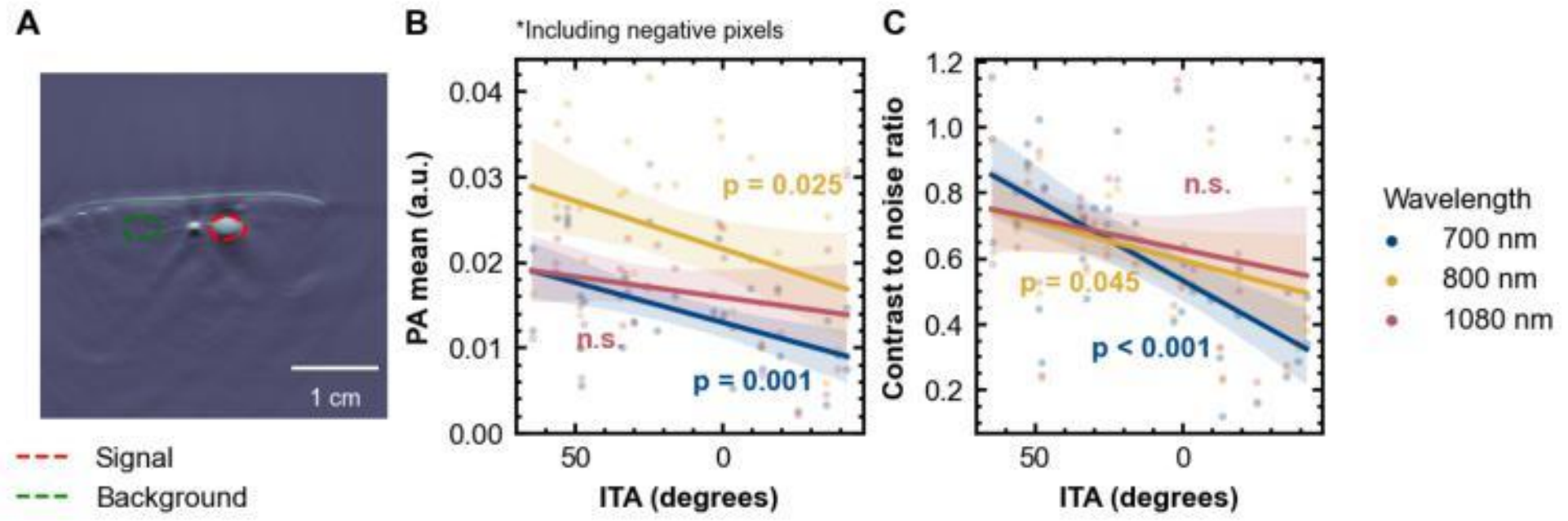

a.
d. The discrepancy appears because the analyses in Figure 2 excluded negatively valued pixels, which arise as artefacts in photoacoustic image reconstruction due to acquisition limitations such as transducer bandwidth. Excluding the negative pixels, however, results in a quantification which is at odds with the visible reduction in radial artery intensity with increasing melanin level in Figure 2A, as the reviewer has correctly noted.
e. To improve the clarity in this section, we have added Supplementary Figure 7, where negative values were included in the analysis (B). Furthermore, we constructed a background region of interest at the same depth as the radial artery (A) and calculated the contrast to noise ratio as a function of ITA (as now detailed in the Materials and Methods). Performing the analysis in this way results in a trend that is consistent with the observation made in Figure 2 (C).
f. We have added a discussion of contrast to noise ratio to the results section [Lines 203-211], and to the Methods [Lines 594-599].

14. P. 20. The description of colourimetry measurements should be expanded to
include basic information such as the wavelength range was used. Additionally, during acquisition process, were any steps taken to ensure that variations in blood content of the skin did not impact measurements? This has been described as a key confounding factor in prior studies.
   a. See point 8.

15. P. 20 The text states that ITA “has been proposed as a quantitative measure of skin colour.” In fact, the justification for using ITA is stronger than this. It has been demonstrated to provide values that are well

correlated with epidermal melanin content (e.g., https://doi.org/10.1111/pcmr.12410) a. Indeed, we have now updated this sentence accordingly “The colourimeter returns the reflected colour in several colour space representations (CIE-L*a*b*, CIE-L*C*h, CIE-XYZ) and several derived

quantities, including the individual typology angle (ITA, Equation 1), a quantitative measure of skin colour [40]." [Lines 523-526]

16. P. 20. The pulse duration of the OPO laser pulses should be provided.
    a. We have added a note of laser pulse duration (4-7 ns) to the Methods section. [Line 537]
17. P. 22. While a melanosome volume fraction of 0.43 was identified by Jacques as an approximate level for highly pigmented skin, PAI is commonly used for breast imaging, and substantial portions of the breast surface can be covered by areolar skin which has a much greater melanin content than other skin regions. Ideally, this study should address the viability of PAI through areolar skin and the potential of the proposed mitigations to overcome the challenge of greater melanin fraction levels that this scenario represents. At very least, the authors should discuss this issue.
    a. We appreciate that PAI through areolar skin would constitute an even more challenging application of photoacoustic imaging due to the presence of additional skin pigmentation, particularly since breast imaging is one of the approved clinical applications of photoacoustic imaging. It is, unfortunately, not feasible for us to acquire additional data in this context as this would require new clinical research ethical approval.
    b. We have added a comment to the Discussion section emphasising the need for further study in this area. "Additional variation in skin surface pigmentation, for example, increased pigmentation associated with scar tissue or darker areolar skin in breast imaging, or decreased pigmentation with regression in melanoma imaging, could compound these effects." [Line 472-474]
18. P. 23. The text notes that 10E7 photons were used per simulation. Some discussion of convergence/noise is warranted here, such as what level of convergence/noise did this produce in your results, especially for deeper regions of the tissue where absorbed photons are more sparse? Was noise level considered in selecting the number of photons launched?
    a. We were only interested in the mean fluence across the blood vessel, rather than very deep regions, so we did not expect noise to be significant. To test this prediction, we ran the simulation for the highest melanosome volume fraction (0.4) and longest wavelength (700 nm), where we expect the noise level to be most pronounced and saw less than a 1% difference between 10^7 photons and 10^8 photons, suggesting that we had adequate convergence by 10^7 photons.
    b. "To confirm convergence of our simulations, we evaluated the difference in fluence in the simulated blood vessel obtained between 10^7 and 10^8 photons in our simulation with highest melanosome concentration, observing only a 1% difference." [Lines 611-614]

19. P. 23. Was the probe reflectivity accurately modeled? A large fraction of photons likely reflect back out near the source and must be simulated correctly for an accurate result. A highly reflective probe surface may enhance the effect of melanin by increasing the mean number of photon passes through the epidermis, in addition to increasing fluence (whereas a highly absorptive surface may introduce photoacoustic artifacts).
    a. We did not explicitly model any optical properties of the probe itself, for example, the “mediprene” pad that contacts the skin, or the gel pad. We were unable to find publicly available data regarding the optical properties of these materials that could be included into a model. Empirically, we do not see any substantial photoacoustic signal in the probe itself, which implies it would not have a substantial impact on the fluence model.
20. Discussion. The Monk Skin Tone (MST) scale is a method based on 10 color levels, which is standardized according to colorimetry values and emerging as the preferred subjective approach for recruitment, particularly in the field of pulse oximetry. The text should discuss whether MST would be more appropriate for future clinical studies than the Fitzpatrick approach.
    a. In our study, the Fitzpatrick type was used only for the purposes of recruitment and appears to have led to a distribution of skin pigmentation levels that is roughly uniform with respect to the logarithm of melanosome volume fraction (see our response to Reviewer 2, point 5). It is possible that using the Monk scale might have enabled us to recruit a more uniform distribution of melanosome volume fractions and this has now been acknowledged in the manuscript.
    b. “Our recruitment was based on the Fitzpatrick scale, giving approximately uniform recruitment in terms of the logarithm of melanosome volume fraction. However, alternative qualitative metrics, such as the Monk scale, could enable more uniform recruitment with respect to skin type without increased complexity in the recruitment process.” [Lines 451-456].

Reviewer #2:

This paper reports a timely and relevant topic. Much more research is needed to shed light on the asymmetrical impact of skin tone on photoacoustic imaging system

performance. I commend the authors for tackling this problem. The most interesting aspects of this work are the performance observed in muscle data, vitiligo patients, and the method introduced to address the noted problems.

We thank the reviewer for their detailed assessment of our study on **"a timely and relevant topic"**. We have carefully reviewed the feedback and hope that our responses, as well as the updates within the manuscript, will help to address the concerns of the reviewer.

Other aspects are not new and can be deemphasized to avoid overstating the importance, novelty, and significance of this work, which will otherwise call into question the entire body of work. This is a major concern and noted weakness of the presentation, as described in more detail below.

1. Abstract: "We further identified the backscattering of ultrasound waves generated in the skin as a major source of image artefacts for individuals with high skin melanin pigmentation." This is not a new finding and it has been demonstrated before. See for example the introduction section of the work by reference 8 (Fernandes et al. Photoacoustics 2023). The claim here needs to be toned down.
    a. As noted by the reviewer, and in our manuscript, the prior work by Fernandes et al studies "the impact of epidermal melanin content on conventional amplitude-based PA images, as well as the capability of SLSC beamforming to reduce clutter artifact". Their study indeed established the increased presence of clutter in volunteers with darker skin tones and demonstrated the added value of SLSC as an approach to mitigate the artefact. They showed a strong correlation between the level of clutter present and the ITA value. Furthermore, previous studies such as Singh et al. 2016 also demonstrated that the epidermis acts as a source of ultrasound waves which are backscattered, leading to clutter formation.
    b. We go beyond correlation in our study, confirming the physical origin of the clutter. Firstly, we demonstrate the production of acoustic clutter from melanin absorption in **several different anatomical areas**; only the radial artery has been studied before. Secondly, we conduct multiple independent analyses to **demonstrate the underlying mechanism of clutter formation,** through simulations, plane wave ultrasound reconstruction, photoacoustic signal heterogeneity analysis and multispectral analysis of the clutter.
    c. To avoid implying that the specific finding of increased clutter as a function of skin tone is new, we have rephrased the quoted sentence "We further demonstrated the backscattering of ultrasound waves generated in the skin as a major source of image artefacts for individuals with high skin melanin pigmentation.".

d. The key advance of our study is to experimentally and computationally demonstrate that the **source** of the clutter is ultrasound waves generated in the epidermis.
e. In addition, as far as we know, no previous study has provided convincing evidence that linked the clutter to its effects on quantification, including spectral unmixing, as we have here, providing another key advance in our study compared to prior art.

2. Introduction: “a comprehensive assessment of the confounding effects of skin colour has yet to be performed for PAI.” There are multiple papers that show the confounding effects of skin color. For example, references 8, 9, 10, 11, and:
    a. Fernandes, Guilherme S. Pilotto, et al. "Impact of skin pigmentation on photoacoustic imaging using linear array transducer: a pilot in vivo study." 2022 IEEE International Ultrasonics Symposium (IUS). IEEE, 2022.
    b. Fernandes, Guilherme SP, et al. "In silico demonstrations of the impact of wavelength and skin tone on photoacoustic breast imaging." 2023 IEEE International Ultrasonics Symposium (IUS). IEEE, 2023.

    Could the authors clarify what about all of this work is not considered comprehensive? This will help to maintain historical accuracy and to ensure proper claims are being made.

    While the studies cited have each contributed to an increasing body of evidence demonstrating skin tone bias in photoacoustic imaging, there are several substantial questions that have not been resolved in prior studies:
    a. First, all previous in-human studies have looked at the single anatomical site of the radial artery, making generalisation of findings limited. **We studied multiple anatomical sites including major blood vessels and muscles.**
    b. Second, the influence of skin pigmentation on photoacoustic derived oximetry has only been considered in the study of Mantri et al. That study obtained data from only 9 participants, so had limited representation of different skin tones. They also used an LED-based system at two wavelengths, which limits the penetration depth for imaging as well as the accuracy of unmixing[1]. **We studied 42 participants using a laser-based multiwavelength system, acquiring data at several wavelengths from 660 nm to 1300 nm, at depths of up to 3cm.**
    c. Thirdly, while clutter has been explored previously, the source of clutter has not been unequivocally demonstrated. **As noted above in**

[1] Joseph, J., Ajith Singh, M. K., Sato, N. & Bohndiek, S. E. Technical validation studies of a dual-wavelength LED-based photoacoustic and ultrasound imaging system. Photoacoustics 22, 100267 (2021).

response to (2), we sought to demonstrate the source through multiple methods.

d. Finally, the effects of acoustic clutter on spectral unmixing have not been explored. No study has been able to explain the combined effects of clutter and spectral colouring. **We undertook a detailed optical and acoustic physics-based modelling approach to examine the combined effects of spectral colouring and clutter on photoacoustic data.**

We have updated our Introduction to specifically highlight the limitations in the existing body of literature that we sought to overcome with a comprehensive assessment (lines 75-88).

3. "Here, we present the first comprehensive assessment of the confounding effects of skin tone in PAI." This is not a true statement. See comment #2 for details, discuss the noted references, and state what is actually "first" given this existing literature. Any of the papers noted in comment #2 can be considered among the first comprehensive assessments of the confounding effects of skin tone in PAI as far as I am aware.

   The reasons we considered this the first comprehensive study are discussed in the response to points 1 and 2. To avoid being misconstrued as the first study of skin colour bias in photoacoustics, we have rephrased this sentence: <u>"Here, we present a comprehensive assessment of the confounding effects of skin tone in PAI." [Line 100-101]</u>

4. "For individuals with very high skin pigmentation, image clutter is shown to originate from the skin acting as an ultrasound transducer, with photoacoustic waves generated in the skin being backscattered throughout the image field." This was already reported in the work by Fernandes et al. Photoacoustics 2023 (see quote from this paper below):
   a. "First, the illumination beam strikes the skin, which can be characterized by high optical absorption coefficient in the visible and near-infrared range, due to the melanin content in the epidermis layer [17,18]. This light absorption can considerably affect the illumination efficiency, particularly for deep-located structures in the imaging plane. Second, the PA waves generated due to light absorption at the skin surface can cause the skin to act as an US emitter, transmitting sound waves beyond the skin surface, which are then reflected by typical US scatterers within tissue, thus creating considerable artifacts in PA images [19]. In particular, PA waves generated by strong optical absorbers at the skin surface interact with the surrounding media and

travel back to the US transducer, introducing an artifact known as acoustic clutter."

Hence, this paper needs to be cited at the end of the quoted statement in the paper under review. And, the claim can perhaps be that the authors are providing additional evidence of this. However, the evidence is questionable, as noted above with more details provided later in this review.

In the quoted text, we are noting that we show in our data substantial additional evidence regarding the source of the ultrasound clutter. While Fernandes et al demonstrate a correlation between ITA and clutter level, they do not directly show that this is caused by acoustic reflections, as we have done here.

5. "... suggesting that the Fitzpatrick scale was a suitable proxy for skin colour." This is not true for the darker skin tones. The Fitzpatrick scale lumps multiple dark skin tones into a single value. This is not a suitable proxy for darker skin tones, and this limitation needs to be more clearly acknowledged. This major concern is supported by the higher ITA standard deviations in Table 1 and max/min values in Fig. 1E with the darker skin tones. Similarly, the darker skin tone results presented in Fig. 1G further supports this, as these data points do not follow the linear fit. What is the result when they are fitted separately?

We agree with the reviewer's description of the limitations of the Fitzpatrick scale. In our study, the Fitzpatrick scale was used only for recruitment of participants and ITA was used subsequently for quantitative analyses.

We observe that melanosome volume fraction seems to grow exponentially with Fitzpatrick type. Our recruitment strategy gave uniform coverage of log(melanosome volume fraction), rather than linear coverage. Our histogram of melanosome volume fraction on a linear and log scale shows this nicely below (and new Supplementary Figure 1). We have included a discussion of the limitations of the Fitzpatrick scale in the discussion section, noting the additional potential of the Monk scale [lines 451-455]. Nevertheless, the cohort recruited in this study is the largest ever assembled for this purpose. The cohort includes participants from a wider range of ITA values than any previous photoacoustic study in which ITA has been reported, giving us excellent statistical power.

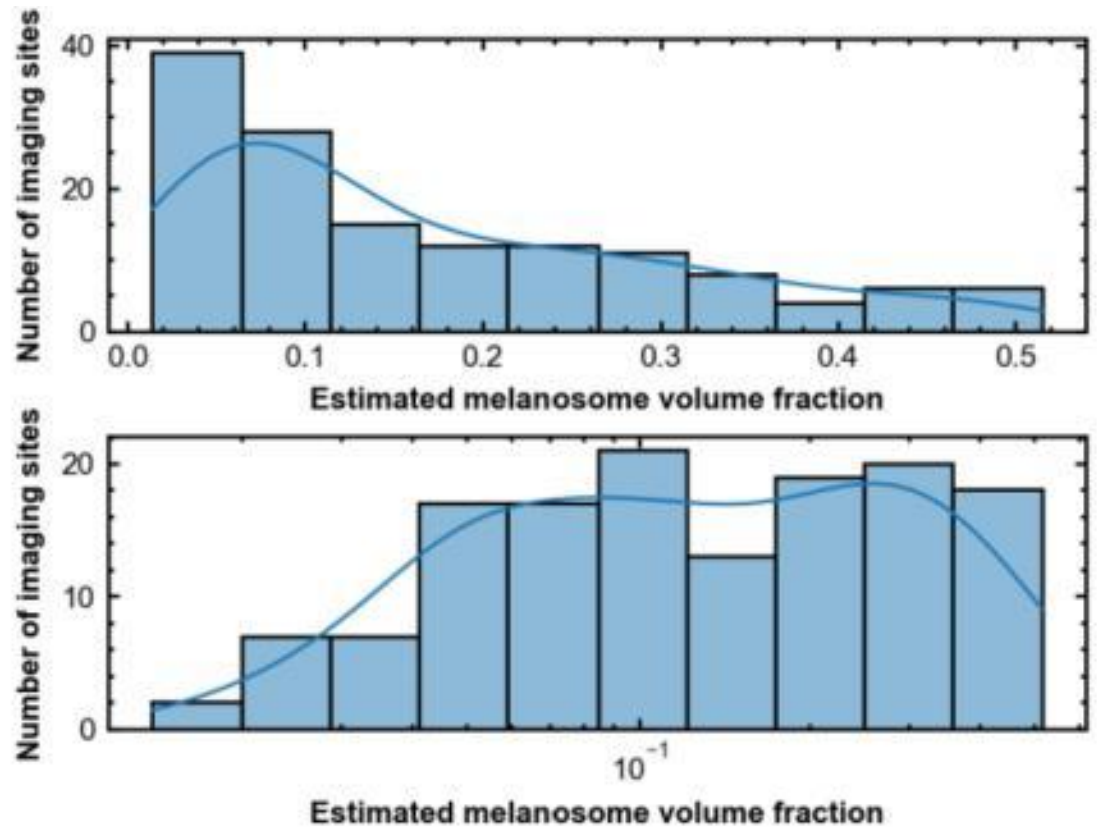

Figure: Histogram of the number of imaging sites as a function of estimated melanosome volume fraction (derived from ITA as described in the paper). This is shown on a linear (top) and log scale (bottom).

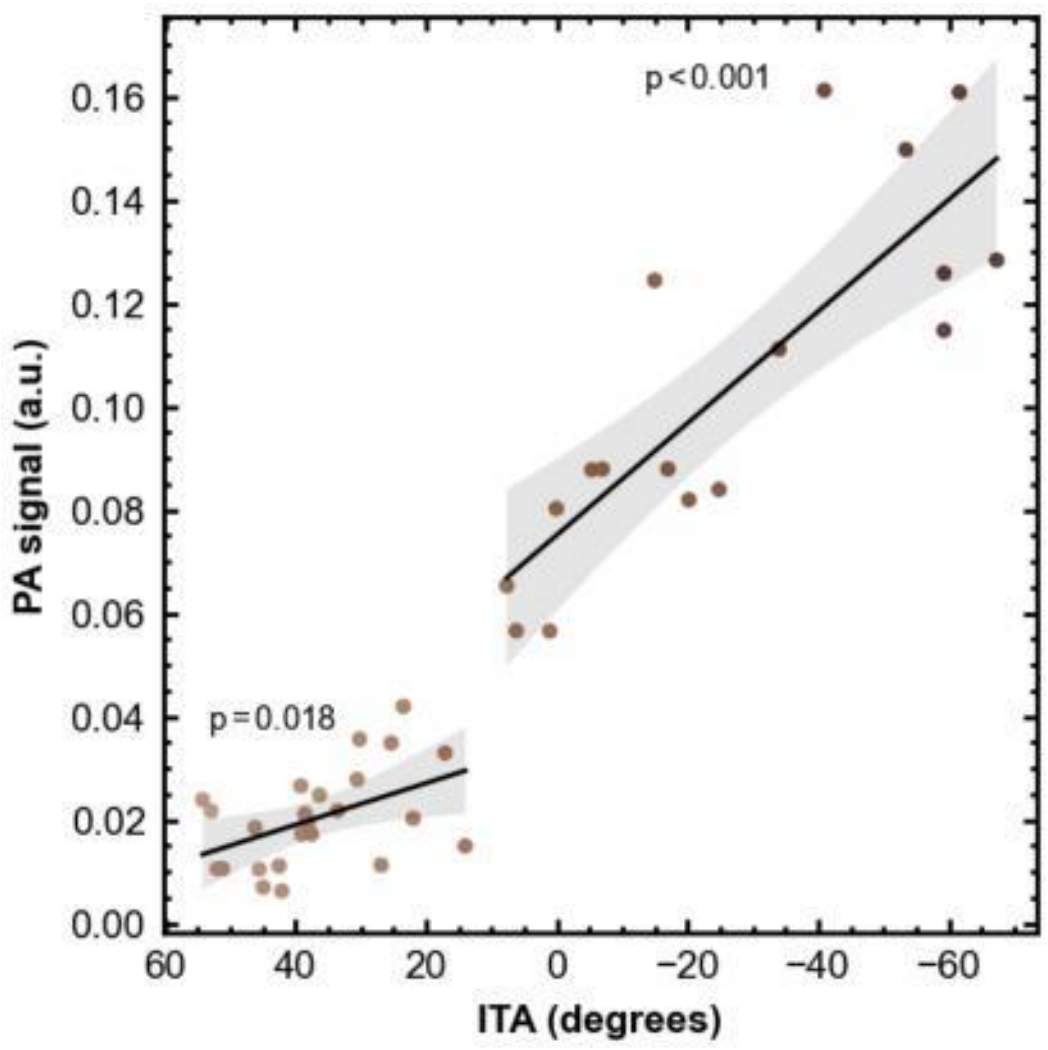

The linear trend in Figure 1G does continue with high statistical significance in participants with ITA < 10 and ITA > 10 separately (by Fernandes et al. 2023's nomenclature this corresponds to groups I-IV and V+VI respectively). The graph includes only measurements from the neck, which was chosen as a sun-exposed area. The increased variability appears to be due to a constant percentage variability in the PA signal in the skin surface, which can be seen when plotted on a log scale. Here all imaging sites are included and we fitted a LOWESS smooth fit with bootstrapping to calculate the confidence interval.

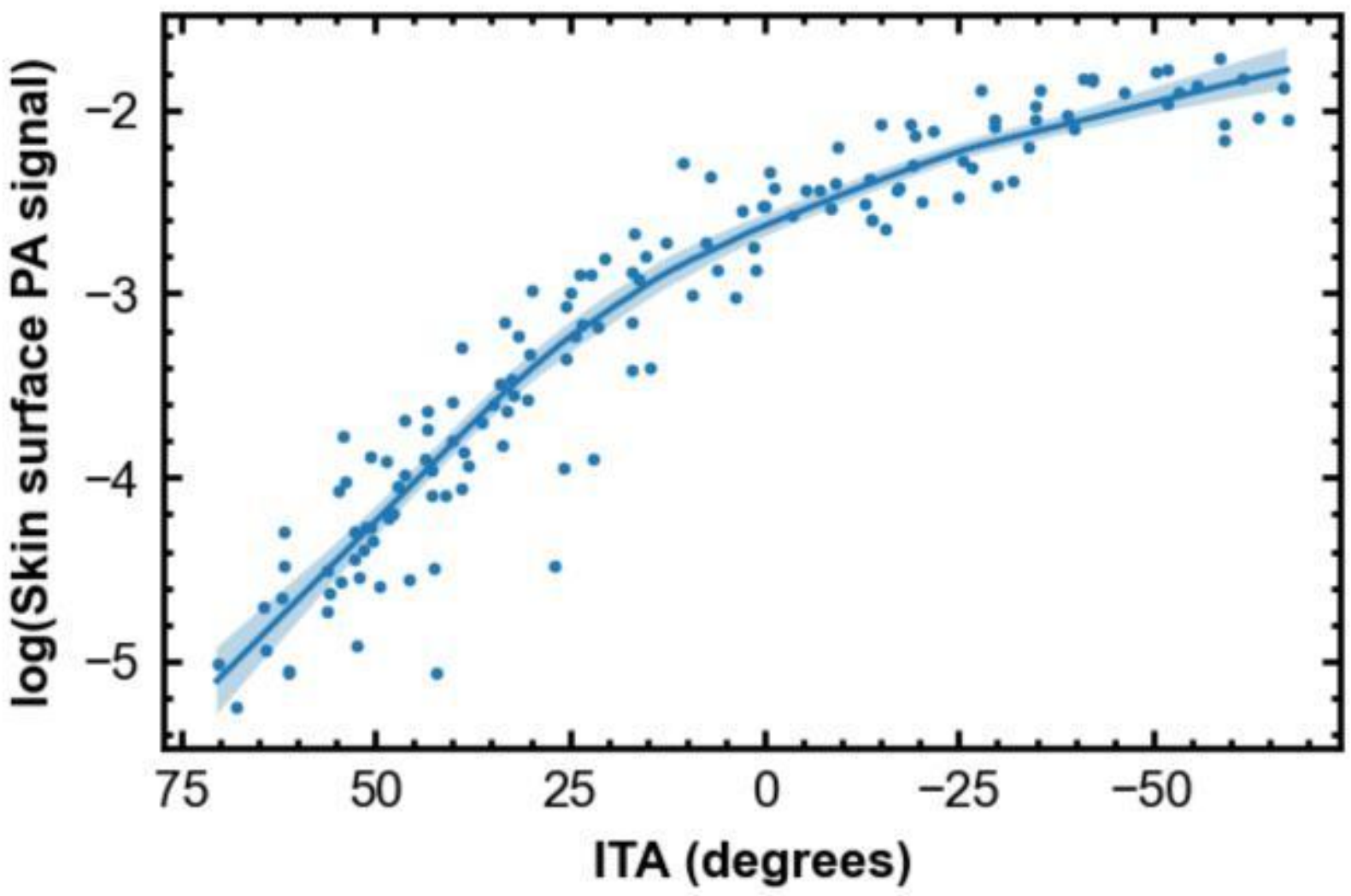

Despite the limitations of the Fitzpatrick scale, our recruitment strategy obtained by far the largest number of participants with low ITA values of any photoacoustic study in which that metric has been reported. For example, in comparison to Fernandes et al 2023, we have more than double the number of participants with ITA < 10 and more than double the number of participants with ITA < -30. This has given us excellent statistical power to demonstrate the complex, non-linear relationship between skin colour and photoacoustic signals.

6. What do the circles in Fig. 1E represent?

   The boxplots in Figure 1E show a solid line representing the median, the upper and lower quartiles as filled box, the "whiskers" represent the upper and lower quartiles +/- 1.5 * interquartile range. Points outside of this range are denoted by circles. We have added a note about this to the Methods section and the figure caption.

7. "We confirmed the relationship between photoacoustic signal from the skin surface and skin pigmentation quantified by colorimetry." Fernandes et al. Photoacoustics 2023 already showed this, and this should be acknowledged. See Fig. 3 from this paper.

   We have added a citation here.

8. "In forearm scans, the radial artery was clearly distinguished at long wavelengths..." Which wavelengths are the authors considering "long"? Please specify specific wavelengths in this sentence.

   We have now specified in the Results section that long is considered >= 950 nm. We quantified this by evaluating the correlation between ITA and contrast-

to-noise ratio for each wavelength. There is a significant correlation for all wavelengths below 950 nm ($p<0.01$), which is not present at longer wavelengths ($p>0.1$).

9. In general, the findings about the radial artery in this paragraph were previously shown by Fernandes et al. Photoacoustics 2023. For example, "Conversely, it was entirely obscured by clutter (very dark or very light arc-shaped features below the skin surface in the images) in participants with darker skin, particularly at shorter wavelengths (Fig. 2A)." This has been shown and is not a novel finding. It would be more accurate to report as a confirmation of previously reported results.

   Indeed, Fernandes et al 2023 do show substantial clutter signal below the skin surface. We have now updated the Results section to note that this confirms observations made in their study.
   "Conversely, as noted previously [8], it was entirely obscured by clutter in participants with darker skin, particularly at shorter wavelengths (Fig. 2A). Unlike prior work where clutter showed a speckle-like appearance [8], here we note the presence of very dark or very light arc-shaped features below the skin surface in the images." [Lines 172-176]

10. "Our qualitative assessment suggested that the forearm was the most informative site for understanding the confounding effects of skin colour due the presence of the radial artery as a focal feature, along with background tissue and bone." Considering that the majority of the paper is based on the radial artery results, the novelty and significance of the entire paper (relative to the work by Fernandes et al. Photoacoustics 2023) is called into question. I recommend de-emphasizing results that have already been shown and instead focus on emphasizing what is truly new in this work.

    We agree that a key importance of our work is the results from other imaging sites in this paragraph, particularly relating to the bicep muscle. We underplayed this slightly in the text, but in fact all the analysis shown in subsequent figures includes both the radial artery and the bicep muscle, with other sites included in the supplementary information for clarity.

    In addition to the study of multiple sites, our radial artery data adds substantially to prior work given the larger cohort size of this study and our multispectral analysis, combined optical and acoustic modelling, fluence correction, and melanin ultrasound reconstruction.

    We have therefore updated our manuscript to place our results in the bicep muscle and the radial artery on an equal footing. We have included more

images from the bicep muscle in Figure 2 and re-emphasised these results in the results section.

11. “Perhaps surprisingly, the overall signal intensity of different structures did not decrease with increasing skin pigmentation, as might be naively predicted.” This is not surprising based on the figure below (Fig. 5), taken from Fernandes et al. Photoacoustics 2023. Part (d) of this figure shows no statistically significant differences in the radial artery (RA) signal as a function of skin tone. There is no need to make a naive prediction about this given this supporting data.
    a. Please see our response to point 12 below. We have updated the phrase to remove “Perhaps surprisingly”.

12. “in the radial artery, there was a negligible change in signal at several wavelengths used for blood vessel imaging in commercial devices” This was also shown in part (d) of the figure below (Fig. 6), taken from Fernandes et al. Photoacoustics 2023:
    a. This is true, but interestingly, when we responded to Reviewer 1’s point 13, we calculated the corresponding plot to Figure 2C that included negatively valued pixels and the corresponding plot for the contrast to noise ratio. In this new analysis, we do see a decrease in photoacoustic signal with increasing melanin level, as predicted by optical considerations. We think this happens because the clutter signal is oscillatory (Figure 2B), cancelling out when negative pixels are included, but contributing positively when negative pixels are excluded.

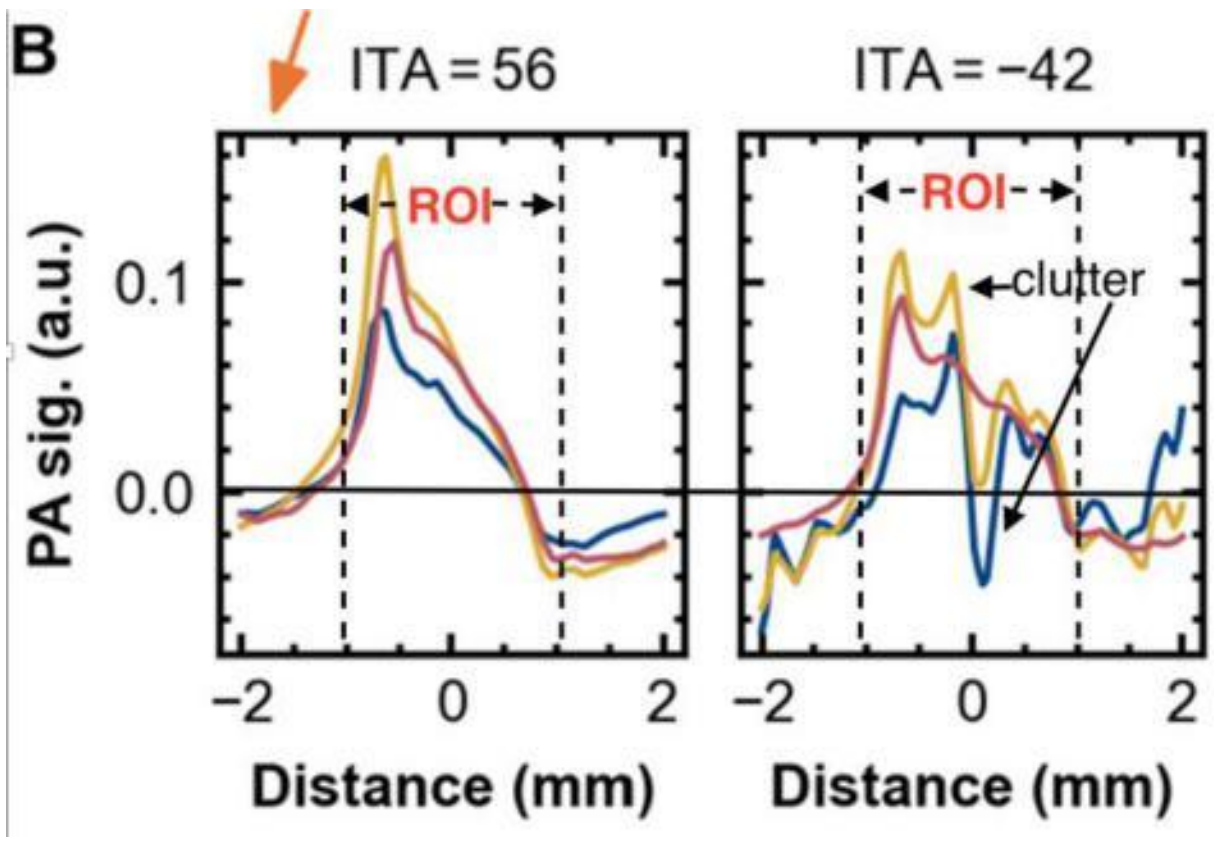

    b.
    f. The negative component of the clutter labelled on the right above would be excluded under the original analysis, while the positive clutter peak would be included, compensating for the fluence-induced reduction in signal intensity when negative pixels are included. This wasn’t demonstrated in Fernandes et al 2023.

13. “The standard deviation of the signal across the radial artery increased significantly at 700 nm, in line with the observations of increased clutter...” I

do not understand the comparison being made here. Increased significantly relative to what? And, why is this reflective of increased clutter. More details are needed to more clearly convey this thought.

We apologise for the lack of clarity on this point. The standard deviation of the signal across the radial artery and bicep were calculated to demonstrate the presence of increased signal variation caused by clutter artefacts.

We can see that there is substantial variation in the signal intensity in the radial artery in Figure 2B (also reproduced in response to point 12), so we showed that the standard deviation gives us a measure of clutter level. We have updated the Results section text on page 8 to more clearly articulate this.

14. "All participants in the vitiligo cohort would otherwise have been placed in Fitzpatrick types I, II, or III..." Patients with Fitzpatrick types IV, V, and VI will suffer the most from the effects being reported. It would be more impactful to show how the reported methods alleviate this concern in patient cohorts with vitiligo and Fitzpatrick types IV, V, and VI. Could the authors recruit more patients to show this impact?

We agree that patients with Fitzpatrick type IV, V and VI would suffer from these effects more. Despite our best efforts, we could not recruit anyone with vitiligo with Fitzpatrick type IV/V/VI. We agree that this would be more impactful, but this is an unfortunate consequence of the demographics of the region in which the study was conducted. We are not able to recruit more patients as we have come to the end of our ethical approval period.

15. In Table 1 and S1, the multiple ITA values measured on the patients with vitiligo should be reported. It is not clear what patch of color is being reported in Table 1 or S1. Why is there only one ITA value in Table S1, when these patients have at least two ITA values for different patches of skin?

Our intention was to provide a single representative ITA value of a sun-exposed area as an indication of the distribution of skin types represented in our dataset. We agree that it would be helpful to include ITA values for more imaging sites and for pigmented and non-pigmented regions in participants with vitiligo. We have now updated Table 1, S1 and added a Table S2 to include representative values from all of the sites measured, including multiple values for the participants with vitiligo where relevant.

16. "Photoacoustic signals from the skin surface region at the lowest wavelength of 700 nm (highest melanin absorption) increased only modestly between

non-pigmented regions and pigmented regions, likely because of absorption from other chromophores like haemoglobin (p = 0.059, linear mixed effects model; Fig. 4D).” Is this expected to be true for lower ITA values? I am doubtful about this. More evidence is needed to support this claim for lower ITA values.

We agree with the reviewer that this trend would not continue for lower ITA values. This result only applies because we are considering very high ITA values in the vitiligo cohort. If we compare Fig. 4D to Fig. 1G (right panel), you can see that all but the very lowest ITA point is below 20, which is in the high ITA region of Fig. 1G where there is only a weak change in PA signal.

17. Is the correlation between the ITA value of one patch color vs. another and the underlying photoacoustic signals similar or different as ITA values decrease?

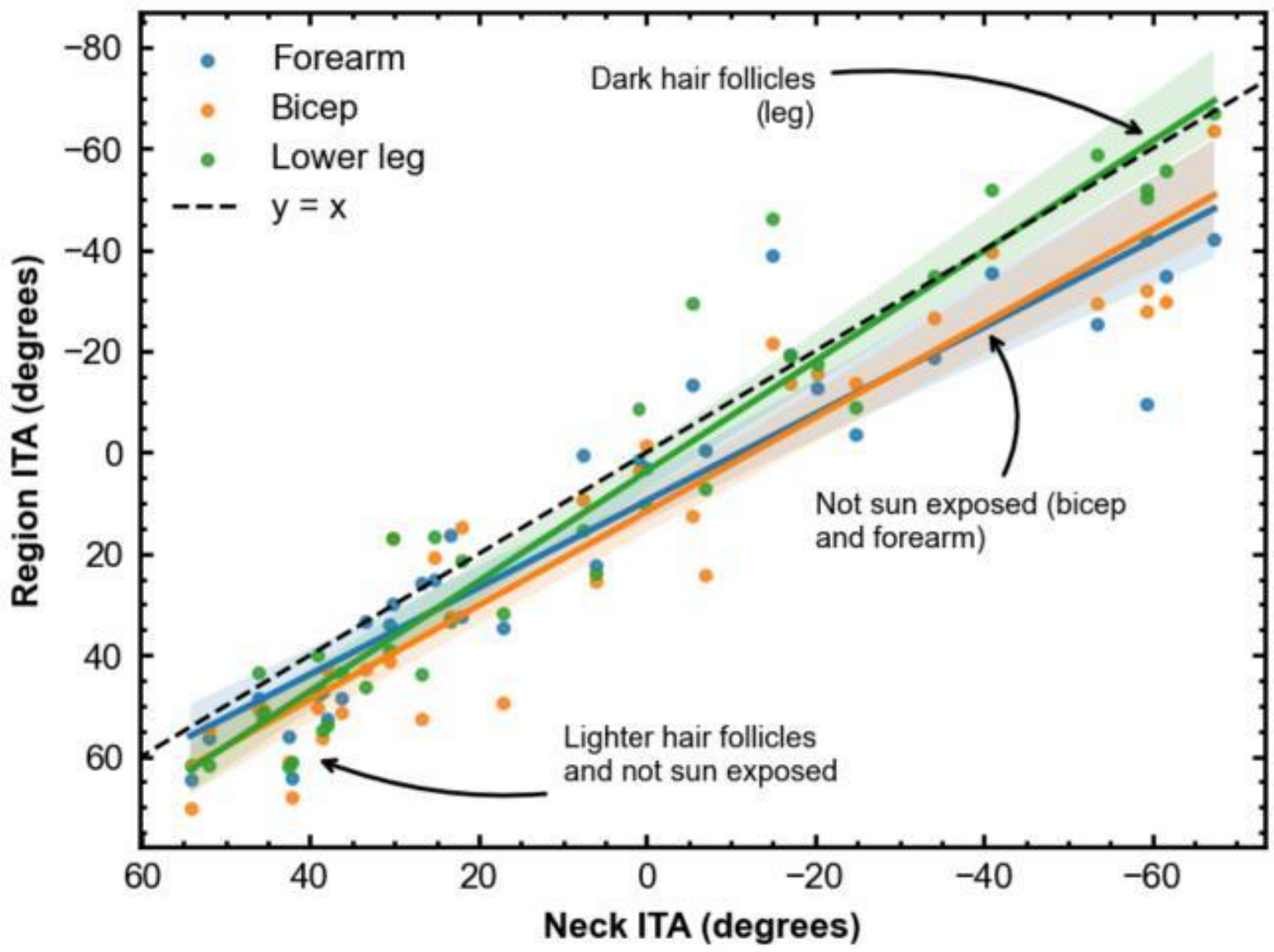

This is a very interesting question. There is a strong correlation between ITA in the neck, used as a sun-exposed region for reference, and the ITA measured in other regions – the forearm, bicep and lower leg (calf muscle)

Nonetheless, there are some interesting trends that can be observed. The gradient of the linear regression line of ITA between neck and the lower leg has a gradient statistically significantly different to 1 (p=0.005), likely because of the presence of hair follicles, which counteract differences in sun exposure between the two sites. The bicep and forearms do not have a statistically significant difference in their gradients from 1 (y = x line) but have a statistically significant offset in their ITA values from the neck (11 and 9 degrees higher respectively, p<0.001, p=0.004 respectively).

We find this analysis to be informative, so have added as Supplementary Figure 2 and included a discussion of the results above in the results section [Lines 135-150].

18. “The correction method also worked well for a matched region on a vitiligo participant and was able to match the sO2EST across their vitiligo and pigmented regions.” What were the ITA values for this patient?
    Non-pigmented region: 26.9 degrees
    Pigmented region: 1.1 degrees
    We have included this data in the relevant paragraph of the results section. [Lines 300-302]
19. “... we finally sought to develop a deeper understanding of the origin of the clutter artefacts observed. Initial qualitative evaluation suggested that the artefacts appeared in the PA image approximately twice as far below the skin surface as strongly reflecting structures in the ultrasound image, with spectral features similar to that of melanin in the skin surface (Fig. 1E, Figs S2 and S3). The consistent appearance suggested that the skin could be acting as a secondary source of acoustic waves due to the strong optical absorption, which were backscattered at interfaces between tissues of different acoustic impedance. Upon further interrogation, it was found that the backscattered signals were being erroneously reconstructed into the photoacoustic image based on the wave travel time (Fig. 6A).” I agree with everything stated, but this is all speculative, and much of this speculation has already been shown or reported in the literature in both non-invasive and minimally invasive photoacoustic applications. For example:
    a. Preisser, Stefan, et al. "Study of clutter origin in in-vivo epi-optoacoustic imaging of human forearms." Journal of Biomedical Optics 18.9 (2016): 094003.
    b. Jaeger, Michael, et al. "Deformation-compensated averaging for clutter reduction in epiphotoacoustic imaging in vivo." Journal of Biomedical Optics 17.6 (2012): 066007-066007.
    c. Petrosyan, Tigran, et al. "Rapid scanning wide-field clutter elimination in epi-optoacoustic imaging using comb LOVIT." Photoacoustics 10 (2018): 20-30
    d. Lediju Bell, Muyinatu A., et al. "In vivo visualization of prostate brachytherapy seeds with photoacoustic imaging," J. Biomed. Opt. 19(12) 126011 (22 December 2014)

    We agree that prior papers have included speculation that the melanin absorption in the epidermis leads to the observed clutter. In our study, we go further than the analyses in these references, providing substantial evidence to support the production of acoustic waves in the epidermis as the physical basis of the signal generation. We have used a plane wave ultrasound reconstruction to unequivocally demonstrate that the melanin pigmentation is

indeed the source of the clutter. We have updated our manuscript to ensure that prior studies are appropriately cited (e.g. lines 77-88, 174-1176, 222-223, 352-357, 421, 438-440) and to explicitly explain how we have pursued the source of the clutter, going beyond speculation to demonstrating physical origins (e.g. lines 110-112, 359-366, 398-400, 438-446).

Hence, the claims about this paper reporting these observations for the first time, with supporting theory, need to be removed (or toned down) in the abstract and introduction section of this paper. Examples of questionable statements in light of the above references:

We hope that the earlier clarification in response to points 1 and 2 highlight that these prior studies did not demonstrate the source of the ultrasound but speculated as to its origins. We will outline in response to the points below key findings that go beyond speculation.

e. Abstract: “Accompanied by detailed theoretical modelling, we demonstrated that strong light absorption by melanin leads to spectral colouring, which dominates in individuals with low skin melanin pigmentation” This statement is misleading. There is no theoretical modeling in the results. Instead, there is only speculation to support the presented results. A model is presented in the methods section, but that is not a model of the demonstration being reported. Also, references 9, 11, and others already show the impact of melanin on spectral coloring.

   This point is incorrect: our manuscript includes optical modelling of how light fluence changes as a function of melanosome volume fraction (provided in Fig. 3C, 5C and D), and acoustic modelling of how changes to initial pressure in the epidermis affect photoacoustic and mUS images (in Fig. 6 and Supplementary Figures 12, 13 and 14). We undertook the modelling as observations in reference 9 (Mantri et al.) are inconsistent with fluence effects. The optical modelling builds on our previous work in reference 11 (Else et al. 2023), and results are consistent with *in vivo* data underpinning the fluence correction model.

f. Abstract: “We further identified the backscattering of ultrasound waves generated in the skin as a major source of image artefacts for individuals with high skin melanin pigmentation.” As noted in comments #1 and #4, this has already been reported by Fernandes et al. Photoacoustics 2023 and in the additional supporting references listed above.

As noted in response to point 1, Fernandes et al indeed noted the correlation between the presence of clutter and increased skin pigmentation, but did not demonstrate the origin of the clutter, which is what we have highlighted in the abstract.

Intro: “We then undertook detailed theoretical modelling to improve understanding of both the optical and acoustic effects observed.” The model was not necessary to improve understanding. If the model is removed, existing literature would still support everything presented in the results section.

While we agree that existing literature would qualitatively support everything presented in the Results section, modelling was crucial to quantitatively explain the physical observations made in the Results section and derive a generalisable correction. Without modelling, there would be an unexplained discrepancy in oximetry results between this study and Mantri et al. We clarify the relative contribution of spectral colouring and acoustic backscattering on oximetry, which has not been done before. Furthermore, the optical model underpins the fluence correction approach.

In addition to my suggestion to remove (or tone down) the above statements, more of the original and preceding work on this topic needs to be referenced to retain historical accuracy.

We agree that it is important to cite and explain the respective strengths and limitations of the preceding work, and we have added further suitable references to the works suggested and highlighted more explicitly our unique contributions in the introduction. As suggested in point 20, we also have added more comparisons to these prior studies in the discussion.

20. “Remarkably, mUS images computed from 700 nm PA time series data resemble conventional ultrasound images in all imaging sites, even at only modest levels of melanin (Fig. 6B).” How does this algorithm compare to previously reported algorithms that have the same end goal? Please add comparisons to DCA, SLSC, and LOVIT, which are three algorithms previously reported to address clutter in photoacoustic images (see references in comment #19 for more details). A comparison of strengths and weaknesses of each of these algorithms (and of mUS) would help to situate the significance, improvements, and advances achieved with the presented work.

    We agree that it important to add this comparison to the manuscript and have added a comparison of our work to DCA, SLSC and LOVIT in the Discussion section [Lines 438-442]. DCA, SLSC and LOVIT were developed to remove the clutter from the image. It is therefore worth noting explicitly that the purpose of using the mUS approach in our study was to demonstrate the

origin of the clutter rather than to remove it from the image, which is what the other algorithms are aiming to do.

21. "and could perhaps even derive ITA directly from the photoacoustic image given how well correlated we demonstrated them to be..." This claim is not supported by the manuscript contents. It would also fail for darker skin tones, as noted above. Either remove this claim, show more evidence of this for all skin tones, or provide a fairer treatment of the limitations for darker skin tones.

We agree with the reviewer that further evidence should be provided to support this claim. We have now run further analysis, adding an additional Supplementary Figure and paragraph to the Results section (Supplementary Figure 11) [Lines 335-343]. As the reviewer suggested, this still unfortunately fails on darker skin tones, even though the correlation between photoacoustic signal in the skin surface and melanosome volume fraction is excellent, as the acoustic clutter artefacts increasingly dominates, which cannot be accounted for in the fluence model.

New supplementary figure:

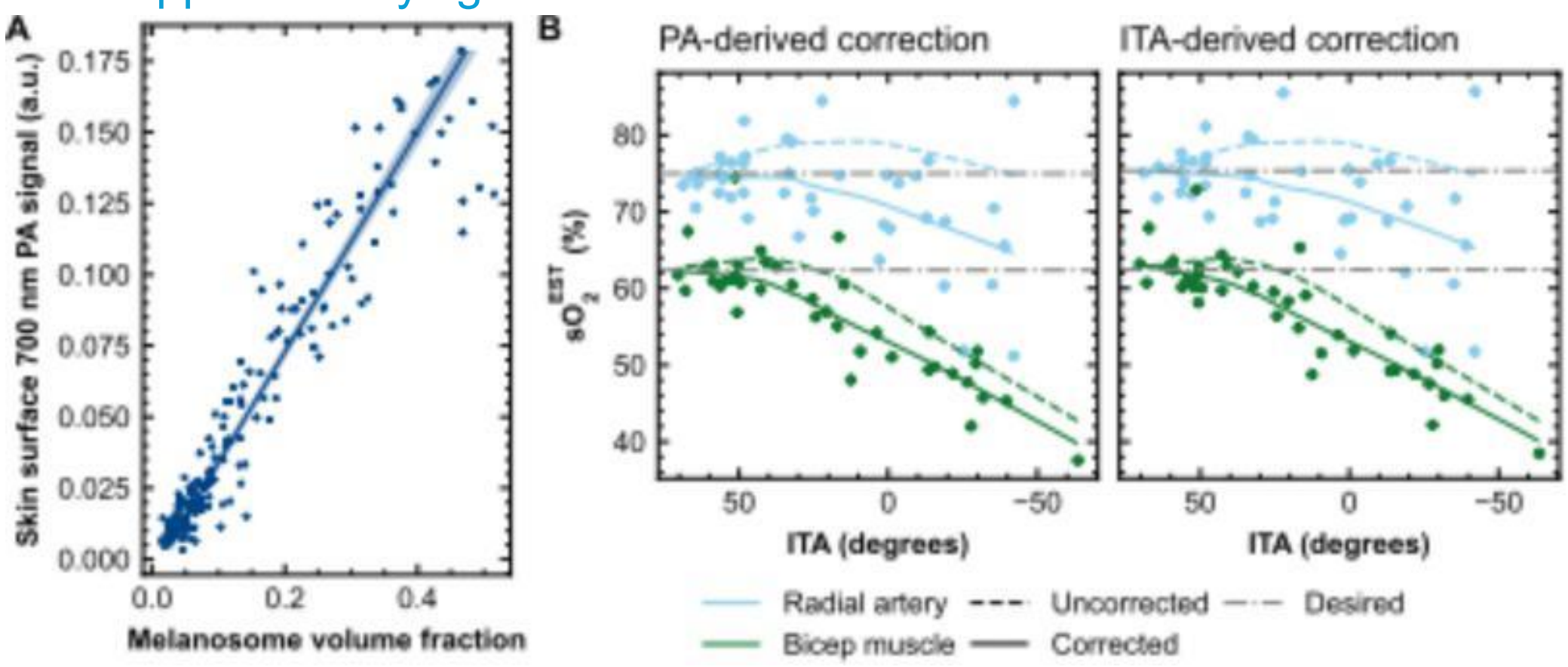

Please also see our response to point 5: the prediction of melanosome volume fraction from PA signal is still possible for darker skin tones, but the uncertainty appears to be a constant fraction of the signal rather than constant.

22. "knowing that the ultrasound reflectivity of the tissue can be obtained from photoacoustic time series data suggests that it could be incorporated into a model to separate out the primary photoacoustic signal from the scattered signals" This is another claim that is not supported by the manuscript contents. Please remove.

This comment is intentionally speculative, with "suggests" rather than "shows". Our study demonstrates that there are ultrasound images "hidden" in

photoacoustic data at low wavelengths in participants with darker skin tones. Given ongoing work by other groups in joint photoacoustic/ultrasound reconstruction, it is feasible that this sort of insight might provide additional information for a joint reconstruction approach, stimulating additional research in the field using the open data set that we are publishing alongside the paper.

23. Table 1 can be better formatted. For example, the standard deviations are not labeled as such and some spill into another row, while others remain on the same row as the +/- symbol. This formatting confusion exists for other table entries, too.

    We have transposed the table to provide more space.

Review of NCOMMS-25-56680A: "The confounding effects of skin colour in photoacoustic imaging" by Else et al.

Reviewer #1 (Remarks to the Author):

The authors have provided an extensive reply to author comments and have done an excellent job of responding and making meaningful changes to the manuscript. I believe that these modifications have significantly improved the quality of the manuscript. This is an impressive piece of work and is suitable for publication.

That said, please carefully review the references.

- Many Journal titles are not listed with using standard abbreviations.
- Titles of articles are inconsistently capitalized.
- Reference 31 does not actually provide a citation.
- In reference 39, the first author name appears to be omitted/incorrect.

Thank you for your positive assessment of the meaningful changes made to the manuscript, we agree that the quality of the manuscript has substantially improved and are delighted that you feel it is now suitable for publication.

We have now checked the references as requested and explicitly written the full journal title in all cases, capitalized titles consistently with the original article, and checked the references 31 and 39 as noted.

Reviewer #2 (Remarks to the Author):

This paper continues to report an important and timely topic. The authors have made important clarifications and distinctions in this revision. For example, the major conclusions presented in this paper are drawn from data that expands previous forearm results from volunteers, and some of the incorrect claims about what the authors did "first" or "identified" were removed or toned down. The inclusion of the bicep and neck regions positively bolsters findings relative to previous work. Despite these positive attributes, the group of vitiligo patients remains limited with none from the Fitzpatrick IV-VI groups, which would present greater variation of skin color for a single participant and would provide a more complete analysis of the claims made with respect to these patients. This is a noted weakness of the associated findings. Additional data from darker skin colors with vitiligo would help to strengthen this part of the paper, yet there is no mention of this weakness in the discussion section. The remaining contents of the paper present a novel and significant advance for the field, with some areas for improvement that do not require more data, as summarized below in three parts. Part I is concerned with major concerns based on the response to the reviewers. Part II is

concerned with details in the paper that are unclear. Part III summarizes a few minor details that need to be addressed, yet did not impact interpretation of the study results.

Thank you for your detailed feedback, we are encouraged by the positive assessment of the improvement of the manuscript compared to the prior version. The reviewer further raises several important points of clarification. We hope that the revisions provided here will help to clarify and improve our work.

Regarding the specific question of the limited range of skin types represented in the vitiligo cohort, we appreciate that this is a key limitation of this section of our study and have addressed this limitation in the discussion section (pgs. 11, 12).

Please note that we have now formatted the manuscript in LaTeX, so the page references here refer to the "di;" pdf file.

Part I: Major concerns based on the response to the reviewers

1. In the response to question 2 from reviewer 2, the authors state "while clutter has been explored previously, ***the source of clutter has not been unequivocally demonstrated." This is not a true statement.*** Fernandes *et al.* IUS 2023 conducted a series of multidomain photoacoustic simulations using five optical wavelengths, 18 skin constitutive pigmentations, and a previously validated 3D breast model. The abstract of said paper states: "Three scenarios were considered: (i) the baseline scenario, in which a photoacoustic target was surrounded by breast tissue and covered by a layer of skin; (ii) the baseline scenario with the skin layer removed; and (iii) the baseline scenario with the photoacoustic target removed. Clutter levels ranged -11 dB to 11 dB with the baseline scenario. **When skin was removed, this clutter was reduced by up to 39 dB and the associated target contrast improved by up to 39 dB relative to the baseline scenario, confirming skin as the primary source of clutter**." While the authors sought to demonstrate the source of clutter through multiple additional methods, this does not invalidate previous simulation demonstrations of the same conclusion. Hence, this relevant literature should be cited in statements such as the following: "The consistent appearance aligned with prior evidence that the epidermis could be acting as a secondary source of acoustic waves due to the strong optical absorption of melanin, which were backscattered at interfaces between tissues of different acoustic impedance[15,16,34,35]." I suggest that the authors revisit the paper cited above and assess the appropriateness of acknowledging it with a citation. From my perspective, the IUS 2023 paper by Fernandes *et al.* (doi:10.1109/IUS51837.2023.10307088) is directly relevant to a major claim of the paper under review, yet said paper was inexplicably omitted from the references.

- We are happy to cite this conference abstract. We would clarify our response to Q2 from reviewer 2 to say, "While clutter has been explored previously, the source of clutter has not been unequivocally demonstrated **in human subjects,**" which we maintain is a true statement.

2. In the response to question 4 from reviewer 2, the authors state "While Fernandes et al demonstrate a correlation between ITA and clutter level, they do not directly show that this is caused by acoustic reflections, as we have done here." In the response to question 19 from reviewer 2, the authors state "We hope that the earlier clarification in response to points 1 and 2 highlight that these prior studies did not demonstrate the source of the ultrasound but speculated as to its origins." ***These statements and others like it are not true*** for the reasons stated in point #1 above and demonstrated in 10.1109/IUS51837.2023.10307088.

- We maintain that previous **in human** studies were speculating as to the origin of the acoustic clutter. We appreciate that this has been demonstrated **in simulation** in a limited number of scenarios in Fernandes' IUS conference abstract of 2023 and have cited this.

3. In the response to questions 11 and 12 from reviewer 2, the authors state "This is true, but interestingly, when we responded to Reviewer 1's point 13, we calculated the corresponding plot to Figure 2C that included negatively valued pixels and the corresponding plot for the contrast to noise ratio. In this new analysis, we do see a decrease in photoacoustic signal with increasing melanin level, as predicted by optical considerations. We think this happens because the clutter signal is oscillatory (Figure 2B), cancelling out when negative pixels are included, but contributing positively when negative pixels are excluded. The negative component of the clutter labelled on the right above would be excluded under the original analysis, while the positive clutter peak would be included, compensating for the fluence-induced reduction in signal intensity when negative pixels are included." This is interesting, but the standalone statement in the paper about this finding is incorrect. In particular, the statement: "However, the mean signal intensity and corresponding contrast to noise ratio in the radial artery decreased significantly with increasing melanin level when negative pixels were included (Fig. S7)." is not true for the 1080 nm wavelength. While the following sentences try to address this, more attention should be given to ensure that the quoted sentence about Fig. S7 is accurate by adding appropriate nuance and caveats to the standalone sentence.

- We agree that this sentence does not make sense on its own, so have clarified that this refers to shorter wavelengths. We have also moved the sentence that discusses the wavelength dependence forward (pgs. 5-6).

4. Further to point #3 above, it is not clear why the observed effect about oscillatory clutter is wavelength dependent. It would help if the authors can clarify their proposed theory with respect to this detail.

- The oscillatory clutter corresponds to ultrasound backscatter from the plane wave generated in the epidermis. The backscatter off structures between the epidermis and the radial artery will be superimposed on the primary photoacoustic signal (from optical absorption) in the radial artery. The reconstructed photoacoustic signal in the radial artery, therefore, is a linear combination of the ultrasound back scatter and the primary photoacoustic signal.
- The mean amplitude of the pressure wave generated in the epidermis over time is zero (the wave should be approximately the derivative of the delta function), while the peak intensity is proportional to the initial pressure in the epidermis (increases with increasing melanin concentration and at shorter wavelengths where melanin absorbs more strongly).
- We therefore expect the photoacoustic signal in the radial artery to correspond to a linear combination of oscillatory clutter, with zero mean (from backscatter) and initial pressure from haemoglobin absorption.
- Hence, when the wavelength is shorter, there is more melanin absorption in the epidermis, causing more oscillatory signal.
- Therefore, our proposed explanation for this effect is that when negative pixels are included in the average, the oscillatory component mostly cancels, leaving primarily spectral colouring effects, i.e. a decrease in PA signal with increasing skin pigmentation.
- When negative pixels are excluded, as is typically done in the field to reduce artefacts of the image reconstruction process, the oscillatory component does not completely cancel, either cancelling out the spectral colouring effect as in the radial artery or causing the PA signal to increase with increasing skin pigmentation as in the bicep data. That is now clarified in the manuscript.

5. In response to question 5 from reviewer 2, the authors state, “The increased variability appears to be due to a constant percentage variability in the PA signal in the skin surface, which can be seen when plotted on a log scale. Here all imaging sites are included and we fitted a LOWESS smooth fit with bootstrapping to calculate the confidence interval.” However, I could not find these details included in the paper. The authors also seem to argue that a high number of patients is more important than addressing the observed differences with the darker skin tones. Note that these are two separate issues (i.e., statistical significance for all participants vs. results that are relevant to one subgroup and not another subgroup), and they should not be pitted against each other. The

associated questions were not concerned with statistical significance or the total number of patients.

- We have included this data in Supplementary Figure 1 and refer to this on pg. 5.
- We don't mean to imply that a higher number of patients is more important than addressing the observed differences with darker skin tones. Addressing the observed differences for darker skin tones is absolutely critical.
- We have included more of a discussion of the limitations of our study, particularly regarding the vitiligo cohort in the Discussion section.

6. The response to question 21 from reviewer 2 is insightful to address some of the subgroup concerns that were raised, but the negative result for darker skin tones is not clearly presented in the paper.

- We recognise that we could more carefully describe the limitations of our study, including the negative result for darker skin tones. We have addressed this in response to point 13 below.

7. In the introduction section, the authors reference conflicting results between references 9 and 11. What exactly is the conflict being referred to?

- Thank you for flagging this, this is an important point of clarification. We refer to the discrepancy in the trend of sO_2 prediction with increasing skin pigmentation. Mantri et al. observed decreasing sO_2 with increasing skin pigmentation, while our previous optical modelling suggested the opposite trend.

8. "For individuals with very high skin pigmentation, image clutter is shown to originate from the skin surface acting as an ultrasound transducer, with photoacoustic waves generated in the epidermis being backscattered throughout the image field." This may simply be an oversight, but it's important to mention again that this was already shown by previous investigators. By not citing previous work, I am left with the incorrect impression that the authors are reporting this particular finding for the first time. See Part I comment 1 for more details. The authors presented several points to explain why their study differs from previous studies, but this critical concern remains unaddressed.

- While we recognise the contribution made by Fernandes et al 2023 (IUS) in simulation, we maintain that this is a key result of our work, which has not been unequivocally demonstrated **in human subjects** before.
- To address key papers directly that the reviewer may be referring to:
    - Fernandes et al 2023 (photoacoustics): while they state that "**It is expected** that PA images from darker skinned individuals will suffer from both lower light fluence delivered within the imaging plane and higher levels of clutter artifacts" and show that clutter increases as ITA decreases, they do not show that this is from acoustic backscattering.
    - Fernandes et al 2023 (IUS abstract) conducted simulations rather than studies in human subjects.

- Preisser et al. 2016 (IoP Journal of Optics) conducted a study with ink painted onto a subjects' forearm, not in subjects with different skin tones.

9. "All participants underwent photoacoustic imaging (PAI) with simultaneous reflection ultrasound (US) computed tomography and colourimetry at all each imaging site." It seems that "all" needs to be removed or otherwise its purpose should be clarified. There are at least 1-2 other typos in the newly added red text, which did not affect overall interpretation.

- Thank you for bringing these to our attention, we have now corrected these errors.

10. "The regions of interest analysed showed some variation in size and depth (mean ± 164 standard deviation): radial artery diameter 8.5 ± 1.0 mm, depth 2.9 ± 0.8 mm; carotid artery diameter 10 ± 4 mm, depth 15 ± 3 mm. The centroid of the bicep muscle region was at a depth of 14.2 ± 1.4 mm, while that of the calf muscle was 14.3 ± 1.6 mm. Muscle regions of interest included as much of the muscle as was visible in the field of view." This text seems out of place in the results subsection labeled "A diverse healthy volunteer study was vital to understand confounding effects of skin colour in photoacoustic imaging." These are not details that contribute to the subheading and are instead details about the methodology, which would be better placed in the Methods & Materials section to avoid confusion about the main message in this section. If the text stays, a concluding statement about why variations in size and depth affect the understanding of confounding effects needs to be added to the quoted paragraph. The conclusion seems to be that "there was no systematic relationship between ITA and region of interest depth" based on the response to Reviewer 1 comment 11, but no data is presented to support this conclusion.

- We have moved this to the methods section (pg. 14).
- Here is the analysis of the target depth vs. ITA. We do not feel it is helpful to include in the paper, but we have quoted statistical significance levels (pg. 14).

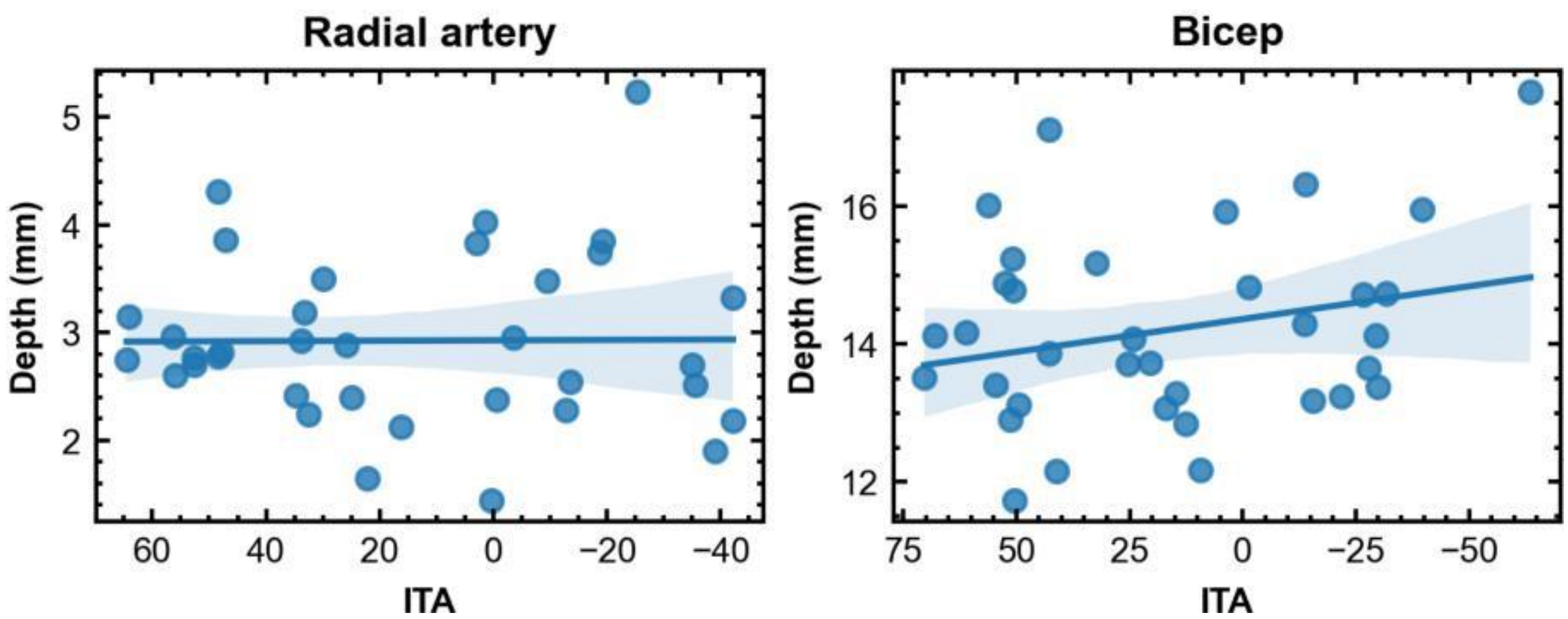

-

11. It is not clear if the regions of interest noted above refer to the CNR measurements in addition to the measurements taken to assess signal trends as a function of skin tone. Please clarify.

- We used the radial artery region of interest as a target for calculating the CNR and constructed a region at the same depth with the same area 2 cm to the left as the noise region. We have clarified this point in the methods section (pg. 14).

12. If the regions of interest quoted in point #10 were indeed used to calculate the CNR, for the muscles, it is not clear if the statement "Muscle regions of interest included as much of the muscle as was visible in the field of view." refers to the identified target or background region of interest. In addition, the sizes of the background regions of interest when calculating the CNR of the arteries are not specified. More details are needed to better understand the authors' approach to this calculation.

- We did not present the contrast-to-noise ratio for the muscle region of interest.
- We have included further detail regarding the calculation of CNR for the radial artery region of interest on pgs. 14.

13. "Unfortunately, this did not compensate for biases observed for darker skin tones, suggesting that spectral colouring becomes a less significant source of bias as melanin levels increase." It is not clear what features of the data presented in Fig. 5 support this conclusion.

- Thank you for giving us the opportunity to clarify this point. We did not mean to say that spectral colouring is less significant as melanin levels increase – indeed it would presumably be far more significant – but rather that it ceases to be the dominant effect as acoustic scattering comes to dominate.
- We have updated the text to say "Unfortunately, this did not compensate for biases observed at higher melanin levels, suggesting that spectral colouring cannot explain the observed trends alone," alluding to results in the next section which highlight the key role of acoustic backscattering.

14. "Given the strong correlation between ITA and photoacoustic signal (Fig. 1G), we derived a fluence correction factor based purely on the mean photoacoustic signal from the skin surface." When structures from lower ITA values are not visible because of optical penetration issues, it is unlikely that this approach will be sufficient. Perhaps a less than (<) sign missing in the statement "removing a linear ITA dependence for ITA > 10 ($p = 0.63$) and ITA > 30 ($p = 0.85$) respectively." If not, why is the linear dependence removed for both ITA > 10 and ITA > 30? Is this referring to the pre- and post-correction cases discussed in the preceding paragraph? Further, the authors reported Fig. S4, which presents a statistical significance in these ITA ranges, so the methodology and reported p value discrepancies are unclear here.

- We agree that our approach to correct for spectral colouring will break down at lower ITA levels due to the overwhelming effect of acoustic back scattering, as seen in Figure 5D and Fig. S11, and acknowledge this in the manuscript.
- The removal of the linear dependence for ITA > 10 (in the radial artery) and ITA > 30 (in the bicep) respectively refers to the effectiveness of the proposed correction algorithm over those ranges, as in the paragraph before this.
- We hope that the edits we have made clarify this (pg. 9).

15. "To address the acoustic effects, we modelled the epidermis as a source of ultrasound plane waves, allowing us to reconstruct ultrasound images from the clutter signal." The advantage of doing this is unclear. I think the more appropriate message is that the mUS images were made to demonstrate that skin is the source of clutter, but this does not come across clearly based on the way the sentence is written, as the acoustic effects were not "addressed", just demonstrated. In other words, the technique to reconstruct mUS images does not "address" the source of the clutter in the same way that the optical effects were addressed in the preceding sentence. Consider rewriting this sentence to clarify the intent here.

- We have changed our use of the word "address" in this context to "better understand" (pg. 11).

16. "Because of the high optical absorption of melanin, the epidermis acts as an ultrasound transmitter, emitting waves into the deeper tissue, which give rise to reflected ultrasound signals that are far stronger than the desired primary photoacoustic signal from the tissue of interest." A citation to prior references that state the same is warranted here, as this is not a new idea.

- We have added citations to the relevant literature here (pg. 11).

17. This is not true of DCA (reference 16) or PAFUSion (reference 12): "but require either specialist hardware to cancel the scattered wave or introduce a non-linear scaling to the intensity making quantification impossible." To be accurate about this, I suggest (a) adding "unique acquisition protocols" to this list and (b) including the appropriate citation(s) next to each limitation, rather than at the end of the sentence. Without suggestion b, it is not clear which limitation the authors are claiming to be associated with each technique cited. Further, note that PAFUSion is primarily concerned with reflection artifacts from highly reflective surrounding structures, which is not the same as the scattering events that cause the acoustic clutter. Therefore, this algorithm is not really relevant here, and my above suggestion does not correct the inaccurate limitation(s) listed for this technique.

- We have updated the text to be clear about which limitation refers to which study (pg. 11).

18. “Photoacoustic signals from the skin surface region at the lowest wavelength of 700 nm (highest melanin absorption) increased only modestly between non-pigmented regions and pigmented regions, possibly because of absorption from other chromophores like haemoglobin...” Another possibility is that the group of vitiligo patients contains none from the Fitzpatrick IV-VI groups. This should be mentioned more prominently if the authors agree with this probability as stated in the response to the reviewers.

- We agree and have updated the text to more prominently highlight the limited range of skin types in the vitiligo cohort (pg. 8).

19. The group of vitiligo patients remains limited with none from the Fitzpatrick IV-VI groups, which would present greater variation of skin color for a single participant and would provide a more complete analysis of the claims made with respect to these patients. Why is there no mention of this weakness in the discussion section? An appropriate place to discuss this would be where the following text exists: “Additional variation in skin surface pigmentation, for example, increased pigmentation associated with scar tissue or darker areolar skin in breast imaging, or decreased pigmentation with regression in melanoma imaging, could compound these effects.” The extension to scar tissue and breast tissue variations here appears as an unnecessary diversion from the critical detail about skin color variations in vitiligo patients with darker skin tones, which were not included in this study. Please prioritize discussion of this important limitation. This should take priority over the apparent diversion of readers’ attention to speculative information about scar tissue and breast tissue applications that cannot be verified.

- We agree that a more focussed concluding paragraph would help to ensure that the reader is not diverted. We have removed the speculation about scar tissue and areolar tissue from the final paragraph and moved it to a dedicated paragraph about the limitations of the study. (pgs. 11/12)
- We have further elaborated on the limitations of our correction method, and about the lack of vitiligo patients. (pgs. 11/12).

20. The speculative details about scar tissue, breast tissue, etc. come across as a disconnected distraction from the key results presented in this paper about a very different patient population. Consider this perspective and how to best address this concern, either through removal of said text or through updated text that better justifies its inclusion. Multiple unsupported speculations should really be avoided. Although the authors think this could stimulate future research (as noted in response to Reviewer 2 comment 22), such speculations can also perpetuate inaccuracies if they are not true. Ideally, there would be some initial result demonstrated before such speculations are made in archived literature.

- We agree that this was somewhat disconnected given the key message in the final paragraph of our paper. We have now concluded this in a more detailed

paragraph encompassing the limitations of our study regarding recruitment and the applicability of our proposed correction approach. (pg. 12)

21. References 14 and 34 are duplicates of the same paper.

- We have fixed this, thank you for pointing this out.

22. There is insufficient information to locate Reference 31, which only states "Fitzpatrick skin phototype."

- Thank you for pointing this out. We have updated this reference.

23. A reference to the Monk scale is similarly needed.

- Thank you for flagging this, we have added a reference.

24. In response to Reviewer 1 question 8, the authors state: "While addressing this question, we uncovered a single outlier in the ITA measurements of participant 25 (radial artery) by plotting the standard deviation of the five measurements against the mean (see figure below)." However, the referenced figure states the mean was taken over three rather than five measurements. This is confusing.

- Thank you for pointing this out. This is a typo that was also present in the paper. We have corrected this to say 5.

25. "The overall signal intensity of different structures did not decrease with increasing skin pigmentation, as might be naively predicted..." Remove "naively". It is not scientifically appropriate to qualify the associated thought this way, and the sentence retains its meaning without this word.

- We have removed the word naively.

26. "In the bicep, there was a dramatic increase in mean photoacoustic signal with increasing skin pigmentation..." There is a lot of variation in the results presented in Fig. 2D. Given these variations, it is unclear how the reported trends are statistically significant. It is also concerning that the standard deviations in Fig. 2D are larger than some of the mean values in Fig. 2D.

- While there is a high level of variation, we carried out a standard linear regression analysis and did not remove any of the apparent outliers. Despite the variance, for the bicep muscle in Fig. 2D (right panel), there is a clear trend consistent with the corresponding statistical significance levels. I ran the analysis with bootstrapping to account for any non-normally distributed values, which gave the same result.
- This is an equivalent of Figure 2D (bicep) with 95% confidence intervals for the linear regression line. Gradient>1 is clearly within the confidence interval for 700 nm and 800 nm but not 1080 nm, consistent with normal linear regression calculations shown in the paper.

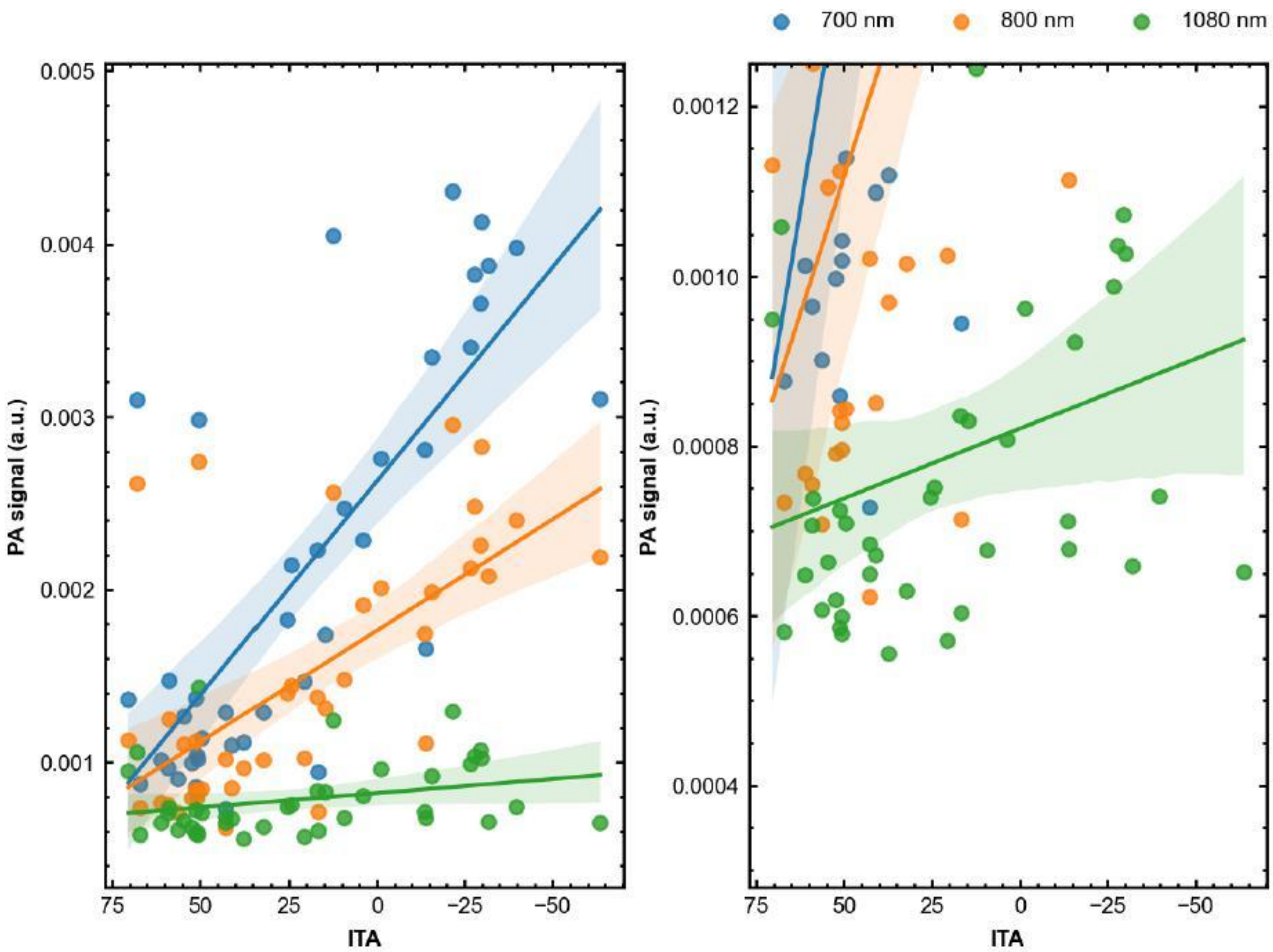
700 nm
800 nm
1080 nm
PA signal (a.u.)
ITA
0.001
0.002
0.003
0.004
0.005
0.0004
0.0006
0.0008
0.0010
0.0012
75
50
25
0
−25
−50

Review of **"The confounding effects of skin colour in photoacoustic imaging"** by Else et al.

This paper reports a timely and relevant topic. Much more research is needed to shed light on the asymmetrical impact of skin tone on photoacoustic imaging system performance. I commend the authors for tackling this problem. The most interesting aspects of this work are the performance observed in muscle data, vitiligo patients, and the method introduced to address the noted problems. Other aspects are not new and can be deemphasized to avoid overstating the importance, novelty, and significance of this work, which will otherwise call into question the entire body of work. This is a major concern and noted weakness of the presentation, as described in more detail below.

1. Abstract: "We further identified the backscattering of ultrasound waves generated in the skin as a major source of image artefacts for individuals with high skin melanin pigmentation." This is not a new finding and it has been demonstrated before. See for example the introduction section of the work by reference 8 (Fernandes et al. Photoacoustics 2023). The claim here needs to be toned down.
2. Introduction: "a comprehensive assessment of the confounding effects of skin colour has yet to be performed for PAI." There are multiple papers that show the confounding effects of skin color. For example, references 8, 9, 10, 11, and:
    - Fernandes, Guilherme S. Pilotto, et al. "Impact of skin pigmentation on photoacoustic imaging using linear array transducer: a pilot in vivo study." *2022 IEEE International Ultrasonics Symposium (IUS)*. IEEE, 2022.
    - Fernandes, Guilherme SP, et al. "In silico demonstrations of the impact of wavelength and skin tone on photoacoustic breast imaging." *2023 IEEE International Ultrasonics Symposium (IUS)*. IEEE, 2023.

    Could the authors clarify what about all of this work is not considered comprehensive? This will help to maintain historical accuracy and to ensure proper claims are being made.

3. "Here, we present the first comprehensive assessment of the confounding effects of skin tone in PAI." This is not a true statement. See comment #2 for details, discuss the noted references, and state what is actually "first" given this existing literature. Any of the papers noted in comment #2 can be considered among the first comprehensive assessments of the confounding effects of skin tone in PAI as far as I am aware.
4. "For individuals with very high skin pigmentation, image clutter is shown to originate from the skin acting as an ultrasound transducer, with photoacoustic waves generated in the skin being backscattered throughout the image field." This was already reported in the work by Fernandes et al. Photoacoustics 2023 (see quote from this paper below):
    - "First, the illumination beam strikes the skin, which can be characterized by high optical absorption coefficient in the visible and near-infrared range, due to the melanin content in the epidermis layer [17,18]. This light absorption can considerably affect the illumination efficiency, particularly for deep-located structures in the imaging plane. Second, the PA waves generated due to light absorption at the skin surface can cause the skin to act as an US emitter, transmitting sound waves beyond the skin surface, which are then reflected by

> typical US scatterers within tissue, thus creating considerable artifacts in PA images [19]. In particular, PA waves generated by strong optical absorbers at the skin surface interact with the surrounding media and travel back to the US transducer, introducing an artifact known as acoustic clutter."

Hence, this paper needs to be cited at the end of the quoted statement in the paper under review. And, the claim can perhaps be that the authors are providing additional evidence of this. However, the evidence is questionable, as noted above with more details provided later in this review.

5. "... suggesting that the Fitzpatrick scale was a suitable proxy for skin colour." This is not true for the darker skin tones. The Fitzpatrick scale lumps multiple dark skin tones into a single value. This is not a suitable proxy for darker skin tones, and this limitation needs to be more clearly acknowledged. This major concern is supported by the higher ITA standard deviations in Table 1 and max/min values in Fig. 1E with the darker skin tones. Similarly, the darker skin tone results presented in Fig. 1G further supports this, as these data points do not follow the linear fit. What is the result when they are fitted separately?
6. What do the circles in Fig. 1E represent?
7. "We confirmed the relationship between photoacoustic signal from the skin surface and skin pigmentation quantified by colorimetry." Fernandes et al. Photoacoustics 2023 already showed this, and this should be acknowledged. See Fig. 3 from this paper.

[FIGURE REDACTED]

8. “In forearm scans, the radial artery was clearly distinguished at long wavelengths...” Which wavelengths are the authors considering “long”? Please specify specific wavelengths in this sentence.
9. In general, the findings about the radial artery in this paragraph were previously shown by Fernandes et al. Photoacoustics 2023. For example, “Conversely, it was entirely obscured by clutter (very dark or very light arc-shaped features below the skin surface in the images) in participants with darker skin, particularly at shorter wavelengths (Fig. 2A).” This has been shown and is not a novel finding. It would be more accurate to report as a confirmation of previously reported results.
10. “Our qualitative assessment suggested that the forearm was the most informative site for understanding the confounding effects of skin colour due the presence of the radial artery as a focal feature, along with background tissue and bone.” Considering that the majority of the paper is based on the radial artery results, the novelty and significance of the entire paper (relative to the work by Fernandes et al. Photoacoustics 2023) is called into question. I recommend de-emphasizing results that have already been shown and instead focus on emphasizing what is truly new in this work.
11. “Perhaps surprisingly, the overall signal intensity of different structures did not decrease with increasing skin pigmentation, as might be naively predicted.” This is not surprising based on the figure below (Fig. 5), taken from Fernandes et al. Photoacoustics 2023:

[FIGURE REDACTED]

Part (d) of this figure shows no statistically significant differences in the radial artery (RA) signal as a function of skin tone. There is no need to make a naive prediction about this given this supporting data.

12. “in the radial artery, there was a negligible change in signal at several wavelengths used for blood vessel imaging in commercial devices” This was also shown in part (d) of the figure below (Fig. 6), taken from Fernandes et al. Photoacoustics 2023:

[FIGURE REDACTED]

13. “The standard deviation of the signal across the radial artery increased significantly at 700 nm, in line with the observations of increased clutter...” I do not understand the comparison being made here. Increased significantly relative to what? And, why is this reflective of increased clutter. More details are needed to more clearly convey this thought.
14. “All participants in the vitiligo cohort would otherwise have been placed in Fitzpatrick types I, II, or III...” Patients with Fitzpatrick types IV, V, and VI will suffer the most from the effects being reported. It would be more impactful to show how the reported methods alleviate this concern in patient cohorts with vitiligo and Fitzpatrick types IV, V, and VI. Could the authors recruit more patients to show this impact?
15. In Table 1 and S1, the multiple ITA values measured on the patients with vitiligo should be reported. It is not clear what patch of color is being reported in Table 1 or S1. Why is there only one ITA value in Table S1, when these patients have at least two ITA values for different patches of skin?
16. “Photoacoustic signals from the skin surface region at the lowest wavelength of 700 nm (highest melanin absorption) increased only modestly between non-pigmented regions and pigmented regions, likely because of absorption from other chromophores like haemoglobin ($p = 0.059$, linear mixed effects model; Fig. 4D).” Is this expected to be true for lower ITA values? I am doubtful about this. More evidence is needed to support this claim for lower ITA values.

17. Is the correlation between the ITA value of one patch color vs. another and the underlying photoacoustic signals similar or different as ITA values decrease?
18. “The correction method also worked well for a matched region on a vitiligo participant and was able to match the sO_2^{EST} across their vitiligo and pigmented regions.” What were the ITA values for this patient?
19. “... we finally sought to develop a deeper understanding of the origin of the clutter artefacts observed. Initial qualitative evaluation suggested that the artefacts appeared in the PA image approximately twice as far below the skin surface as strongly reflecting structures in the ultrasound image, with spectral features similar to that of melanin in the skin surface (Fig. 1E, Figs S2 and S3). The consistent appearance suggested that the skin could be acting as a secondary source of acoustic waves due to the strong optical absorption, which were backscattered at interfaces between tissues of different acoustic impedance. Upon further interrogation, it was found that the backscattered signals were being erroneously reconstructed into the photoacoustic image based on the wave travel time (Fig. 6A).” I agree with everything stated, but this is all speculative, and much of this speculation has already been shown or reported in the literature in both non-invasive and minimally invasive photoacoustic applications. For example:
    - Preisser, Stefan, et al. "Study of clutter origin in in-vivo epi-optoacoustic imaging of human forearms." *Journal of Biomedical Optics* 18.9 (2016): 094003.
    - Jaeger, Michael, et al. "Deformation-compensated averaging for clutter reduction in epiphotoacoustic imaging in vivo." *Journal of Biomedical Optics* 17.6 (2012): 066007-066007.
    - Petrosyan, Tigran, et al. "Rapid scanning wide-field clutter elimination in epi-optoacoustic imaging using comb LOVIT." *Photoacoustics* 10 (2018): 20-30
    - Lediju Bell, Muyinatu A., et al. "In vivo visualization of prostate brachytherapy seeds with photoacoustic imaging," J. Biomed. Opt. 19(12) 126011 (22 December 2014)

    Hence, the claims about this paper reporting these observations for the first time, with supporting theory, need to be removed (or toned down) in the abstract and introduction section of this paper. Examples of questionable statements in light of the above references:

    - Abstract: “Accompanied by detailed theoretical modelling, we demonstrated that strong light absorption by melanin leads to spectral colouring, which dominates in individuals with low skin melanin pigmentation” This statement is misleading. There is no theoretical modeling in the results. Instead, there is only speculation to support the presented results. A model is presented in the methods section, but that is not a model of the demonstration being reported. Also, references 9, 11, and others already show the impact of melanin on spectral coloring.
    - Abstract: “We further identified the backscattering of ultrasound waves generated in the skin as a major source of image artefacts for individuals with high skin melanin pigmentation.” As noted in comments #1 and #4, this has already been reported by Fernandes et al. Photoacoustics 2023 and in the additional supporting references listed above.

- Intro: “We then undertook detailed theoretical modelling to improve understanding of both the optical and acoustic effects observed.” The model was not necessary to improve understanding. If the model is removed, existing literature would still support everything presented in the results section.

In addition to my suggestion to remove (or tone down) the above statements, more of the original and preceding work on this topic needs to be referenced to retain historical accuracy.

20. “Remarkably, mUS images computed from 700 nm PA time series data resemble conventional ultrasound images in all imaging sites, even at only modest levels of melanin (Fig. 6B).” How does this algorithm compare to previously reported algorithms that have the same end goal? Please add comparisons to DCA, SLSC, and LOVIT, which are three algorithms previously reported to address clutter in photoacoustic images (see references in comment #19 for more details). A comparison of strengths and weaknesses of each of these algorithms (and of mUS) would help to situate the significance, improvements, and advances achieved with the presented work.
21. “and could perhaps even derive ITA directly from the photoacoustic image given how well correlated we demonstrated them to be...” This claim is not supported by the manuscript contents. It would also fail for darker skin tones, as noted above. Either remove this claim, show more evidence of this for all skin tones, or provide a fairer treatment of the limitations for darker skin tones.
22. “knowing that the ultrasound reflectivity of the tissue can be obtained from photoacoustic time series data suggests that it could be incorporated into a model to separate out the primary photoacoustic signal from the scattered signals” This is another claim that is not supported by the manuscript contents. Please remove.
23. Table 1 can be better formatted. For example, the standard deviations are not labeled as such and some spill into another row, while others remain on the same row as the +/- symbol. This formatting confusion exists for other table entries, too.

Review of NCOMMS-25-56680A: "The confounding effects of skin colour in photoacoustic imaging" by Else et al.

This paper continues to report an important and timely topic. The authors have made important clarifications and distinctions in this revision. For example, the major conclusions presented in this paper are drawn from data that expands previous forearm results from volunteers, and some of the incorrect claims about what the authors did "first" or "identified" were removed or toned down. The inclusion of the bicep and neck regions positively bolsters findings relative to previous work. Despite these positive attributes, the group of vitiligo patients remains limited with none from the Fitzpatrick IV-VI groups, which would present greater variation of skin color for a single participant and would provide a more complete analysis of the claims made with respect to these patients. This is a noted weakness of the associated findings. Additional data from darker skin colors with vitiligo would help to strengthen this part of the paper, yet there is no mention of this weakness in the discussion section. The remaining contents of the paper present a novel and significant advance for the field, with some areas for improvement that do not require more data, as summarized below in three parts. Part I is concerned with major concerns based on the response to the reviewers. Part II is concerned with details in the paper that are unclear. Part III summarizes a few minor details that need to be addressed, yet did not impact interpretation of the study results.

Part I: Major concerns based on the response to the reviewers

1. In the response to question 2 from reviewer 2, the authors state "while clutter has been explored previously, ***the source of clutter has not been unequivocally demonstrated."*** ***<u>This is not a true statement</u>.*** Fernandes *et al.* IUS 2023 conducted a series of multidomain photoacoustic simulations using five optical wavelengths, 18 skin constitutive pigmentations, and a previously validated 3D breast model. The abstract of said paper states: "Three scenarios were considered: (i) the baseline scenario, in which a photoacoustic target was surrounded by breast tissue and covered by a layer of skin; (ii) the baseline scenario with the skin layer removed; and (iii) the baseline scenario with the photoacoustic target removed. Clutter levels ranged -11 dB to 11 dB with the baseline scenario. **When skin was removed, this clutter was reduced by up to 39 dB and the associated target contrast improved by up to 39 dB relative to the baseline scenario, confirming skin as the primary source of clutter**." While the authors sought to demonstrate the source of clutter through multiple additional methods, this does not invalidate previous simulation demonstrations of the same conclusion. Hence, this relevant literature should be cited in statements such as the following: "The consistent appearance aligned with prior evidence that the epidermis could be acting as a secondary source of acoustic waves due to the strong optical absorption of melanin, which were backscattered at interfaces between tissues of different acoustic impedance[15,16,34,35]." I suggest that the authors revisit the paper cited above and assess the appropriateness of acknowledging it with a citation. From my perspective, the IUS 2023 paper by Fernandes *et al.* (doi: 10.1109/IUS51837.2023.10307088) is directly relevant to a major claim of the paper under review, yet said paper was inexplicably omitted from the references.
2. In the response to question 4 from reviewer 2, the authors state "While Fernandes et al demonstrate a correlation between ITA and clutter level, they do not directly show that this is caused by acoustic reflections, as we have done here." In the response to question 19

from reviewer 2, the authors state "We hope that the earlier clarification in response to points 1 and 2 highlight that these prior studies did not demonstrate the source of the ultrasound but speculated as to its origins." ***These statements and others like it are not true*** for the reasons stated in point #1 above and demonstrated in 10.1109/IUS51837.2023.10307088.

3. In the response to questions 11 and 12 from reviewer 2, the authors state "This is true, but interestingly, when we responded to Reviewer 1's point 13, we calculated the corresponding plot to Figure 2C that included negatively valued pixels and the corresponding plot for the contrast to noise ratio. In this new analysis, we do see a decrease in photoacoustic signal with increasing melanin level, as predicted by optical considerations. We think this happens because the clutter signal is oscillatory (Figure 2B), cancelling out when negative pixels are included, but contributing positively when negative pixels are excluded. The negative component of the clutter labelled on the right above would be excluded under the original analysis, while the positive clutter peak would be included, compensating for the fluence-induced reduction in signal intensity when negative pixels are included." This is interesting, but the standalone statement in the paper about this finding is incorrect. In particular, the statement: "However, the mean signal intensity and corresponding contrast to noise ratio in the radial artery decreased significantly with increasing melanin level when negative pixels were included (Fig. S7)." is not true for the 1080 nm wavelength. While the following sentences try to address this, more attention should be given to ensure that the quoted sentence about Fig. S7 is accurate by adding appropriate nuance and caveats to the standalone sentence.
4. Further to point #3 above, it is not clear why the observed effect about oscillatory clutter is wavelength dependent. It would help if the authors can clarify their proposed theory with respect to this detail.
5. In response to question 5 from reviewer 2, the authors state, "The increased variability appears to be due to a constant percentage variability in the PA signal in the skin surface, which can be seen when plotted on a log scale. Here all imaging sites are included and we fitted a LOWESS smooth fit with bootstrapping to calculate the confidence interval." However, I could not find these details included in the paper. The authors also seem to argue that a high number of patients is more important than addressing the observed differences with the darker skin tones. Note that these are two separate issues (i.e., statistical significance for all participants vs. results that are relevant to one subgroup and not another subgroup), and they should not be pitted against each other. The associated questions were not concerned with statistical significance or the total number of patients.
6. The response to question 21 from reviewer 2 is insightful to address some of the subgroup concerns that were raised, but the negative result for darker skin tones is not clearly presented in the paper.

Part II: Details in the paper that are unclear

7. In the introduction section, the authors reference conflicting results between references 9 and 11. What exactly is the conflict being referred to?
8. "For individuals with very high skin pigmentation, image clutter is shown to originate from the skin surface acting as an ultrasound transducer, with photoacoustic waves generated in the epidermis being backscattered throughout the image field." This may simply be an

oversight, but it's important to mention again that this was already shown by previous investigators. By not citing previous work, I am left with the incorrect impression that the authors are reporting this particular finding for the first time. See Part I comment 1 for more details. **The authors presented several points to explain why their study differs from previous studies, but this critical concern remains unaddressed.**

9. "All participants underwent photoacoustic imaging (PAI) with simultaneous reflection ultrasound (US) computed tomography and colourimetry at all each imaging site." It seems that "all" needs to be removed or otherwise its purpose should be clarified. There are at least 1-2 other typos in the newly added red text, which did not affect overall interpretation.
10. "The regions of interest analysed showed some variation in size and depth (mean ± 164 standard deviation): radial artery diameter 8.5 ± 1.0 mm, depth 2.9 ± 0.8 mm; carotid artery diameter 10 ± 4 mm, depth 15 ± 3 mm. The centroid of the bicep muscle region was at a depth of 14.2 ± 1.4 mm, while that of the calf muscle was 14.3 ± 1.6 mm. Muscle regions of interest included as much of the muscle as was visible in the field of view." This text seems out of place in the results subsection labeled "A diverse healthy volunteer study was vital to understand confounding effects of skin colour in photoacoustic imaging." These are not details that contribute to the subheading and are instead details about the methodology, which would be better placed in the Methods & Materials section to avoid confusion about the main message in this section. If the text stays, a concluding statement about why variations in size and depth affect the understanding of confounding effects needs to be added to the quoted paragraph. The conclusion seems to be that "there was no systematic relationship between ITA and region of interest depth" based on the response to Reviewer 1 comment 11, but no data is presented to support this conclusion.
11. It is not clear if the regions of interest noted above refer to the CNR measurements in addition to the measurements taken to assess signal trends as a function of skin tone. Please clarify.
12. If the regions of interest quoted in point #10 were indeed used to calculate the CNR, for the muscles, it is not clear if the statement "Muscle regions of interest included as much of the muscle as was visible in the field of view." refers to the identified target or background region of interest. In addition, the sizes of the background regions of interest when calculating the CNR of the arteries are not specified. More details are needed to better understand the authors' approach to this calculation.
13. "Unfortunately, this did not compensate for biases observed for darker skin tones, suggesting that spectral colouring becomes a less significant source of bias as melanin levels increase." It is not clear what features of the data presented in Fig. 5 support this conclusion.
14. "Given the strong correlation between ITA and photoacoustic signal (Fig. 1G), we derived a fluence correction factor based purely on the mean photoacoustic signal from the skin surface." When structures from lower ITA values are not visible because of optical penetration issues, it is unlikely that this approach will be sufficient. Perhaps a less than (<) sign missing in the statement "removing a linear ITA dependence for ITA > 10 (p = 0.63) and ITA > 30 (p = 0.85) respectively." If not, why is the linear dependence removed for both ITA>10 and ITA>30? Is this referring to the pre- and post-correction cases discussed in the preceding paragraph? Further, the authors reported Fig. S4, which presents a statistical

significance in these ITA ranges, so the methodology and reported p value discrepancies are unclear here.

15. "To address the acoustic effects, we modelled the epidermis as a source of ultrasound plane waves, allowing us to reconstruct ultrasound images from the clutter signal." The advantage of doing this is unclear. I think the more appropriate message is that the mUS images were made to demonstrate that skin is the source of clutter, but this does not come across clearly based on the way the sentence is written, as the acoustic effects were not "addressed", just demonstrated. In other words, the technique to reconstruct mUS images does not "address" the source of the clutter in the same way that the optical effects were addressed in the preceding sentence. Consider rewriting this sentence to clarify the intent here.
16. "Because of the high optical absorption of melanin, the epidermis acts as an ultrasound transmitter, emitting waves into the deeper tissue, which give rise to reflected ultrasound signals that are far stronger than the desired primary photoacoustic signal from the tissue of interest." A citation to prior references that state the same is warranted here, as this is not a new idea.
17. This is not true of DCA (reference 16) or PAFUSion (reference 12): "but require either specialist hardware to cancel the scattered wave or introduce a non-linear scaling to the intensity making quantification impossible." To be accurate about this, I suggest (a) adding "unique acquisition protocols" to this list and (b) including the appropriate citation(s) next to each limitation, rather than at the end of the sentence. Without suggestion b, it is not clear which limitation the authors are claiming to be associated with each technique cited. Further, note that PAFUSion is primarily concerned with reflection artifacts from highly reflective surrounding structures, which is not the same as the scattering events that cause the acoustic clutter. Therefore, this algorithm is not really relevant here, and my above suggestion does not correct the inaccurate limitation(s) listed for this technique.
18. "Photoacoustic signals from the skin surface region at the lowest wavelength of 700 nm (highest melanin absorption) increased only modestly between non-pigmented regions and pigmented regions, possibly because of absorption from other chromophores like haemoglobin..." Another possibility is that the group of vitiligo patients contains none from the Fitzpatrick IV-VI groups. This should be mentioned more prominently if the authors agree with this probability as stated in the response to the reviewers.
19. The group of vitiligo patients remains limited with none from the Fitzpatrick IV-VI groups, which would present greater variation of skin color for a single participant and would provide a more complete analysis of the claims made with respect to these patients. Why is there no mention of this weakness in the discussion section? An appropriate place to discuss this would be where the following text exists: "Additional variation in skin surface pigmentation, for example, increased pigmentation associated with scar tissue or darker areolar skin in breast imaging, or decreased pigmentation with regression in melanoma imaging, could compound these effects." The extension to scar tissue and breast tissue variations here appears as an unnecessary diversion from the critical detail about skin color variations in vitiligo patients with darker skin tones, which were not included in this study. Please prioritize discussion of this important limitation. This should take priority over the apparent diversion of readers' attention to speculative information about scar tissue and breast tissue applications that cannot be verified.

20. The speculative details about scar tissue, breast tissue, etc. come across as a disconnected distraction from the key results presented in this paper about a very different patient population. Consider this perspective and how to best address this concern, either through removal of said text or through updated text that better justifies its inclusion. Multiple unsupported speculations should really be avoided. Although the authors think this could stimulate future research (as noted in response to Reviewer 2 comment 22), such speculations can also perpetuate inaccuracies if they are not true. Ideally, there would be some initial result demonstrated before such speculations are made in archived literature.

Part III: Minor details

21. References 14 and 34 are duplicates of the same paper.
22. There is insufficient information to locate Reference 31, which only states “Fitzpatrick skin phototype.”
23. A reference to the Monk scale is similarly needed.
24. In response to Reviewer 1 question 8, the authors state: “While addressing this question, we uncovered a single outlier in the ITA measurements of participant 25 (radial artery) by plotting the standard deviation of the five measurements against the mean (see figure below).” However, the referenced figure states the mean was taken over three rather than five measurements. This is confusing.
25. “The overall signal intensity of different structures did not decrease with increasing skin pigmentation, as might be naively predicted...” Remove “naively”. It is not scientifically appropriate to qualify the associated thought this way, and the sentence retains its meaning without this word.
26. “In the bicep, there was a dramatic increase in mean photoacoustic signal with increasing skin pigmentation...” There is a lot of variation in the results presented in Fig. 2D. Given these variations, it is unclear how the reported trends are statistically significant. It is also concerning that the standard deviations in Fig. 2D are larger than some of the mean values in Fig. 2D.
